# In Situ Vivianite Formation in Intertidal Sediments: Ferrihydrite-Adsorbed P Triggers Vivianite Formation

**DOI:** 10.1021/acs.est.4c10710

**Published:** 2024-12-25

**Authors:** L. Joëlle Kubeneck, Katherine A. Rothwell, Luiza Notini, Laurel K. ThomasArrigo, Katrin Schulz, Giulia Fantappiè, Prachi Joshi, Thomas Huthwelker, Ruben Kretzschmar

**Affiliations:** † Soil Chemistry Group, Institute of Biogeochemistry and Pollutant Dynamics, CHN, ETH Zürich, 8092 Zürich, Switzerland; ‡ School of Earth Sciences, University of Bristol, Bristol BS8 1RJ, U.K.; ¶ Department of Civil, Construction, and Environmental Engineering, University of Delaware, Newark, Delaware 19716, United States; § Environmental Chemistry Group, Institute of Chemistry, University of Neuchâtel, 2000 Neuchâtel, Switzerland; ∥ Geomicrobiology, Department of Geosciences, University of Tübingen, 72076 Tübingen, Germany; ⊥ Paul Scherrer Institut, 5232 Villigen, Switzerland

**Keywords:** Mössbauer spectroscopy, phosphorus cycling, coastal sediments, iron minerals

## Abstract

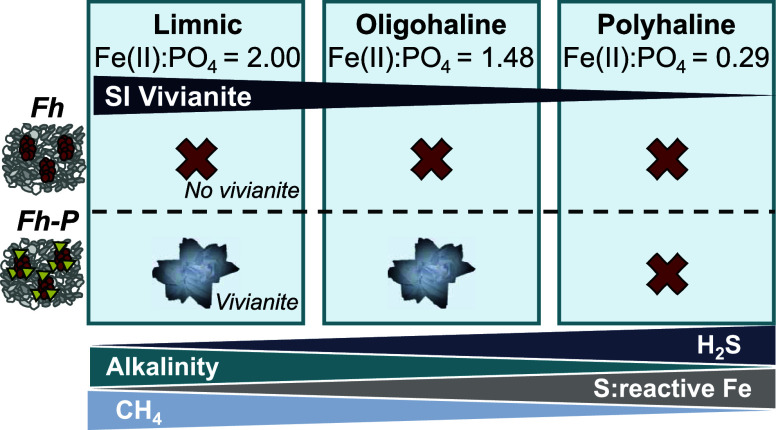

Coastal sediments are a key contributor to oceanic phosphorus (P) removal, impacting P bioavailability and primary productivity. Vivianite, an Fe(II)-phosphate mineral, can be a major P sink in nonsulfidic, reducing coastal sediments. Despite its importance, vivianite formation processes in sediments remain poorly understood. Here, we applied a novel approach to detect and quantify in situ vivianite formation in three intertidal flats. We conducted 7-week long incubations of mesh-bags filled with sediments mixed with (1) ^57^Fe-ferrihydrite, (2) ^57^Fe-ferrihydrite with adsorbed phosphate, and (3) ^57^Fe-ferrihydrite with adsorbed phosphate and some vivianite (natural Fe isotope abundance), which could serve as crystal growth sites. Synthesizing the ferrihydrite from ^57^Fe (96.1%) enabled us to detect transformation products using ^57^Fe-Mössbauer spectroscopy. Vivianite formed only in treatments containing adsorbed phosphate and only at the two sites where vivianite formation was thermodynamically feasible based on porewater chemistry. These results demonstrate vivianite formation within weeks when locally favorable Fe:P ratios exist. Although vivianite comprised a minor fraction of Fe (up to 15%), it represented a significant P pool (up to 72%), emphasizing its role in coastal P burial. Additionally, our results may apply to other environmental systems like limnic sediments.

## Abstract 2

Adopting a novel approach to track iron mineral transformations in situ, we detect in situ vivianite formation under favorable conditions, highlighting its crucial role in phosphorus burial in coastal sediments.

## Introduction

Riverine runoff of fertilizers and sewage increases terrestrial phosphorus (P) inputs to coastal oceans globally, fueling eutrophication^1^ and resulting in the spreading of dead zones (hypoxic to low-oxygen zones).^2^ This impacts ecosystem functioning and is anticipated to intensify with climate change.^2^ The retention and burial of P in coastal sediments regulate the availability of P in the water column through processes such as sorption to minerals like iron (oxyhydr)oxides (Fe-oxides), precipitation within authigenic minerals, and burial of organic matter.^3^ Until recently, vivianite, a ferrous phosphate mineral (Fe_3_(PO_4_)_2_·8H_2_O), was considered a minor contributor to P burial in coastal sediments.^3,4^ However, accumulating field observations challenge this notion.^4−10^ Recent estimates propose that vivianite sequesters up to 50% of total P in sediments of the Bothnian Sea and the Chesapeake Bay,^4,6,9,10^ highlighting its potential role in coastal P removal and thus overall oceanic P cycling. Vivianite predominantly forms in low sulfate environments enriched in Fe-oxides, commonly found in low-saline coastal environments and below the sulfate-methane transition zone (SMTZ) in marine sediments.^4,5,9^

Despite the importance of vivianite for long-term P retention, open questions remain regarding in situ formation kinetics, the role of nucleation sites, and the role of precursor phases.^7^ For instance, Fe-oxides with adsorbed phosphate have been suggested as a precursor phase,^4,11,12^ supported by a recent study showing vivianite formation within 4 weeks in lake sediments amended with Fe-oxides preadsorbed with phosphate.^13^ However, this study was conducted ex-situ and utilized Fe-oxide with adsorbed phosphate with a lower Fe:P (3.7) ratio than the commonly reported ratio of 10 for coastal and marine sediments.^14,15^ Other work^16^ did not observe vivianite formation during the in situ incubation of gel samplers containing ferrihydrite coprecipitated with phosphate in Fe- and P-rich sediments, suggesting that transformation was inhibited by surface passivation by adsorbed phosphate. These contrasting findings highlight an incomplete understanding of a potential precursor for vivianite formation.

While thermodynamic calculations frequently predict vivianite occurrence, crystalline vivianite is often undetected in natural samples.^7^ This absence may indicate slow in situ nucleation and crystal growth kinetics. In situ formation kinetics can be accelerated by the presence of suitable nucleation and crystal growth sites.^17^ However, research on the role of nucleation sites for vivianite formation remains limited. Conversely, the absence of detected vivianite crystals could also be linked to methodological limitations, challenging the identification and quantification of vivianite in natural samples.^4,6,7,18^ For instance, commonly applied wet chemical extractions are not mineral-specific,^6^ and detection and quantification by P K-edge X-ray absorption near edge structure (XANES) spectroscopy can be challenging, due to similar spectral features in different P compounds and the sensitivity of linear combination fitting to the “white line” magnitude (the maximum following the absorption edge).^4,8^ Furthermore, the vivianite content is often below the detection limit of bulk techniques like X-ray diffraction (XRD) analysis and for Fe-specific methods such as Fe K-edge extended X-ray absorption fine structure (EXAFS) spectroscopy.^4,6,19^ Therefore, a novel approach is needed to detect in situ vivianite formation.

Here, we adapted and modified an approach demonstrated by Notini et al.^20^ to study in situ vivianite formation at three intertidal flats along the Elbe estuary in Northern Germany. The three sites encompass a range of solid-phase Fe:P:S ratios (Table 1), offering conditions to explore geochemical parameters influencing vivianite formation. Mesh-bags containing either (i) ^57^Fe-ferrihydrite (Fh treatment), (ii) ^57^Fe-ferrihydrite with adsorbed phosphate (FhP treatment), or (iii) ^57^Fe-ferrihydrite with adsorbed phosphate and vivianite (FhP+Viv treatment; vivianite synthesized with natural Fe isotope abundance), mixed with sediment were incubated for 7 weeks at 10–15 cm sediment depth at each site. The ferrihydrite in each treatment was strongly enriched in ^57^Fe (96.1% compared to 2.1% natural abundance^21^), resulting in ∼95% contribution of the added ^57^Fe-ferrihydrite to the total ^57^Fe pool. This allowed us to track transformation products using ^57^Fe-Mössbauer spectroscopy while mimicking natural sediment conditions, as the added mineral was finely dispersed in the sediment matrix and exposed to natural porewater conditions. The FhP treatment assessed the importance of a precursor phase, while vivianite addition tested whether providing crystal growth sites could accelerate formation kinetics. Our results demonstrate vivianite formation within 7 weeks only from FhP(+Viv) treatments under favorable environmental conditions, providing novel insights into in situ formation, the role of a precursor, and vivianite’s importance for coastal P burial.

**Table 1:** Elemental Composition and Ratios of the Unamended Sediment and Experimental Treatments (Fh, FhP, FhP+Viv) for Each Field Site before Field Incubation^a^^,^^b^

			HSF	HW	FKS
unamended sediment	total S	[μmol/g]	157	187	104
	total Mn	[μmol/g]	21	35	5
	total Fe	[μmol/g]	331	557	193
	reactive Fe	[μmol/g]	142	248	46
	total S:reactive Fe	[mol/mol]	1.10	0.75	2.26
	total P	[μmol/g]	46	48	26
	reactive Fe:total P	[mol/mol]	3.09	5.17	1.77
Fh treatment	added Fh per mesh-bag	[mg]	13.0	22.6	7.8
	^57^Fe from added Fh	[%]	94.7	94.7	94.7
	increase in total Fe with Fh addition	[%]	38.7	38.6	40.5
	total Fe	[μmol/g]	458	772	271
	total P	[μmol/g]	46	48	26
	reactive Fe	[μmol/g]	270	463	124
	total S:reactive Fe	[mol/mol]	0.58	0.40	0.84
	reactive Fe:total P	[mol/mol]	5.87	9.64	4.78
FhP treatment	added FhP per mesh-bag	[mg]	14.5	25.1	8.7
	^57^Fe from added FhP	[%]	94.8	94.8	94.8
	increase in total Fe with FhP addition	[%]	38.7	38.3	40.6
	total Fe	[μmol/g]	458	771	271
	increase in total P with FhP addition	[%]	39	63	42
	total P	[μmol/g]	63	78	37
	reactive Fe	[μmol/g]	270	462	125
	total S:reactive Fe	[mol/mol]	0.58	0.41	0.83
	reactive Fe:total P	[mol/mol]	4.27	5.90	3.36
FhP+Viv treatment	added FhP per mesh-bag	[mg]	14.5	25.1	8.7
	^57^Fe from added FhP	[%]	94.2	94.5	94.2
	added vivianite per mesh-bag	[mg]	5.2	8.7	3.0
	increase in total Fe due to mineral addition	[%]	48	47	50
	total Fe	[μmol/g]	489	818	289
	increase in total P due to mineral addition	[%]	81	128	184
	total P	[μmol/g]	82	110	48
	reactive Fe	[μmol/g]	300	509	143
	total S:reactive Fe	[mol/mol]	0.52	0.37	0.73
	reactive Fe:total P	[mol/mol]	3.65	4.64	2.97

a Elemental concentrations were determined by X-ray fluorescence, and reactive Fe is the sum of the first five steps of the sequential Fe extraction.

b Abbreviations: HSF = Haseldorfer Marsch, HW = Hollerwetter, FKS = Friedrichskoog.

## Material and Methods

### Field Sites

**Figure 1:**
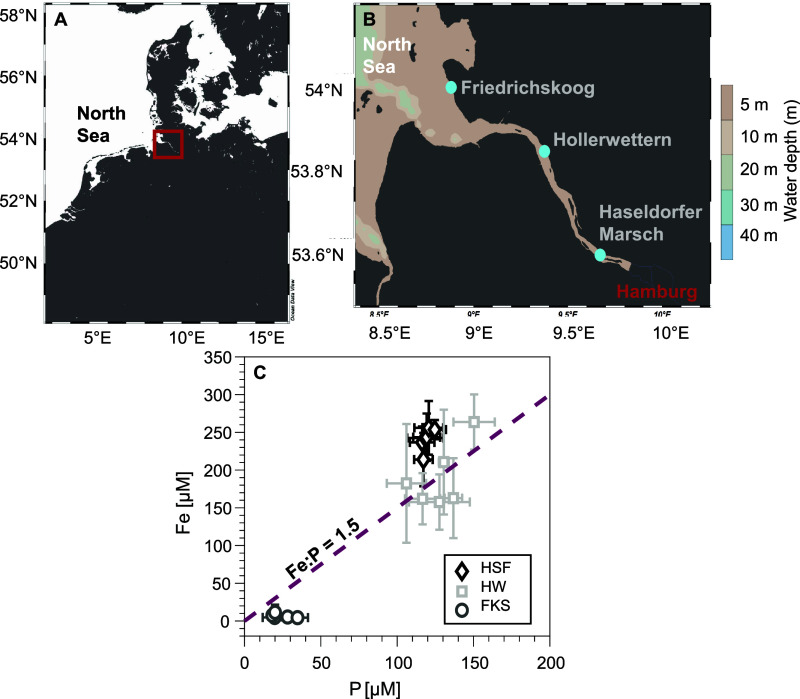
Location of the Elbe estuary (A) and the three field sites along the estuarine gradient (B). Dissolved Fe and P concentrations at 12.5 cm sediment depth are shown for each site (C). Porewater was collected in triplicate and analyzed 5–6 times during the experiment. Figure C shows mean concentrations with error bars representing standard deviation. Dashed line indicates the Fe ratio of 1.5 (mol:mol), the theoretical value for vivianite. Figures A and B were created with Ocean Data View.^63^ Abbreviations: HSF = Haseldorfer Marsch (low salinity), HW = Hollerwettern (medium salinity), FKS = Friedrichskoog (high salinity).

The three field sites are situated along the Elbe estuary, Northern Germany (Figure 1A). The Elbe estuary is classified as eutrophic and characterized by high nutrient and particulate matter inputs.^22^ Study site Haseldorfer Marsch (HSF; 53°34′50″ N, 9°39′27″ E) is located in the upper part of the estuary (<0.5 practical salinity unit (psu), limnic conditions), while study site Hollerwettern (HW; 53°49′36″ N, 9°22′59″ E) is situated in the middle estuary with oligohaline conditions (0.5–5 psu). The third study site, Friedrichskoog (FKS; 54°0′42″ N, 8°50′6″ E; Figure 1B), is characterized by polyhaline conditions (>18 psu) and situated in the lower estuary. The intertidal flats were chosen to include a range of salinities, as well as different sedimentary Fe, P, and S solid-phase contents (Table 1) and aqueous geochemical conditions (Figure 1, Section S3), providing ideal conditions to study how geochemical conditions across an estuarine gradient impact vivianite formation.

### Experimental Treatments

Three treatments were tested to investigate factors potentially controlling vivianite formation. Three mineral phases were prepared for these treatments: ^57^Fe-ferrihydrite, ^57^Fe-ferrihydrite with adsorbed phosphate, and vivianite. ^57^Fe-ferrihydrite was synthesized from an oxidized ^57^Fe-solution and using 1 M NaOH as a base, following the method presented by Notini et al.^20^ (see Section S1.1). One batch of the ^57^Fe-ferrihydrite suspension was spiked with 26.6 mL of a 0.06 M Na_2_HPO_4_ solution to prepare ^57^Fe-ferrihydrite with adsorbed phosphate. The reaction was allowed to proceed for 24 h at constant pH 6.5 ± 0.2 (readjusted if needed, see Section S1.1). The final solid-phase Fe:P ratio was 7.1 (Section S2). Vivianite was synthesized from stock solutions of FeSO_4_ and Na_2_HPO_4_ in an anoxic chamber (MBraun, UNIlab PLUS, N_2_ atmosphere, <1 ppm (v/v) O_2_) following the protocol of Kubeneck et al.^23^

The dried and homogenized minerals were used to prepare different sediment-mineral mixes using each field site’s respective dried (30 °C, ambient atmosphere), sieved (<2 mm), and homogenized sediment (further details Section S1.2). The following treatments were prepared: Fh—sediment mixed with ^57^Fe-ferrihydrite; FhP—sediment mixed with ^57^Fe-ferriydrite with adsorbed phosphate; FhP+Viv—sediment mixed with ^57^Fe-ferriydrite with adsorbed phosphate and vivianite; and Control—sediment without mineral additions. For preparing the treatments, the sieved and homogenized sediment was mixed with the mineral phase(s) in a centrifuge tube (5 mL) and well shaken by hand for 1 min to ensure homogeneous mixtures. The mass of spiked ^57^Fe-ferrihydrite, ^57^Fe-ferrihydrite with adsorbed phosphate, and vivianite depended on the initial Fe content of the sediment (Table 1). The spiked mass of ^57^Fe-ferrihydrite or ^57^Fe-ferrihydrite with adsorbed phosphate (e.g., 13 mg/g ^57^Fe-Fh for HSF sediment) was chosen to ensure that the added ^57^Fe in the form of ferrihydrite contributes ∼95% to the spectral area of Mössbauer spectra as ^57^Fe-Mössbauer spectroscopy is only sensitive to the ^57^Fe isotope. For all sediments, this addition resulted in a ∼40% increase in Fe content (Table 1). The added amount of Fe in vivianite was equivalent to 10% of the original sediment Fe content (e.g., 5.2 mg/g vivianite for HSF sediment), ensuring that the added vivianite would be mostly invisible to ^57^Fe-Mössbauer spectroscopy (<1% contribution to Mössbauer signal) and below the detection limit of Fe K-edge EXAFS analysis (final vivianite contribution ≤6% to total Fe). The sediment-mineral mixes for the FhP+Viv treatment were prepared inside an anoxic chamber to prevent vivianite oxidation. Table 1 provides an overview of how mineral additions changed elemental contents and ratios in each treatment.

The sediment-mineral mixes (1 g) were filled into 5 cm long and 1.5 cm wide polyethylene terephthalate mesh-bags (51 μm pore size, SEFAR, Switzerland, Figure S1). To minimize solid phase material loss through the mesh, mesh-bags comprised three mesh layers. The filled mesh-bags were heat sealed and then placed into 3D-printed acrylic sample holders with a 5 cm long opening, matching the mesh bag dimensions (Figure S1). A threaded labeled nylon rod was screwed onto the sample holder containing the sample to allow easy insertion and retrieval of the samplers in the field (Figure S1). The prepared sample holders with mesh-bags of Fh, FhP, and Control treatment were vacuum-sealed and transported to the field, while sample holders with mesh-bags of FhP+Viv treatment were prepared in an anoxic chamber and transported to the field in airtight, double-sealed, N_2_-flushed Al-bags.

### Experimental Setup

At each field site, 10 samples (Fh, FhP, FhP+Viv in triplicate and one Control) were installed in the sediments at an equivalent distance from each other along the circumference of a circle (∼2 m diameter) in Summer 2021 (July to September). Samples were inserted into the sediment, ensuring that the sample holder window was located at 10–15 cm depth. This depth was chosen since porewater analysis in previous years indicated the highest concentration of dissolved Fe and P at this depth, while dissolved sulfide was not detected (data not shown). To install the samples, the samples were removed from the vacuum-sealed bag and pushed into the sediment (Figure S1). To prevent vivianite oxidation in the FhP+Viv samples, a 15 cm long core liner (UWITEC, PVC corer, 8.6 cm diameter) was inserted 2 cm into the sediment. The headspace of the core liner was flushed with N_2_ for about 3 min. Sample holders with FhP+Viv were removed from airtight Al-bags and swiftly inserted into the sediment under the N_2_ atmosphere.

At the end of the experiment, after 7 weeks, all samples were still in place. Samples were pulled out of the sediment with the help of the nylon rod and immediately put into vacuum-sealed bags and stored on ice (Figure S1). Within 5 h, samples were sealed in airtight, N_2_-flushed double-sealed Al-bags and frozen at −20 °C. Samples were transported frozen back to the laboratory at ETH Zürich, where samples were thawed and dried in an anoxic glovebox. Surrounding sediment was removed from the samplers, and they were carefully broken apart to recover the mesh-bags containing the reacted solid-phase. Subsequently, the reacted solid phase was gently homogenized with an agate mortar and pestle and stored in airtight, amber glass vials in the anoxic chamber (Figure S1).

### Porewater Characterization

The detailed methodology of porewater collection and analysis is provided in Section S1.4. Approximately every 7–10 days during the field experiment, sediment temperature (at ∼10 cm depth) and oxidation–reduction potential (ORP) were measured within the experimental plots during low tide (±3 h). ORP was determined with a custom-made ORP probe with Pt electrodes at 10 and 15 cm depth and an AgCl-reference electrode (supersaturated KCl, Paleo Terra, The Netherlands). Measured ORP values were converted to redox potentials (Eh) relative to the standard hydrogen electrode (+204 mV at 20 °C^24^). Additionally, porewater samples for major elemental and anion analysis, as well as pH, alkalinity, and hydrogen sulfide, were taken in triplicate from three locations within the experimental plot with MacroRhizons (5 cm long porous part, outer diameter 4.5 mm, 0.15 μm pore size, Rhizosphere, The Netherlands) at a sediment depth of 10–15 cm. After ∼5 weeks into the experiment, one sediment core (∼40 cm length, 8.6 cm diameter) was recovered for depth-resolved methane (CH_4_) analysis at each field site (see Section S1.4.2). At the end of the experiment, six additional sediment cores per field site were taken to determine pH, alkalinity, and major anion and elemental porewater depth profiles.

Porewater samples for pH and alkalinity were processed within 8 h after porewater collection. Alkalinity was determined via a two-step titration (Titrimetric test kit, VISOCOLOR HE Carbonate hardness, Macherey-Nagel). Samples for major anion analysis were frozen at −20 °C until analysis with ion chromatography (IC, Metrohm 040 Professional IC Vario). Samples for hydrogen sulfide species were preserved by adding zinc acetate and cooled until spectrophotometrical measurements.^25^ Samples for elemental analysis were acidified and cooled until analyzed by inductively coupled plasma optical emission spectroscopy (ICP-OES, Agilent 5100). CH_4_ concentrations were determined by gas chromatography (GC, TraceGC1300, ThermoFisher Scientific, modified by S+HA analytics) and corrected for sediment porosity. The porewater data was used to calculate the saturation indices of the porewater with respect to vivianite and siderite using Visual MINTEQ (Version 3.1).

### Initial and Reacted Solid-Phase Analysis

^57^Fe-Mössbauer spectra were collected for a subsample of dried initial and reacted (triplicates combined) samples at 77, 25, 13, 10, and 5 K (see Section S1.5.2) and analyzed by extended Voigt-Based fitting (xVBF) routine^26^ or Full Static Hamiltonian (FHS) fitting routine for 5 K spectra^20^ using Recoil software (University of Ottawa, Canada). Triplicates were combined as sequential Fe extraction showed little heterogeneity among replicates (Section S4.1). To gain further insights into the bulk Fe geochemistry of the solid-phase Fe, hand-milled subsamples of the reacted solid phases (triplicates combined) were prepared into pellets to collect Fe K-edge X-ray absorption spectroscopy (XAS) data. Fe K-edge XAS data were normalized and fitted by linear combination fitting (LCF) with reference spectra in Athena^27^ (see Section S1.5.3). In addition, hand-milled samples of FhP (triplicates combined) and Control samples were mounted on double-sided carbon tape to collect bulk P K-edge X-ray absorption near-edge structure (XANES) spectra to gain insights into bulk P mineralogy (see Section S1.5.4).

## Results and Discussion

### Geochemical Indicators for Vivianite Formation

We monitored porewater composition and characterized the solid-phase geochemistry at each site to understand if geochemical conditions favored vivianite formation. Porewater analysis showed little variations in geochemistry over the 7-week incubation at the three field sites and indicated ongoing anaerobic respiration at 10–15 cm depth. Redox potential ranged between 50 and 100 mV at the low and medium salinity sites HSF and HW, and 50–150 mV at high salinity site FKS (Figure S7, Section S3). At the low and medium salinity sites (HSF and HW) the dissolved Fe and P concentrations were high (125–300 and 100–150 μM, respectively; Figure 1C). Dissolved Fe:P molar ratios were mostly around 1.5 (Figure 1C), suggesting sufficient Fe availability for vivianite formation, which theoretically requires a stoichiometric ratio of 1.5.^23^ Furthermore, sulfate was rapidly depleted over depth, resulting in shallow SMTZs (5–10 cm depth; Figures S12–S15). High aqueous Fe concentrations below the SMTZ suggest ongoing Fe-reduction, potentially coupled with anaerobic methane oxidation,^28^ creating conditions where dissolved Fe exceeds the sulfide scavenging capacity. These conditions favor vivianite formation,^4,10^ consistent with thermodynamic calculations indicating the oversaturation of the porewater with respect to vivianite at 10–15 cm depth (Figures S6 and S16). Furthermore, the total S to reactive Fe solid-phase ratios (≤1.1) were low at HSF and HW (Table 1). A ratio below 1.1 has been associated with favorable conditions for vivianite formation.^10,18^ Collectively, vivianite formation was expected at both sites considering geochemical conditions. This hypothesis was further supported by the presence of vivianite in control samples (incubated unamended sediment). While vivianite was not detected by Mössbauer spectroscopy nor Fe K-edge XAS in the initial and reacted unamended sediment (Sections S4.2.4 and S4.4), P K-edge XANES analysis indicated that vivianite comprised 21–24% of the total P pool in the reacted unamended sediment (Tables 2 and S23).

**Table 2:** Comparison of the Contribution of Vivianite to the Total Fe and P Pool Using ^57^Fe-Mössbauer Spectroscopy, Fe K-Edge EXAFS, and P K-Edge XANES in Reacted Samples^a^

sample	^57^Fe-Mössbauer spectroscopy				Fe K-edge EXAFS		P K-edge XANES	
	^57^Fe in Viv^b^ (%)	Fe in Viv^c^ (%)	P in Viv^d^ (%)	added P bound in Viv^e^ (%)	Fe in Viv (%)	P in Viv^f^ (%)	Fe in Viv^g^ (%)	P in Viv^h^ (%)
HSF control	ND				ND		5	24
HSF FhP	41	11	53	191	ND		4	19
HSF FhP+Viv	35	11	43	97	ND			
HW control	ND				ND		3	21
HW FhP	27	7	48	123	11	70	4	24
HW FhP+Viv	36	11	56	100	15	72		

a Abbreviations: ND = not detected.

b Assuming a 1:2 stoichiometry between Fe(II)D1:Fe(II)D2 in vivianite, the spectral area of Fe(II)D2 was used to calculate the total contribution of vivianite to the remaining ^57^Fe-pool.

c Assuming no change in total Fe or ^57^Fe during incubation, we converted the quantity of ^57^Fe present in vivianite into its contribution to the total Fe pool.

d Assuming a 3:2 Fe:P molar ratio in vivianite, we converted its contribution from the total Fe pool to the total P pool while assuming no change in the total P concentration during incubation.

e We added P through FhP addition and calculated how much of the initially added P was theoretically present in vivianite. Values >100% indicate that more P was present in vivianite than we initially added to our treatment.

f Assuming a 3:2 Fe:P molar ratio in vivianite, we converted its contribution from the total Fe pool to the total P pool while assuming no change in the total P concentration during incubation.

g Assuming a 3:2 Fe:P molar ratio in vivianite, we converted its contribution from the total P pool determined by LCF of P K-edge XANES to the total Fe pool while assuming no change in the total Fe concentration during incubation.

h Modeled by unsubstituted vivianite (Table S23).

In contrast, the high salinity site FKS was characterized by low dissolved Fe and P concentrations (0–40 μM for both elements), with low dissolved Fe:P ratios (<1.5; Figure 1C). Additionally, sulfate concentrations were high (14.5–21.6 mM), and sulfide was detected (Figure S10), indicating conditions unfavorable for vivianite formation, aligning with the undersaturation of the porewater with respect to vivianite (Figures S6 and S16). The solid-phase geochemistry was characterized by a higher total S to reactive Fe ratio (2.26, Table 1), indicative of an excess of S over reactive Fe. These conditions are not conducive to vivianite formation, consistent with the absence of vivianite in the control sample based on P K-edge XANES analysis (Table S23). Therefore, vivianite formation was not expected at FKS.

### Metastable Mixed-Valence and Fe(II) Minerals as Major Transformation Products

**Figure 2:**
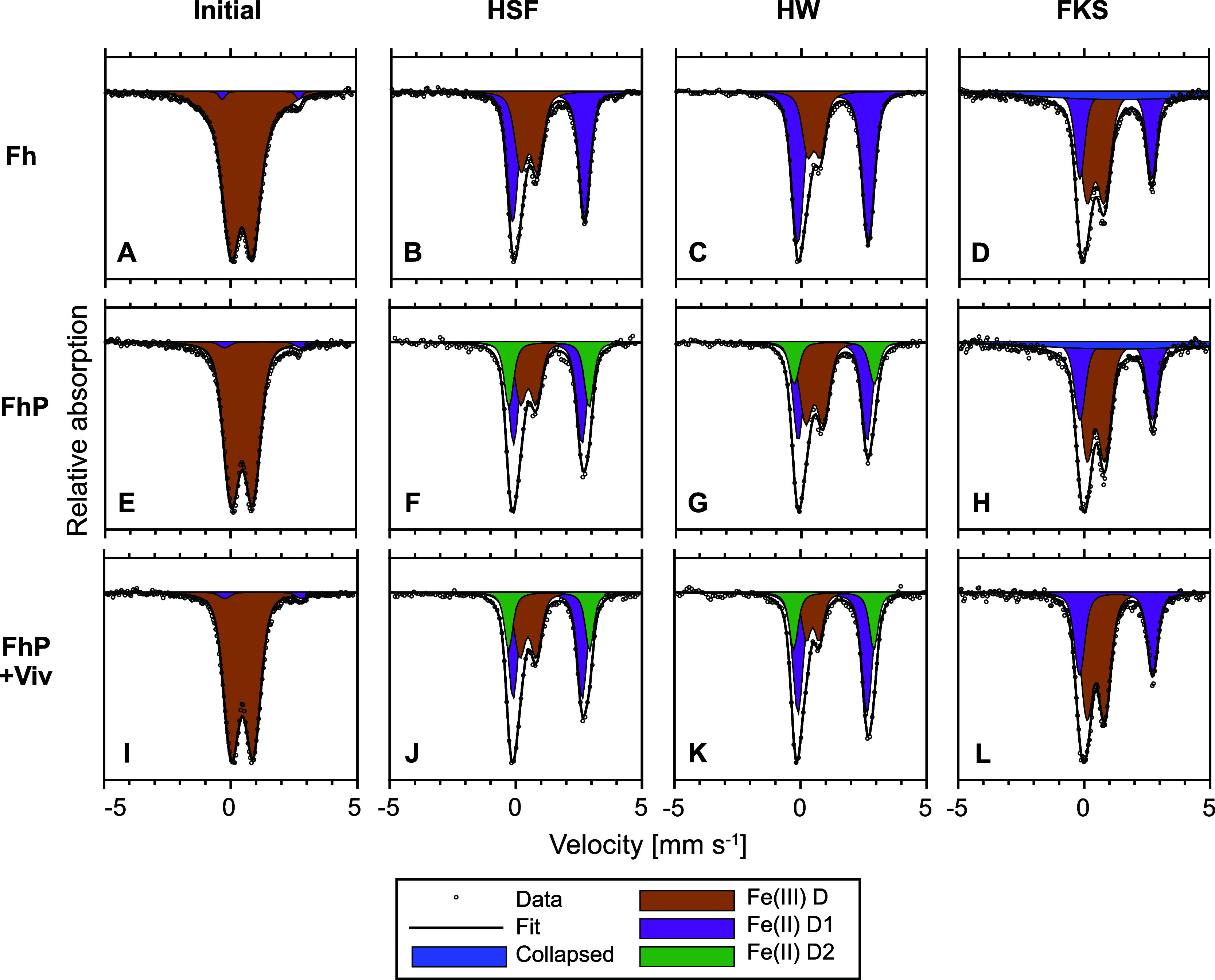
Normalized and fitted ^57^Fe-Mössbauer spectra at 77K for Fh, FhP, and FhP+Viv from initial HSF samples (AEI) and reacted samples at HSF (BFJ), HW (CGK), and FKS (DHL). Additional spectra at 25, 13, 10, and 5 K are shown in Figures S18–S20, with fitted hyperfine parameters in Section S4.2. Abbreviations: HSF = Haseldorfer Marsch (low salinity); HW = Hollerwettern (medium salinity); FKS = Friedrichskoog (high salinity); Fh = sediment + ^57^Fe-ferrihydrite; FhP = sediment + ^57^Fe-ferrihydrite + phosphate; FhP+Viv = sediment + ^57^Fe-ferrihydrite + phosphate + vivianite; Fe(III) D = solid-phase Fe(III) (Fe-oxides or green rust); Fe(II) D1 = solid-phase Fe(II) (green rust, siderite, clay, vivianite, or adsorbed Fe(II)); Fe(II) D2 = Fe(II) in vivianite’s double octahedral position; Collapsed = amorphous Fe-sulfides.

^57^Fe-Mössbauer spectra were collected for initial and reacted samples at five temperatures (77, 25, 13, 10, 5 K) to follow the transformation of the ^57^Fe-labeled ferrihydrite based on mineral-specific fitting parameters or Néel temperatures (Figures 2, S18–S20, Sections S5, S4.2). Initial samples exhibited similar ^57^Fe-Mössbauer spectra since ∼95% of the Mössbauer signal originated from the added ^57^Fe-labeled ferrihydrite (Figures S33 and S34). For simplicity, only initial ^57^Fe-Mössbauer spectra of HSF treatments were plotted in Figure 2 for comparison with reacted samples. At 77 K, the ^57^Fe-Mössbauer spectra of initial Fh and FhP(+Viv) samples consisted of a doublet (Fe(III)D) consistent with ferrihydrite starting to undergo magnetic ordering.^20^

^57^Fe-Mössbauer spectra of reacted Fh samples differed substantially from initial samples across all field sites (Figure 2A–D). At 77 K, a second doublet (Fe(II)D1) with a larger center shift and quadrupole splitting was present, indicating ferrihydrite reduction and the formation of Fe(II) (Tables S5–S13). The Fe(II) doublet contributed 62, 74, and 51% to the spectral area at HSF, HW, and FKS, respectively (Table S21). Based on fitted parameters (Section S4.2, Tables S5–S13), the formed Fe(II) likely comprised a mixture of green rust, adsorbed Fe(II), and siderite at the low and medium salinity sites (HSF and HW). The appearance of an octet below 5 K in the ^57^Fe-Mössbauer spectra corresponds to the magnetic ordering temperature of Fe(II) bound in green rust (Figures S18–S20).^29^ Furthermore, Fe K-edge EXAFS analysis, which identifies changes in the bulk Fe speciation, also confirmed the presence of green rust (Table S18). The formation of siderite was indicated by the emergence of a collapsed feature and a reduction of the Mössbauer spectral contribution of the Fe(II)D1 doublet at 25 K in comparison to 77 K, consistent with the Néel temperature of siderite (Section S4.6).^30^

At FKS, the high salinity site, the presence of a collapsed feature at 77 K in the Mössbauer spectra suggested the formation of Fe-sulfide minerals in addition to green rust and siderite, which was consistent with the analysis of the bulk Fe speciation by Fe K-edge EXAFS analysis. Across all sites, the remaining Fe(III) likely comprised poorly crystalline Fe-oxides (ferrihydrite and/or lepidocrocite) and green rust, as indicated by the presence of sextet(s) at and below 13 K in the ^57^Fe-Mössbauer spectra (Figures S18–S20).^20,29,31^ Interestingly, minimal or no crystalline Fe-oxide formation, such as goethite, was observed in reacted Fh samples across all sites. This contrasts with previous studies using Fe(II)-spiked ferrihydrite suspensions^32,33^ or microbial-driven Fe reduction experiments.^34,35^ Instead, green rust formed similar to recent results by Notini et al.^20^ The formation of metastable green rust was likely promoted and stabilized by various organic and inorganic ligands, such as phosphate, silicate and dissolved organic carbon, present in the porewater,^20,36−41^ while some of those ligands simultaneously hampered crystalline Fe-oxide formation.^42−44^

Although vivianite formation was expected based on thermodynamic calculations at HSF and HW, the ^57^Fe-Mössbauer spectra of the field-reacted Fh samples showed no evidence of vivianite formation from the added ^57^Fe-labeled ferrihydrite. Ferrihydrite addition to the sediment altered solid-phase geochemical conditions (Table 1), by increasing solid-phase reactive Fe:P ratios compared to unamended sediment (e.g., unamended sediment at HSF: 3.1; Fh treatment: 5.9; Table 1). The changed solid-phase ratio likely resulted locally in higher aqueous Fe:P molar ratios upon reductive dissolution in Fh samples than in unamended samples, shifting thermodynamic equilibria toward siderite and green rust.^11,36,38,45^ We hypothesize that the higher solid-phase reactive Fe:P ratios within the mesh-bags explain the absence of vivianite in Fh samples at HSF and HW. The high salinity site (FKS) exhibited higher sulfate concentrations, characteristic of marine sediments.^10,46,47^ The available sulfate likely facilitated microbially driven sulfate reduction,^46^ explaining the presence of Fe-sulfides at FKS.

Our findings indicate that direct contact between ferrihydrite and a reducing sediment matrix favors reductive dissolution promoting the formation of metastable mixed-valence or Fe(II)-minerals such as green rust, siderite, and FeS_*x*_. This suggests a broader occurrence of these minerals in reducing sedimentary environments characterized by higher Fe:P solid-phase ratios, with potential implications for associated elemental cycles, including P. Green rust, known to effectively adsorb phosphate,^41,48^ could regulate P availability in reducing conditions, relevant for current environmental systems as well as conditions during past geological periods such as Precambrian oceans.^49,50^

### Vivianite: A Minor Fe Phase Represents a Major P Sink

Similar to Fh samples, reacted FhP(+Viv) samples contained 38–81% of remaining ^57^Fe as Fe(II) (Table S21), indicating comparable extents of reductive dissolution of ^57^Fe-labeled ferrihydrite in the presence and absence of adsorbed P (Supporting Information S4.6). However, the presence of P changed transformation products at the low and medium salinity sites (HSF and HW). At HSF and HW, P adsorption to ferrihydrite triggered vivianite formation in FhP(+Viv) samples, indicated by the presence of an Fe(II) doublet (Fe(II)D2) in the 77 K ^57^Fe-Mössbauer spectra (Figure 2, Supporting Information S4.2). The Fe(II)D2 hyperfine parameters are consistent with Fe atoms located in the double octahedral position of vivianite.^23^ Theoretically, one-third of the Fe atoms in vivianite are situated in an isolated octahedral position, while the remaining Fe atoms are in a double octahedral position.^23^ Thus, using the spectral area contribution of Fe(II)D2, we calculated vivianite’s contribution to the remaining solid-phase ^57^Fe and total Fe pool (Table 2). The calculation resulted in vivianite comprising 41 and 27% of the total remaining ^57^Fe pool in reacted FhP samples and 35 and 36% in the reacted FhP+Viv samples at HSF and HW, respectively (Table 2). Based on those results, adding vivianite particles (FhP+Viv) as crystal growth sites had a minimal impact on the amounts of formed vivianite. The calculated percentages correspond to 11% of the total Fe present as vivianite at HSF for both FhP and FhP+Viv samples, and 7 and 11% at HW for FhP and FhP+Viv samples based on ^57^Fe-Mössbauer spectroscopy (Table 2). Fe K-edge EXAFS analysis confirmed the presence of vivianite in HW samples (11 and 15% of Fe for FhP and FhP+Viv, respectively). In contrast, Fe K-edge EXAFS analysis of HSF samples could not unequivocally detect vivianite, possibly due to a lower signal-to-noise ratio compared to HW.

The contribution of vivianite to the total Fe pool based on ^57^Fe-Mössbauer spectroscopy can be converted to its contribution to the total P pool (Table 2). At HSF and HW, vivianite represented 7–11% of total solid-phase Fe based on Mössbauer spectroscopy, corresponding to 43–56% of total P (Table 2). However, P K-edge XANES analysis of FhP samples indicated less P in vivianite (19 or 24% at HSF and HW, respectively, Table 2). These discrepancies may result from external P additions, increasing the total P content in the mesh-bag. The Mössbauer calculation uses the original P content (Table 1), while P K-edge XANES measures the actual bulk composition. This could explain why the estimated vivianite pool was smaller based on P K-edge XANES compared to the Mössbauer calculation. Additionally, the discrepancy may stem from analytical challenges, like the sensitivity of LCF of P K-edge XANES to the “white line” magnitude, posing challenges for quantifying the vivianite pool.^4,8^ Furthermore, substitutions in vivianite, altering stoichiometry between the spectral area of ^57^Fe-Mössbauer doublets and features of Fe K-edge EXAFS and P K-edge XANES spectra^51^ could explain variations in the estimates.

Our results offer new insights into the P retention capacity of vivianite, the role of a precursor phase, and the importance of vivianite for coastal P burial. Mass balance calculations indicate that the formed vivianite likely accumulated substantial P from surrounding porewater, underscoring vivianite’s potential role in sequestration and immobilization of bioavailable P (Table 2). For example, in HSF, the addition of FhP increased P content by 17 μmol/g (Table 1). Based on mass balance calculations, the vivianite formed in reacted FhP samples contained ∼34 μmol/g (contributing 53% to total P, Table 2), implying P uptake from the surrounding environment. Thus, our findings indicate the potential importance of vivianite formation in regulating P bioavailability and its likely impact on overall water quality.^13,19^

Vivianite formation was observed within 7 weeks in FhP(+Viv) treatments under suitable geochemical conditions. Previous work by Walpersdorf et al.^52^ reported no vivianite formation in soil slurry experiments after 120 days despite porewater supersaturation indices of 6, concluding slow formation kinetics. In contrast, our findings suggest faster formation kinetics, consistent with Heinrich et al.,^13^ who observed formation within days to weeks in Fe-amended lake sediments. This time scale suggests unhindered nucleation in situ, consistent with our observation that the addition of vivianite particles (FhP+Viv treatment) minimally affected the formed vivianite pool. We hypothesize that various minerals, organic matter, and bacteria in the sediment matrix likely served as nucleation sites for vivianite. However, due to the lack of temporal data we cannot exclude a possible effect of adding vivianite crystals on the initial formation kinetics in the early phase of the experiment.

Our results further suggest that the reductive dissolution of ferrihydrite with adsorbed P played a crucial role in triggering vivianite formation. The reductive dissolution likely resulted in the simultaneous release of Fe(II) and P into the porewater, creating microenvironments with ideal dissolved Fe:P ratios or colloids, triggering vivianite formation. The FhP addition barely altered the solid-phase reactive Fe:P ratio in comparison to the unamended sediment (Table 1), suggesting that similar aqueous Fe:P molar ratios may have persisted upon reductive dissolution. Thus, adding ferrihydrite with adsorbed P likely did not alter thermodynamic equilibria. Collectively, vivianite can form within weeks in situ when reactive (solid-phase and aqueous) Fe:P ratios were locally favorable.

Here, we tested P adsorbed to ferrihydrite as a precursor. Ferrihydrite forms upon Fe(III) hydrolysis^53^ and has a high affinity for P adsorption and incorporation.^16^ Thus, P associated with ferrihydrite, whether adsorbed or coprecipitated, is ubiquitous in soils and sediments, potentially serving as a common precursor in natural samples. Additionally, various other precursor phases likely exist in both marine and terrestrial environments, including different P-enriched Fe-oxide minerals formed at the sediment-water interface, within burrowing holes, or as Fe plaque along aquatic plant roots.^54,55^ Identifying these precursor phases and localizing microenvironments could facilitate in identifying and quantifying vivianite in situ.

While the formed vivianite in reacted FhP(+Viv) samples at HSF and HW was a major P pool (19–72%), it only constituted a minor fraction of the total Fe pool (7–15%). Detecting this pool size would have been challenging using XRD or even Fe-specific methods such as Fe K-edge XAS. Our approach, combining ^57^Fe-labeled ferrihydrite with ^57^Fe-Mössbauer spectroscopy, enabled the detection of an otherwise easily missed vivianite pool. Consequently, we hypothesize that vivianite may be an easily overlooked mineral affecting P cycling over short and extended timeframes in various coastal sediments. Given the significant role of coastal sediments in global oceanic P removal,^3,56^ vivianite likely plays a crucial role in oceanic P availability.

### Implications for Vivianite Formation in Coastal Ecosystems and Beyond

Mixing ^57^Fe-labeled ferrihydrite with and without adsorbed P into the sediment of three field sites enabled us to detect in situ vivianite formation. Our data highlights vivianite formation within weeks in situ and the significance of an optimal reactive Fe:P solid-phase ratio in triggering vivianite formation. This finding holds the potential for further constraining vivianite distribution in coastal sediments. While our focus was on coastal sediments, our results may apply to other environments with similar geochemical conditions, such as limnic and riparian sediments, rice paddies, and wetland soils. For instance, our findings could inform water quality improvement strategies in lakes, where inducing vivianite formation has been used to enhance long-term water quality.^19^

The formed vivianite pool (19–72% of total P pool based on different techniques) in the Elbe estuary’s low and medium salinity sites is consistent with estimates from other coastal systems,^6,9,10,28^ suggesting that vivianite is likely a major P pool in coastal systems globally. While P bound in vivianite is typically considered unavailable to organisms, vivianite dissolution can occur under oxidizing or sulfidic conditions.^51,57−59^ Thus, the widespread occurrence of vivianite could lead to a future sedimentary P legacy if geochemical conditions change, for instance, due to erosion of tidal flats,^60^ sea level rise,^9^ changes in riverine discharge,^61^ or changes in ocean circulation.^56^ Changes in sedimentary mixed-valence and reduced Fe–P pools, such as green rust and vivianite, likely impacted P availability throughout Earth’s history,^62^ underscoring the importance of understanding current vivianite dynamics to understand better past and future P biogeochemical cycling. Our study adapted a novel approach for detecting in situ vivianite formation, highlighting vivianite’s role in P cycling in coastal sediments and could be easily modified to study vivianite formation in other environmental systems.

## Supporting Information Available

Information on material and methods as well as further results of porewater conditions, sequential Fe extraction,^57^Fe-Mössbauer spectroscopy, LCF of Fe K-edge EXAFS, and P K-edge XANES analysis (PDF)
