# Supplementary material for: In Situ Vivianite Formation in Intertidal Sediments: Ferrihydrite-Adsorbed P Triggers Vivianite Formation
Source: Environ Sci Technol. 2024 Dec 25;59(1):523–32. doi: 10.1021/acs.est.4c10710 (PMC11741103; doi:10.1021/acs.est.4c10710)
Supplement: Supplementary file 1 — es4c10710_si_001.pdf [file es4c10710_si_001.pdf]

Supporting Information

In-situ vivianite formation in intertidal sediments: Ferrihydrite-adsorbed P triggers vivianite formation

L. Joëlle Kubeneck[1*], Katherine A. Rothwell[1,4], Luiza Notini[1,3], Laurel K. ThomasArrigo[1, 4], Katrin Schulz[1], Giulia Fantappiè[1], Prachi Joshi[5], Thomas Huthwelker[6], and Ruben Kretzschmar[1]

[1]Soil Chemistry Group, Institute of Biogeochemistry and Pollutant Dynamics, CHN, ETH Zürich, 8092, Zürich, Switzerland
[2]School of Earth Sciences, University of Bristol, Bristol BS8 1RJ, UK
[3]Department of Civil, Construction, and Environmental Engineering, University of Delaware, Newark, Delaware, 19716, United States
[4]Environmental Chemistry Group, Institute of Chemistry, University of Neuchâtel, 2000 Neuchâtel, Switzerland
[5]Geomicrobiology, Department of Geosciences, University of Tübingen, 72076 Tübingen, Germany
[6]Paul Scherrer Institut, 5232 Villigen, Switzerland
[*]Corresponding author: luisa.kubeneck@usys.ethz.ch

December 19, 2024

Supporting Information Summary

Number of pages: 80

Number of tables: 32

Number of figures: 34

Contents

S1 Materials and methods - additional information **S2**

S1.1 Mineral synthesis S2

S1.1.1 Ferrihydrite synthesis S2

S1.1.2 Vivianite synthesis S3

S1.1.3 Mineral characterization S3

S1.2 Treatment preparation S4

S1.3 Field site and experimental set-up S4

S1.4 Porewater characterization S6

S1.4.1 Temporal monitoring S6

S1.4.2 Porewater depth profiles S6

S1.4.3 Porewater analysis S7

S1.4.4 Calculations of saturation indices S8

S1.5 Analysis of solid phase S8

S1.5.1 Sequential Fe extraction S8

S1.5.2 ^{57}Fe-Mössbauer spectroscopy S8

S1.5.3 Fe K-edge XAS S9

S1.5.4 P K-edge XANES S10

S2 Mineral characterization **S11**

S3 Porewater geochemistry **S12**

S3.1 Porewater depth profiles S20

S4 Geochemistry of reacted solid phase **S26**

S4.1 Sequential Fe extraction S26

S4.2 Mössbauer data - reacted samples S30

S4.2.1 Fitted spectra and hyperfine parameters of reacted samples S30

S4.2.2 Overlay of reacted samples with vivianite and siderite references S45

S4.2.3 Identifying the presence of siderite based on Fe(II) temperature trends . . . S47

S4.2.4 Mössbauer data of initial and reacted unamended sediment S48

S4.3 PCA analysis for LCF of Fe K-edge EXAFS specra S53

S4.4 LCF of Fe K-edge EXAFS spectra S54

S4.5 LCF of Fe K-edge XANES spectra S58

S4.6 Ferrous fraction in solid phase S60

S4.7 P K-edge XAS S62

S1 Materials and methods - additional information

S1.1 Mineral synthesis

Three treatments were tested at the three field sites to investigate factors potentially controlling vivianite formation. Three mineral phases were prepared for these treatments: ^{57}Fe-ferrihydrite, ^{57}Fe-ferrihydrite with adsorbed phosphate, and vivianite. All glassware used for mineral synthesis and phosphate adsorption was acid-washed (10 % hydrochloric acid (HCl), v/v) for 24 h and then rinsed thoroughly with ultra-pure water (UPW, Milli-Q, Millipore,>18.2 MΩ·cm) before use. All solutions were prepared using analytical-grade or higher-quality chemicals.

S1.1.1 Ferrihydrite synthesis

For the synthesis of ^{57}Fe-labeled ferrihydrite (^{57}Fe-Fh), 1 g of ^{57}Fe(0) metal powder (Isoflex, 96.14% ^{57}Fe isotope purity) was dissolved in 100 mL of 2 M HCl (Suprapur, VWR) overnight at room temperature. Subsequently, the solution was oxidized with excess H_2O_2 (35 %, Merck) for an hour and then filtered (0.22 µm, nylon). The pH was raised to 7.5 ± 0.5 by the dropwise addition of 1 M NaOH under constant stirring. The formed precipitates were repeatedly washed in UPW, centrifuged (3800 g for 15 min, 20°C), and the supernatants were decanted until the conductivity of the supernatant was below 100 µS cm^{-1}. The washed precipitates were then resuspended in 300 mL UPW.

The washed homogeneous ferrihydrite suspension was split into two batches for phosphate adsorption. Batch 1 contained 100 mL of the suspension, and batch 2 contained 200 mL. Batch 2 was used to adsorb phosphate to the ferrihydrite (^{57}Fe-FhP). To this end, 26.6 mL of a 0.06 M Na_2HPO_4 solution was added to batch 2 to aim for a final Fe:P molar ratio of 6.7. The pH of both batches was re-adjusted to pH 6.5 ± 0.2 with dropwise addition of NaOH or HCl. Both batches were subsequently shaken (170 rpm) for the whole reaction time (24 h). During the reaction time, the pH was regularly checked and, if needed, re-adjusted. After 24 h, the two batches were centrifuged at 3500 rpm for 15 minutes at 20°C. The precipitates were washed with UPW multiple times until the supernatants' conductivity was below 100 µS cm^{-1}. The washed precipitates were then resuspended in 50 mL UPW (Batch 1) and 100 mL UPW (Batch 2) and shock-frozen by dropwise injection into liquid N_2, freeze-dried, gently homogenized with an agate mortar and pestle, and stored in brown glass bottles in a desiccator until use.

S1.1.2 Vivianite synthesis

Vivianite was synthesized in an anoxic chamber (MBraun, UNIlab Plus, N_2 atmosphere, below 1 ppm (v/v) O_2) and in aluminum-covered amber glass flasks at room temperature. All solutions were prepared with de-oxygenated and CO_2-free UPW. To synthesize vivianite, 900 mL of a 25 mM $FeSO_4$ stock solution with natural abundance Fe isotopes (isotope composition: 5.8% ^{54}Fe, 91.7% ^{56}Fe, 2.2% ^{57}Fe, and 0.3% ^{58}Fe[33]) was prepared and continuously stirred (300 rpm), while 100 mL of a 391 mM Na_2HPO_4 stock solution was added. After mixing the solutions, the pH was 7 and remained stable for the remaining reaction time (pH: 7 $\pm$ 0.1). Upon addition of the Na_2HPO_4 stock solution, a white precipitate appeared immediately, and the suspension was continuously stirred at 300 rpm for 24 h.

After 24 h, the suspension was filtered (0.45 µm nylon) to recover the vivianite (4 g) and was washed with 1 L UPW and dried under dark and anoxic conditions. The dried vivianite was gently homogenized and stored in air-tight amber vials in the anoxic chamber until further usage.

S1.1.3 Mineral characterization

To characterize the elemental contents of the ^{57}Fe-Fh, ^{57}Fe-FhP, and vivianite, ~10 mg of each mineral phase was dissolved (in duplicate) in 10 mL of 1 M HCl (Suprapur, VWR). The solution was subsequently analyzed by inductively coupled plasma optical emission spectroscopy (ICP-OES, Agilent 5100) for Fe and P. Additionally, powder X-ray diffraction (XRD) patterns of the mineral phases were collected with a Bruker D8 ADVANCE diffractometer equipped with a high-resolution energy-dispersive one-dimensional (1-D) detector (LYNXEYE) and Cu anode source ($k_{\alpha 1}$=1.5406 Å; $k_{\alpha 2}$=1.54439 Å). For these analyses, a small aliquot of the dried mineral powder was re-suspended in ethanol and deposited onto a polished silicon wafer (Sil'tronix Silicon Technologies, France), leading to an evenly dispersed crystal deposit. This process was carried out in a dark anoxic chamber for vivianite to avoid oxidation. A Plexiglas dome equipped with an anti-scatter knife edge (Bruker, A100B140) was used to maintain anoxic conditions for vivianite during XRD analysis. For vivianite, the diffractograms were collected in the dark in Bragg-Brentano geometry in the 5° to 70° 2Θ range with a 0.02° step size and an acquisition time of 2 s per step. The acquisition time was increased to 10 s per step for the two ferrihydrite mineral phases. XRD patterns revealed that ^{57}Fe-Fh and ^{57}Fe-FhP contained only ferrihydrite (2-line), and for the vivianite batch, vivianite was the only mineral found (Section S2).

S1.2 Treatment preparation

In August 2020, bulk sediment (5 to 20 cm depth) was collected at each field site. All sediments were dried at 30°C, sieved through a 2 mm sieve, gently homogenized with an agate mortar and pestle, and stored in an ambient atmosphere until use. The elemental composition of the three different sediments was measured by X-ray fluorescence spectrometry (XRF; XEPOS, Spectro) using pressed pellets of milled sediment (Table 1).

S1.3 Field site and experimental set-up

A sequence of individual steps involved in preparing the experiment, setting the experiment up in the field and processing reacted samples is shown in Fig. S1. To avoid the oxidation of vivianite in the FhP+Viv samples, a 15 cm long core liner (UWITEC, PVC-corer, 8.6 cm diameter) was pushed for 2 cm into the sediment, and the subsurface volume of the core liner was then vigorously flushed with N_2 for $\sim$ 3 minutes. Sample holders containing FhP+Viv were removed from the air-tight Al-bags and quickly pushed into the sediment under the N_2-atmosphere inside the core liner. Samplers were installed in a circle with a diameter of $\sim$2 m.

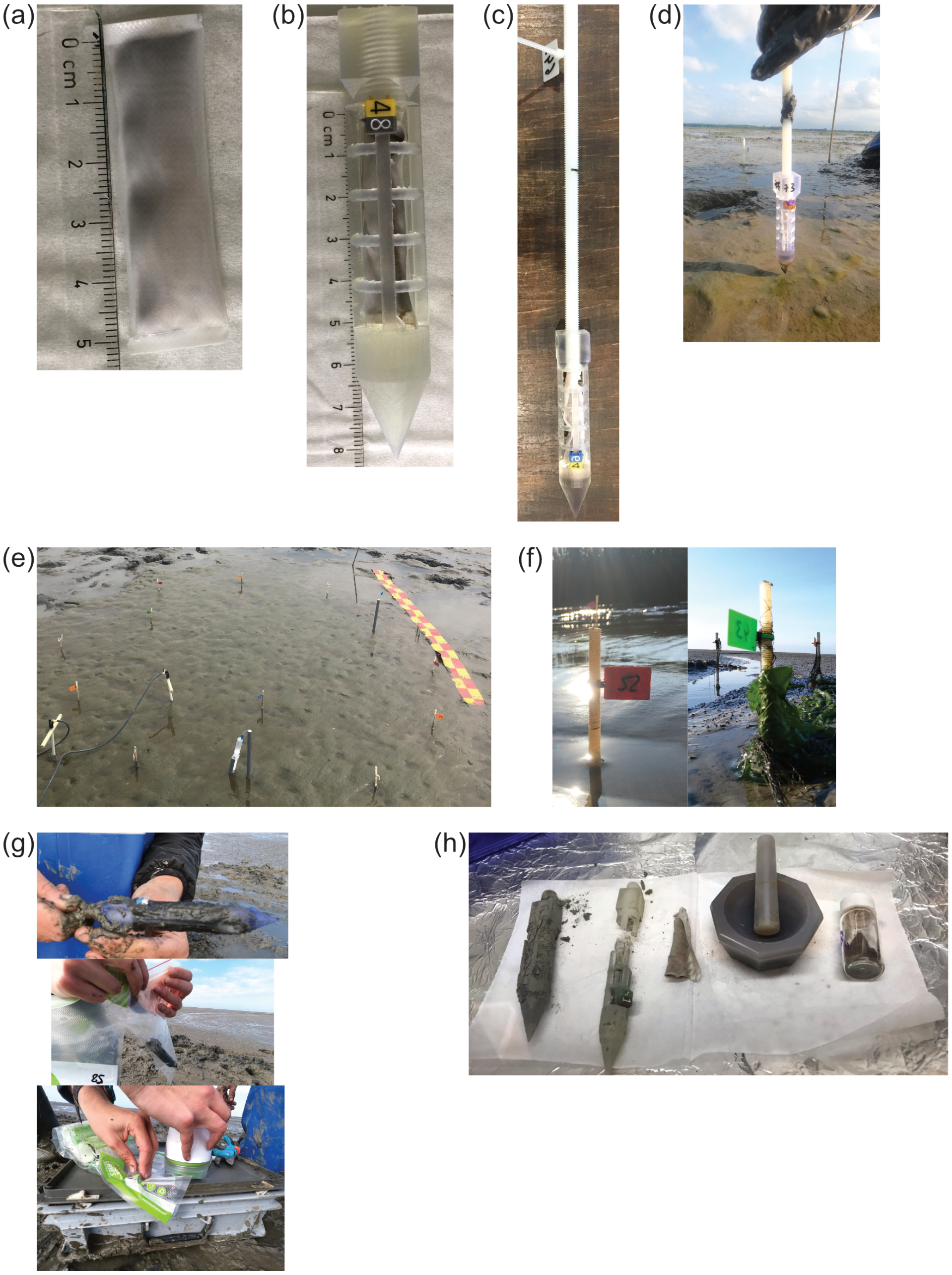

Figure S1 – Overview of the various steps involved in the preparation (a-c), installation(d-f), and processing (g-h) of the field samplers. Preparation of 5 cm long mesh-bags filled with the sediment-mineral mixes (a), which were put into 3-D printed sample holders (b). Sample holders were connected to a labeled nylon rod (c) with which the sample holder was inserted into the sediment at 10 to 15 cm depth (d). At each field site, all three treatments in triplicate were installed in a circle of ∼ 2 m dimension (e). Sample holders were stable during the experimental period (f). Sample holders were at the end of the experiment pulled out of the sediment and directly put into vacuumed bags in the field (g). The frozen, anoxic samples were then air-dried in the glovebox and further processed (h). Pictures a, b, and upper g are also presented in Kubeneck et al.[17]

S1.4 Porewater characterization

S1.4.1 Temporal monitoring

Approximately every 7 to 10 days during the field experiment, sediment temperature (at $\sim$ 10 cm depth) and oxidation-reduction potential (ORP) were measured within the experimental plots during low tide ($\pm$ 3 h). ORP was determined with a custom-made ORP probe with Pt electrodes at 10 and 15 cm depth and an AgCl-reference electrode (supersaturated KCl, Paleo Terra, The Netherlands). Measured ORP values were converted to redox potentials (Eh) relative to the standard hydrogen electrode (+204 mV at 20°C[32]). Reported Eh values should be considered for qualitative comparison between sites since traces of hydrogen sulfide, differences in equilibration time, and slight differences in pH values between sites might affect the reported values. Additionally, porewater samples for major elemental and anion analysis, as well as pH, alkalinity, and hydrogen sulfide, were taken in triplicate from three locations within the experimental plot with MacroRhizons (5 cm long porous part, outer diameter 4.5 mm, 0.15 µm pore size, Rhizosphere, The Netherlands) at a sediment depth of 10 to 15 cm. The first 2 mL of porewater was discarded to clean the MacroRhizons and remove any O_2 from the tubing. Afterward, pre-acidified (30 µl ultra-high purity, concentrated nitric acid), N_2-flushed and pre-vacuumed crimp vials were connected to extract $\sim$3 mL of porewater for elemental analysis. Secondly, N_2-flushed and pre-vacuumed crimp vials containing 2 mL of 2 % zinc acetate solution were connected to extract $\sim$ 2 mL or porewater for hydrogen sulfide analysis. About 5 mL of porewater was collected in a third pre-vacuumed crimp vial. A sub-sample (1 mL) was taken for major anion analysis. In the remaining porewater, pH and total alkalinity were determined.

S1.4.2 Porewater depth profiles

In addition to temporal porewater characterization at the burial depth of the samples, porewater depth profiles were determined for the final time point of the experiment. To this end, six sediment cores per field site of 40 to 50 cm in length were taken by hand using pre-drilled, taped sediment core liners (UWITEC, PVC, 8.6 cm diameter) at the location of the samplers. The retrieved sediment cores were transported back to the field laboratory, where they were processed less than 24 h after core retrieval. For one sediment core, porewater was extracted to determine pH, alkalinity, and major anion content. To this end, microrhizons (19.21.23 Rhizons CSS, 0.15 µm pore size, flat tip, Rhizosphere, The Netherlands) were inserted into the pre-drilled holes from top to bottom. The first few drops of porewater were discarded before connecting a pre-vacuumed, crimp vial to collect $\sim$ 3 mL porewater from each hole with a depth resolution of 2 cm and 5 cm from 0 to 20 cm and 20 to 40 cm depth, respectively. Each porewater sample was used to measure pH, subsequently splitting

the porewater sample for total alkalinity analysis (∼ 2 mL) and major anion analysis (∼ 1 mL). The other 5 sediment cores were used to extract porewater into pre-acidified (30 µl ultra-high purity, concentrated nitric acid), N_2-flushed and pre-vacuumed crimp vials for elemental analysis with a depth resolution of 2 cm and 5 cm from 0 to 20 cm and 20 to 40 cm depth, respectively. Collected porewater samples for elemental analysis from five cores provided insights into the geochemical heterogeneity at the field sites.

After ∼ 5 weeks into the experiment, two ∼40 cm sediment cores (UWITEC, PVC, 8.6 cm diameter) were taken manually at each field site for methane (CH_4) and porosity analysis. CH_4 was sampled immediately after core retrieval from pre-drilled holes in the core liner with a 5 cm depth resolution. 2 cm^3 of wet sediment samples were taken and immediately transferred into a 20 mL serum vial filled with 5 mL saturated NaCl solution. The bottles were capped with stoppers and stored upside down. Once all samples were taken, a 5 mL N_2 gas overpressure was injected into the glass bottles. After that, the samples were stored upside down at room temperature. Within ∼ 5 h, 3 mL of the headspace of the samples was transferred to pre-flushed Exetainer vials. The porosity core was sliced under oxic conditions with a 5 cm depth resolution. Porosity was determined based on the mass difference between wet and dry sediments, assuming a dry sediment density of 2.65 g/cm^3.

S1.4.3 Porewater analysis

Porewater samples for pH and total alkalinity were processed within 8 h after porewater collection. Total alkalinity was determined via a two-step titration (Titrimetric test kit, VISOCOLOR HE Carbonate hardness, Macherey-Nagel). Samples for major anion analysis (Cl^-, Br^-, F^-, and SO_4^{2-}) were frozen at -20°C until analysis with ion chromatography (IC, Metrohm 040 Professional IC Vario). Samples for hydrogen sulfide species were preserved by adding zinc acetate and cooled (4°C) until spectrophotometrical measurements.[6] Samples for elemental analysis (Ca, Mg, Fe, Mn, K, Na, Si, P, and S) were acidified and cooled (4°C) until analysis by ICP-OES. CH_4 samples were analyzed on a TraceGC1300 (ThermoFisher Scientific, modified by S+HA analytics) gas chromatograph, in which the sample is split into two different column configurations (first configuration: 30 m long, 0.53 mm ID TGBondQ column and 30 m long, 0.53 mm ID Molsieve column; second configuration: 30 m long, 0.53 mm ID TGBondQ column and a 30 m long 0.25 mm ID TGBondQ+ column (all ThermoFisher Scientific)), each connected to a Pulse Discharged Detectors. Gas concentrations were quantified with external calibration.

S1.4.4 Calculations of saturation indices

Porewater species concentrations (pH, alkalinity, and elemental and major anion concentrations) were used to calculate supersaturation indices (SI) of possible solids with VisualMINTEQ (Version 3.1) using the default database (thermo.vdb, comp_2008.vdb) and for vivianite the updated equilibrium constant of Brady et al.[3] The equilibrium constants were corrected for sediment temperature, and ionic strength was calculated using the Davies equation for ion activity corrections. The SI values reported in this paper were based on calculations in which oversaturated solids were not allowed to precipitate.

S1.5 Analysis of solid phase

S1.5.1 Sequential Fe extraction

Sub-samples of the homogenized, dried initial and reacted solid-phase ($\sim$ 50 mg) were subjected to a six-step sequential Fe extraction following a combination of Poulton & Canfield[26] and Claff et al.[5] to gain information about the reactivity of the sedimentary Fe pools. Solid-phase Fe was fractionated step-wise into the following phases: (1) exchangeable Fe extracted by 1 M $CaCl_2$, (2) highly reactive Fe, including Fe carbonates (siderite), vivianite, green rust, and likely the majority of mackinawite and FeS_x,[41] extractable by 1 M Na-acetate, (3) easily reducible Fe (oxyhydr)oxides extracted by 1 M hydroxylamine-HCl solution in 25% v/v acetic acid, (4) reducible Fe (oxyhydr)oxides extracted by sodium dithionite solution buffered to pH 4.8 with 0.35 M acetic acid/0.2 sodium citrate, (5) Fe in recalcitrant oxides extracted by 0.2 M ammonium oxalate/0.17 M oxalic acid (pH 3.2), and (6) Fe associated to pyrite extracted by concentrated HNO_3. Steps 1 to 3 were carried out inside an anoxic chamber and steps 4 and 5 under N_2 flow to avoid oxidation artifacts. All filtrates of each step (0.45 µm, nylon or PTFE) were analyzed for dissolved Fe by ICP-OES, correcting for matrices effects. The sum of the first five steps is classified as reactive Fe as this fraction will, over geological time scales, react with sulfides to form iron sulfide minerals.[4]

S1.5.2 ^{57}Fe-Mössbauer spectroscopy

^{57}Fe-Mössbauer spectra were collected using a ^{57}Co/Rh γ-radiation source in constant acceleration mode in transmission setup (WissEl, Wissenschaftliche Elektronik GmbH) for dried initial and reacted samples. Sub-samples of the triplicates of each treatment and field site were mixed into one homogenized sample ($\sim$ 100-150 mg) inside an anoxic chamber. Samples were then sealed between two pieces of Kapton tape in the anoxic chamber until immediately before analysis. The samples were measured at 77, 25, 13, 10, and 5 K inside a closed-cycle exchange gas (He) cryostat (SHI-850, Janis Research Co.). A temperature profile with five temperatures was chosen to aid mineral

identification based on different Néel temperatures. Spectra were calibrated with an α-Fe(0) foil at 295 K, and the line width was set according to the inner broadening of the calibration foil at 295 K. The collected spectra were normalized to 1 with respect to absorption. Fitting was done using Recoil software (University of Ottawa, Canada) using an extended Voigt-Based fitting (xVBF) routine[28,20] or Full Static Hamiltonian (FSH) fitting routine[1] as presented for similar samples by Notini et al.[23]

S1.5.3 Fe K-edge XAS

To gain further insights into the bulk Fe geochemistry of the solid phase Fe K-edge X-ray absorption spectroscopy (XAS) was used. For the analysis, triplicate samples were combined and milled by hand to a particle size < 100 µm in an anoxic chamber. The homogenized powder samples were pressed into 10 mm pellets, sealed between Kapton tape, and packed for transport to the synchrotron facility in double-sealed, airtight Al-bags in the anoxic chamber.

X-ray absorption near edge structure (XANES) and extended X-ray absorption fine structure (EXAFS) spectra on the Fe K-edge (7112 eV) for reacted Fh, FhP and FhP+Viv samples of all field sites were collected at $\sim$ 20 K using a He(l) cryostat at the SAMBA beamline of SOLEIL (Saint-Aubin, France). For samples of field sites HSF and FKS, Fe K-edge XANES and EXAFS spectra (up to 25 scans per sample) were collected in fluorescence mode using a 36-element array Ge detector (Canberra). Samples of field site HW were measured in transmission mode (5 scans per sample). The monochromator (Si(220)) was calibrated to the first derivative maximum of the K-edge absorption spectrum of a metallic Fe-foil (7112 eV).

Fe K-edge (7112 eV) XANES and EXAFS spectra of the initial sediment and reacted sediment (Control) of all field sites were collected at the Balder beamline of the MAXIV synchrotron (Lund, Sweden). Spectra were recorded in either fluorescence or transmission mode, depending on the Fe content, at $\sim$ 20 K using a He(l) cryostat. Fluorescence spectra were collected using a 7-element SSD and Ge detector. The monochromator (Si(111)) was calibrated to the first-derivative maximum of the K-edge absorption spectrum of a metallic Fe-foil (7112 eV). Higher harmonics in the beam were eliminated by combining Si mirrors and detuning the monochromator by 50 % of its maximal intensity. Four to ten scans per sample were collected and averaged.

All spectra were energy calibrated, pre-edge subtracted, and post-edge normalized in Athena[29] with the edge energy, E_0, defined as the maximum peak in the first XANES derivative. Linear combination fit (LCF) analyses of k^3-weighted Fe K-edge XANES spectra were conducted over an energy range of -20 to 30 eV (E-E_0) to determine the Fe(II) fraction. Fe(II) was modeled by reduced smectite and Fe(III) was modeled by ferrihydrite.

In order to evaluate a suitable number of references used for LCF of Fe K-edge EXAFS spectra,

we employed principal component analysis (PCA) using SixPack (further information in Supplementary material S4.3).[43] Following PCA, target-transformation testing (TT, 4 components) was undertaken in SixPack. The number of suitable references obtained by TT exceeded the number of PCA components. All suitable references, selected based on geochemical considerations and ^{57}Fe-Mössbauer fits, were initially considered in the LCF analysis. The combinatorics LCF was limited to the number of references determined by PCA to reduce the total number of references used. LCF analysis of k^3-weighted Fe EXAFS spectra was performed over a k-range of 2 to 10.5 or 11.5 $Å^{-1}$, depending on the field site with the E_0 of all spectra and reference compounds set to 7128 eV (Fe). No constraints were imposed on any of the LCF fits, and initial fit fractions were recalculated to a compound sum of 100 %. In the final fit, four-component fits were compared to three-component fits and only accepted when the normalized sum of squared residuals value decreased by at least 10%[14] and fitted fractions had to contribute at least 5% to be accepted in the fit. The detection limit of a phase in Fe K-edge EXAFS linear combination fit analysis can be as high as 10-20% and depends on the fitted mineral (presence of distinct spectral features) and signal-to-noise ratio of the spectrum.[14]

S1.5.4 P K-edge XANES

Bulk P K-edge XANES analysis was conducted on selected samples to gain insights into the P mineralogy. Hand-milled samples of FhP (triplicates combined) and Control were mounted on double-sided carbon tape and packed for transport to the synchrotron in double-sealed, airtight Al-bags in the anoxic chamber.

XANES spectra on the P K-edge (2152 eV) were collected for the initial and reacted FhP and for the Control sample under vacuum at the PHOENIX beamline of the Swiss Light Source (PSI, Switzerland). P K-edge XANES spectra were collected in fluorescence mode using a four-element silicon drift Vortex detector at room temperature. Two to four scans were collected per sample and averaged. Monochromator energy was calibrated against the first derivative maximum of $CaPO_4$ at 2152.0 eV.

All P K-edge XANES spectra were analyzed using Athena.[29] The E_0 edge energy was chosen as the maximum of the first derivative of the data. Spectra were normalized using a linear regression fit through the pre-edge region (-18 to -8 eV relative to E_0), and a polynomial regression fit through the post-edge region (30 to 45 eV relative to E_0). LCF analysis was done using weighted combinations of reference spectra from 10 pre-defined groups. Due to the similarity of XANES spectra of different P species (for instance, P adsorbed to ferrihydrite or goethite), 10 groups were pre-defined and fitted using a representative spectrum (Table S22). No energy shifts were permitted in the fitting procedure. At most, five standards were accepted in each fit, and the fitting range was constrained

to between -10 to 30 eV relative to E_0. Only fits with a sum of 90 to 110% were accepted and re-normalized to 100 %. Additionally, fitted fractions had to contribute at least 5% to be accepted in the fit.

S2 Mineral characterization

X-ray diffraction (XRD) confirmed the purity of the synthesized vivianite (Fig. S2). Rietveld analysis was conducted as described by Kubeneck et al.[18] Table S1 shows the results of the elemental analysis of the synthesized initial minerals following acid digestion and subsequent analysis by inductively coupled plasma optical emission spectroscopy (ICP-OES, Agilent 500). The elemental ratios were used to calculate the mineral additions for each treatment and how mineral addition resulted in changes in elemental ratios in the prepared samples. The ^{57}Fe-labeled laboratory synthesized ferrihydrite and ferrihydrite with adsorbed phosphate consisted purely out of 2-line ferrihydrite. The presence of other crystalline Fe-phases was excluded as no diffraction peaks were detectable by XRD (Fig. S3).

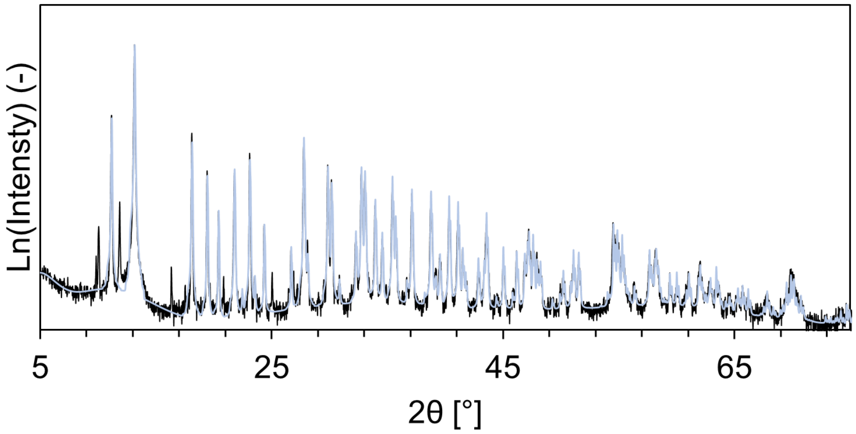

Figure S2 – XRD patterns of vivianite. Data is presented in black, and Rietveld fit for vivianite in light blue. The goodness of fit for the Rietveld fit was 2.22. XRD patterns of the mineral phases were collected with a Bruker D8 ADVANCE diffractometer equipped with a high-resolution energy-dispersive one-dimensional (1-D) detector (LYNXEYE) and Cu anode source ($k_{\alpha1}$=1.5406 Å; $k_{\alpha2}$=1.54439 Å)

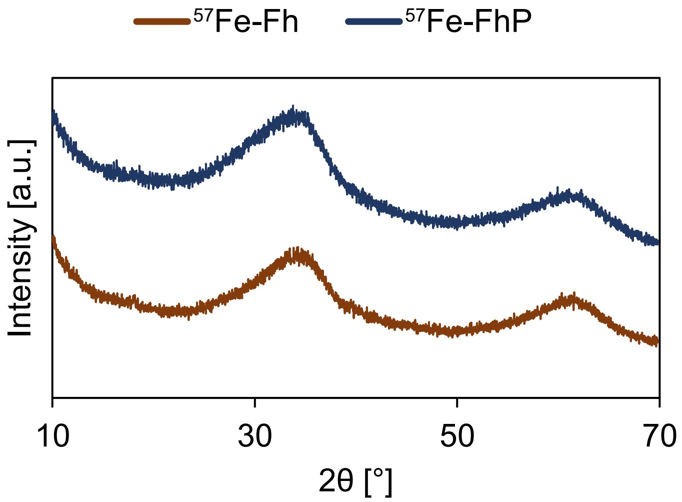

Figure S3 – XRD patterns of ^{57}Fe labeled ferrihydrite (^{57}Fe-Fh) and ferrihydrite with adsorbed phosphate (^{57}Fe-FhP). XRD patterns of the mineral phases were collected with a Bruker D8 ADVANCE diffractometer equipped with a high-resolution energy-dispersive one-dimensional (1-D) detector (LYNXEYE) and Cu anode source ($k_{\alpha 1}$=1.5406 Å; $k_{\alpha 2}$=1.54439 Å).

Table S1 – Fe and P content of the used mineral phases per gram of mineral determined by acid digestion followed by ICP-OES analysis.

**Mineral**	**Fe [wt %]**	**P [wt%]**	**Fe:P [mol:mol]**
^{57}Fe-Fh	57		
^{57}Fe-FhP	52	4	7.14
Vivianite	36	12	1.66

S3 Porewater geochemistry

Sediment temperature at the three field sites was regularly recorded at 10 cm depth and ranged from 13°C to 21°C (Fig. S4). Across all field sites, circumneutral porewater pH values persisted during the experimental period (Fig. S5). The recorded temperature and pH were used in thermodynamic calculations to calculate whether the porewater was oversaturated with respect to vivianite and siderite at 10-15 cm depth (Fig. S6). At HSF and HW, the porewater was oversaturated with respect to vivianite and siderite during the whole experimental period. At FKS, the porewater was undersaturated with respect to vivianite, while the saturation index (SI) of siderite ranged from -1 to 0 and slightly above, suggesting that the porewater might have been oversaturated with respect to siderite occasionally.

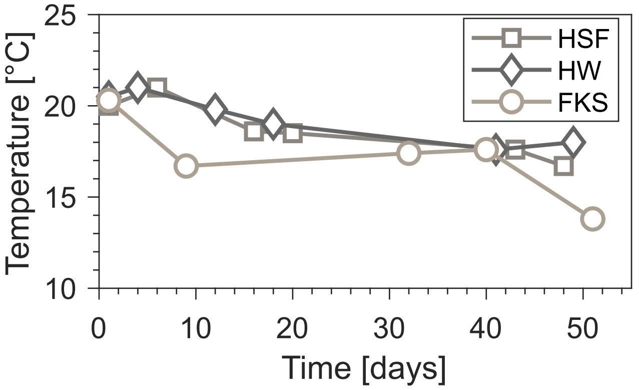

Figure S4 – Sediment temperature at a depth of 10 cm during the field experiment at the three field sites.

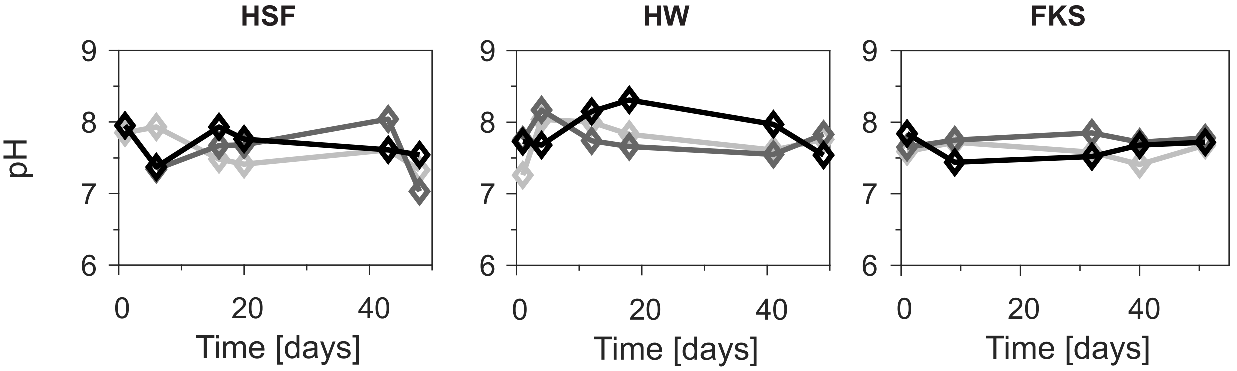

Figure S5 – Porewater pH values recorded during the experimental period. Porewater samples were collected at 12.5 cm depth from three locations within the experimental plot, indicated by different shades of colors in the figure.

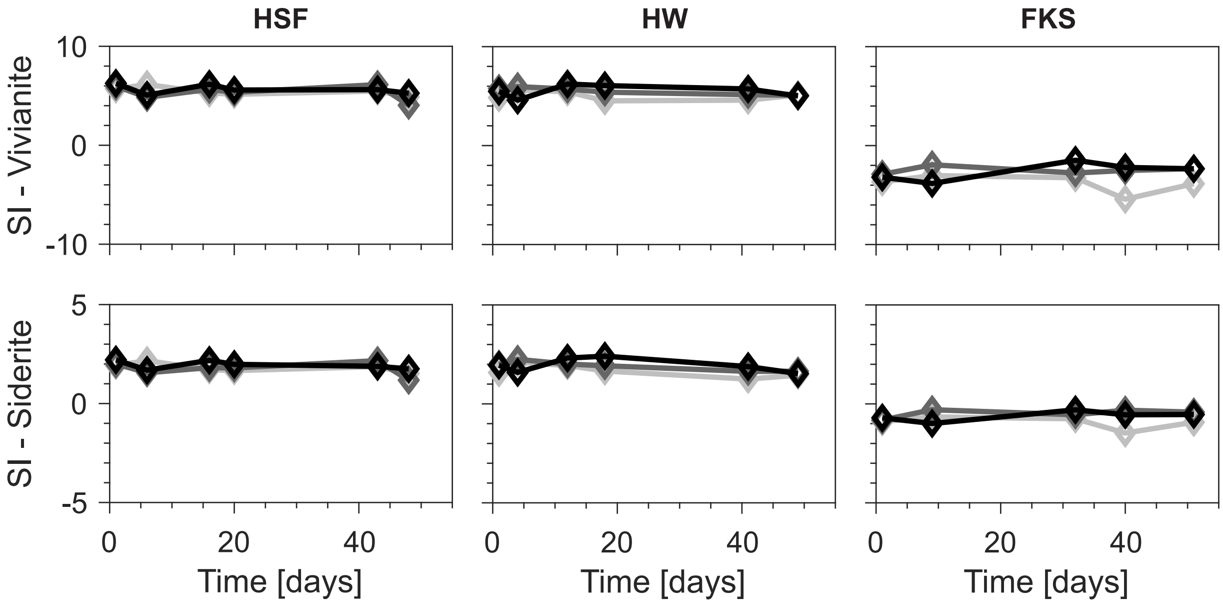

Figure S6 – Temporal trend of supersaturation of the porewater with respect to vivianite and siderite at the three field sites at 12.5 cm sediment depth. The different shades of color indicate the results calculated from the porewater data collected from three locations within the experimental plot.

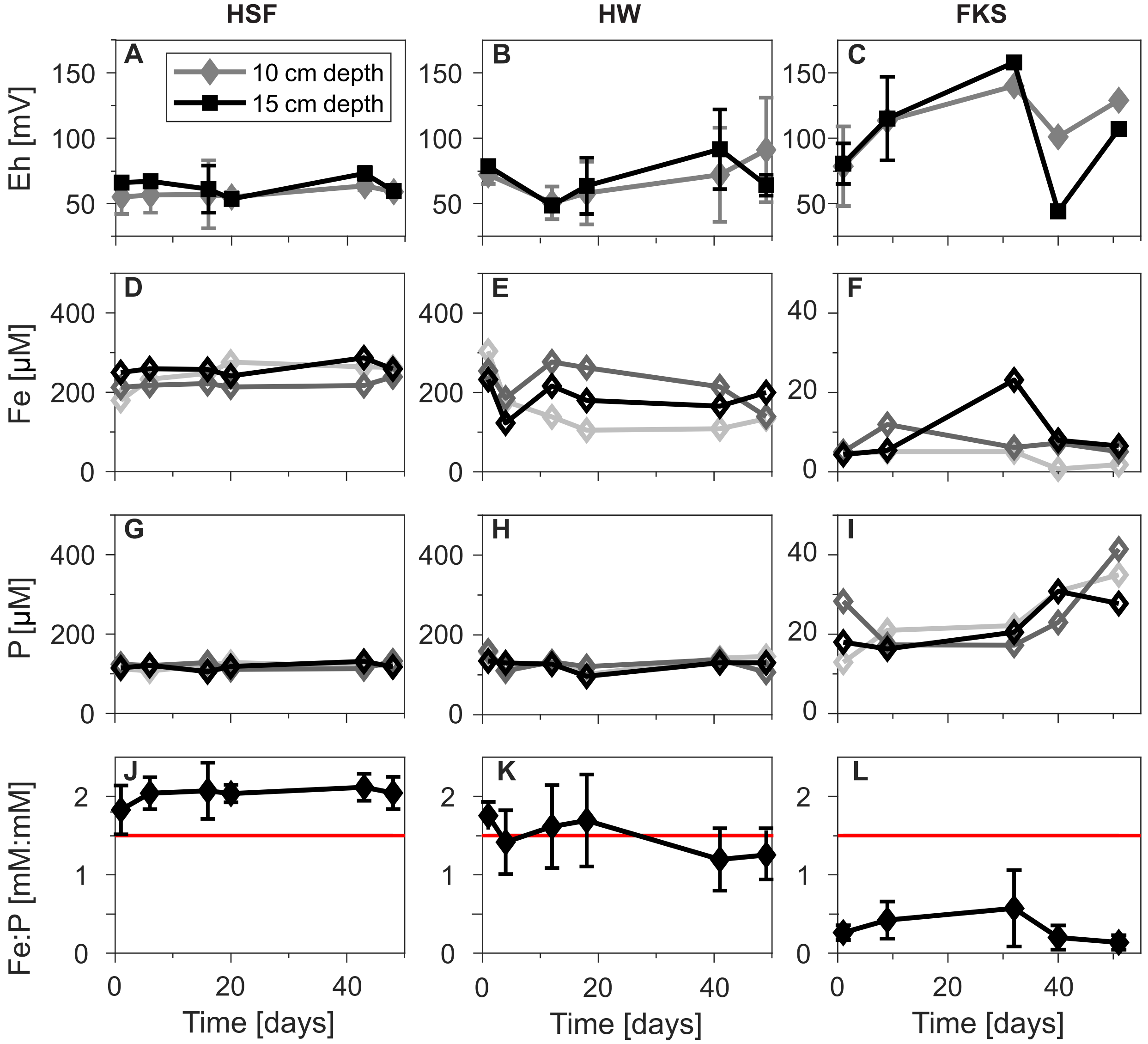

Figure S7 – Temporal trend of Eh, dissolved Fe, dissolved P, and dissolved Fe:P molar ratio at the three field sites during the experiment at 12.5 cm sediment depth. Note the different y-axis dimensions for dissolved Fe and P at FKS. Eh was recorded at two different depths indicated by different symbols and shades of color (ABC). Porewater samples were collected from three locations within the experimental plot, indicated by different shades of colors (D-I). The dissolved Fe:P molar ratio is the average of the three recorded porewater values and error bars show the standard deviation. The red line (JKL) indicates a dissolved Fe:P molar ratio of 1.5, the theoretical stoichiometric ratio of vivianite. Abbreviations: HSF = Haseldorfer Marsch - low salinity site, HW = Hollerwettern - medium salinity site, FKS = Friedrichskoog - high salinity site.

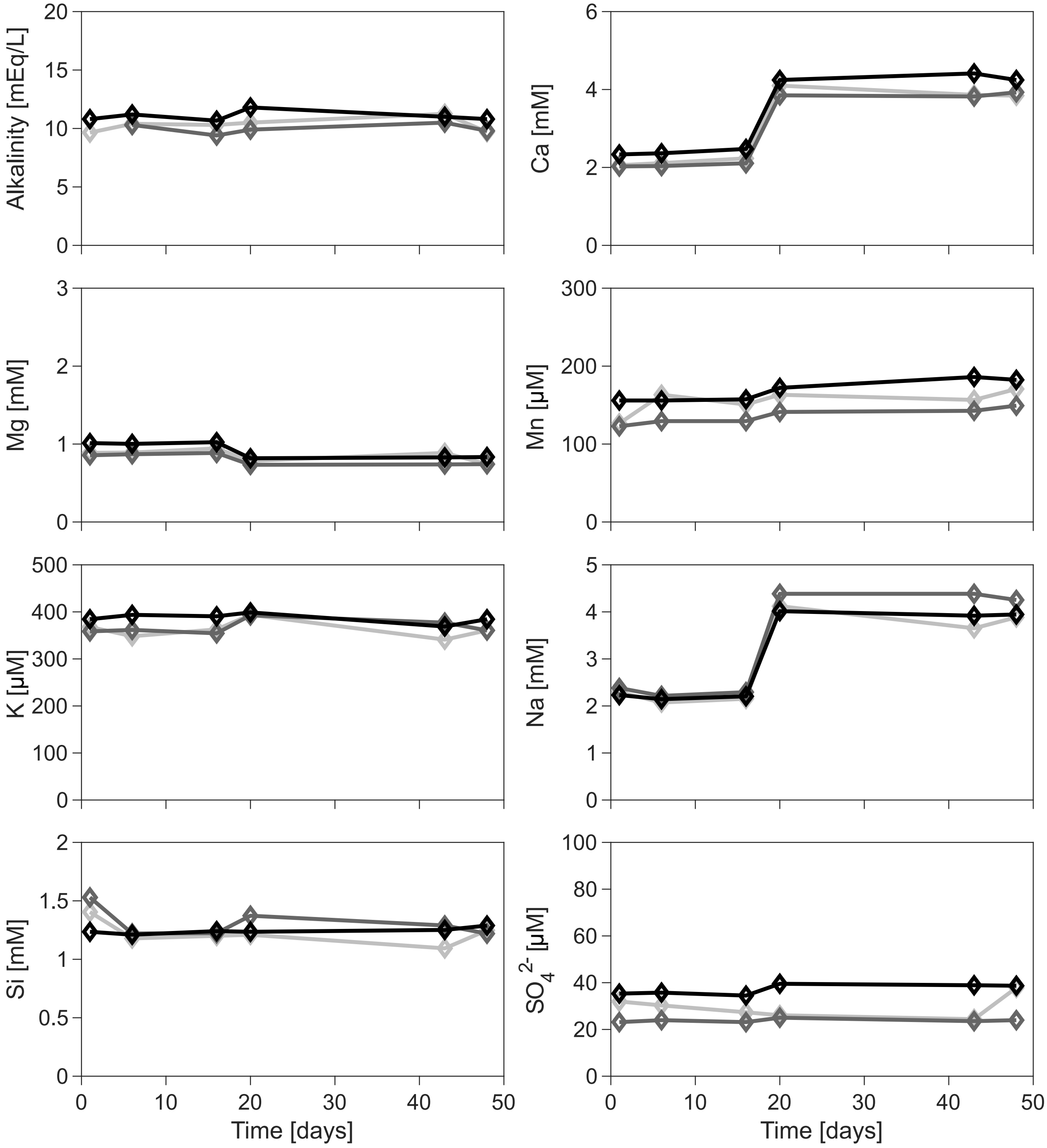

Figure S8 – Additional porewater concentration trends at HSF during the experimental period at 12.5 cm sediment depth. Porewater samples were collected from three locations within the experimental plot, indicated by different shades of colors.

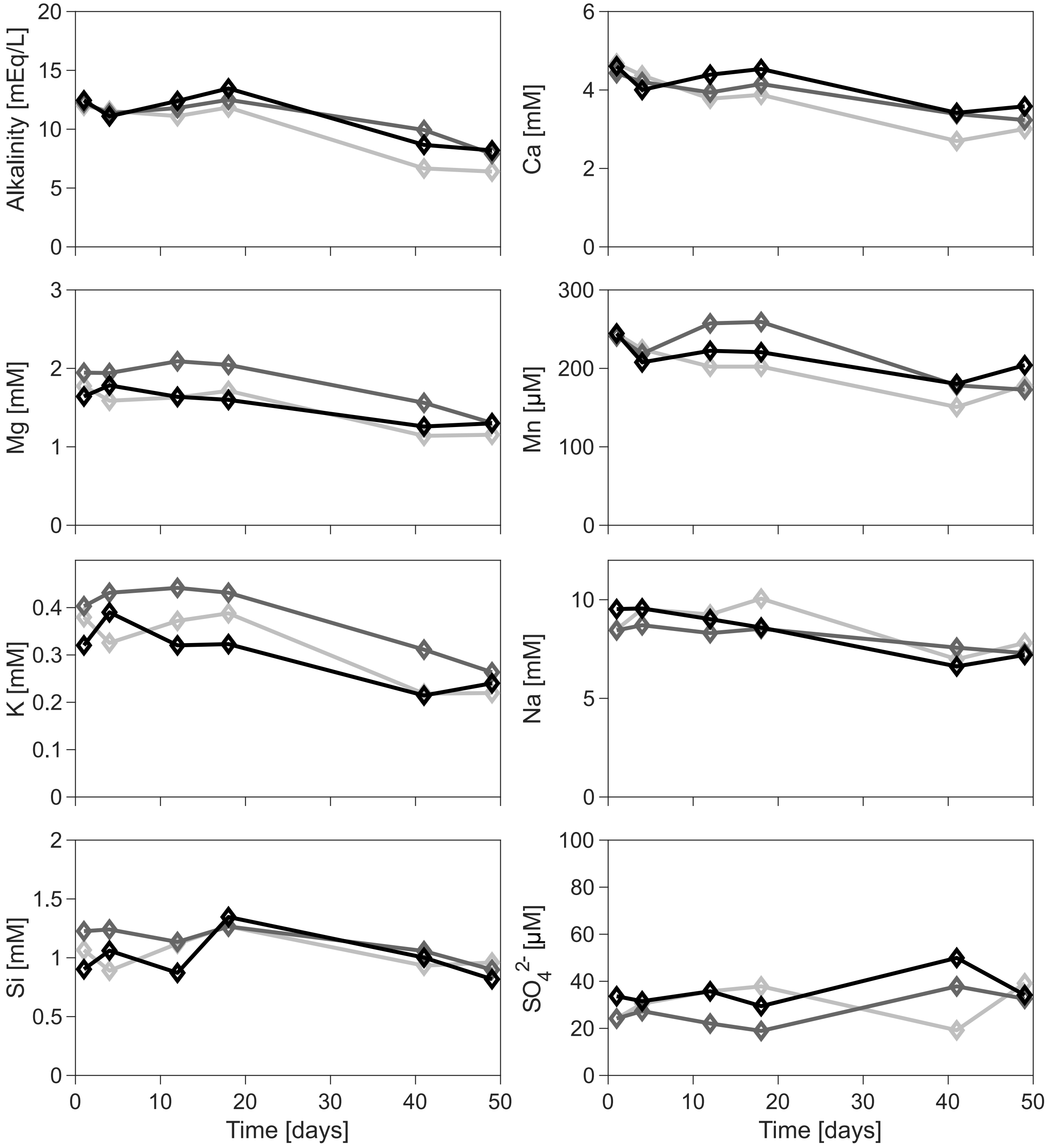

Figure S9 – Additional porewater concentration trends at HW during the experimental period at 12.5 cm sediment depth. Porewater samples were collected from three locations within the experimental plot, indicated by different shades of colors.

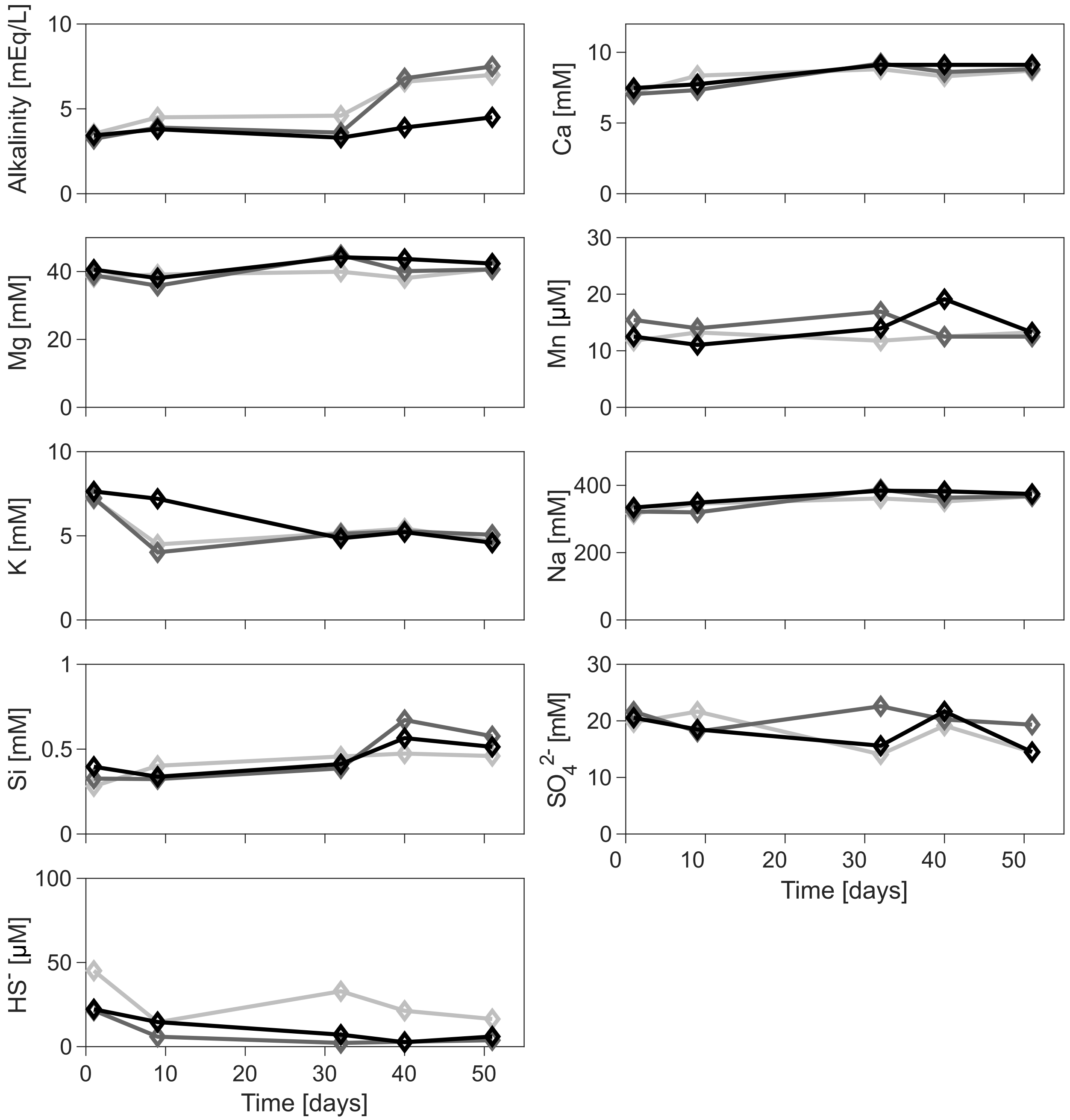

Figure S10 – Additional porewater concentration trends at FKS during the experimental period at 12.5 cm sediment depth. Porewater samples were collected from three locations within the experimental plot, indicated by different shades of colors.

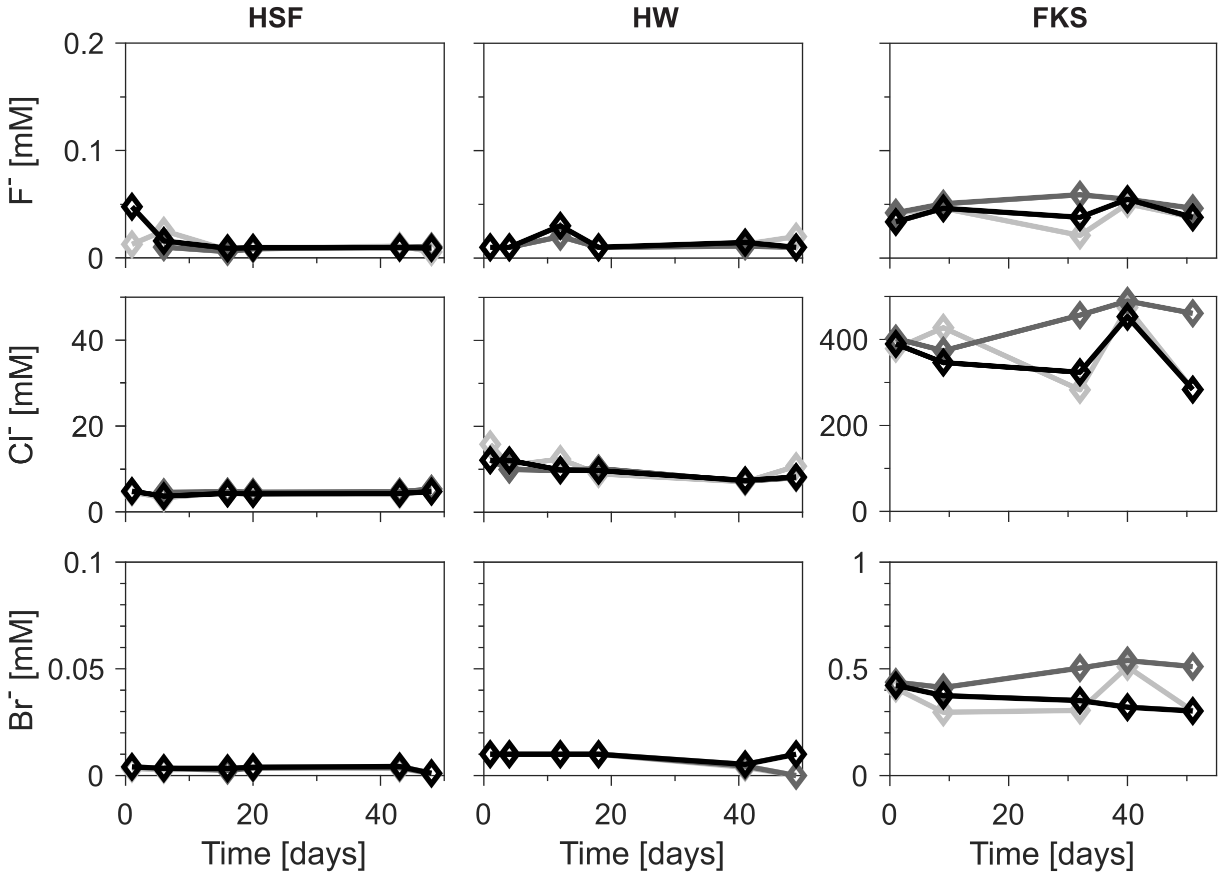

Figure S11 – Temporal trend of F^-, Cl^- and Br^- at the three field sites during the experiment at 12.5 cm sediment depth. Porewater samples were retrieved from three locations within the experimental plot, indicated by different shades of colors in the figure.

S3.1 Porewater depth profiles

To characterize in detail the depth-resolved porewater geochemistry of each site, multiple sediment cores were taken and analyzed. Figure S12 provides a direct comparison between the depth-resolved data, while Figures S13 to S15 show the porewater data individually for each field site. The collected porewater data was subsequently used for thermodynamic calculations to calculate the SI of the porewater with respect to vivianite and siderite with depth (Fig. S16).

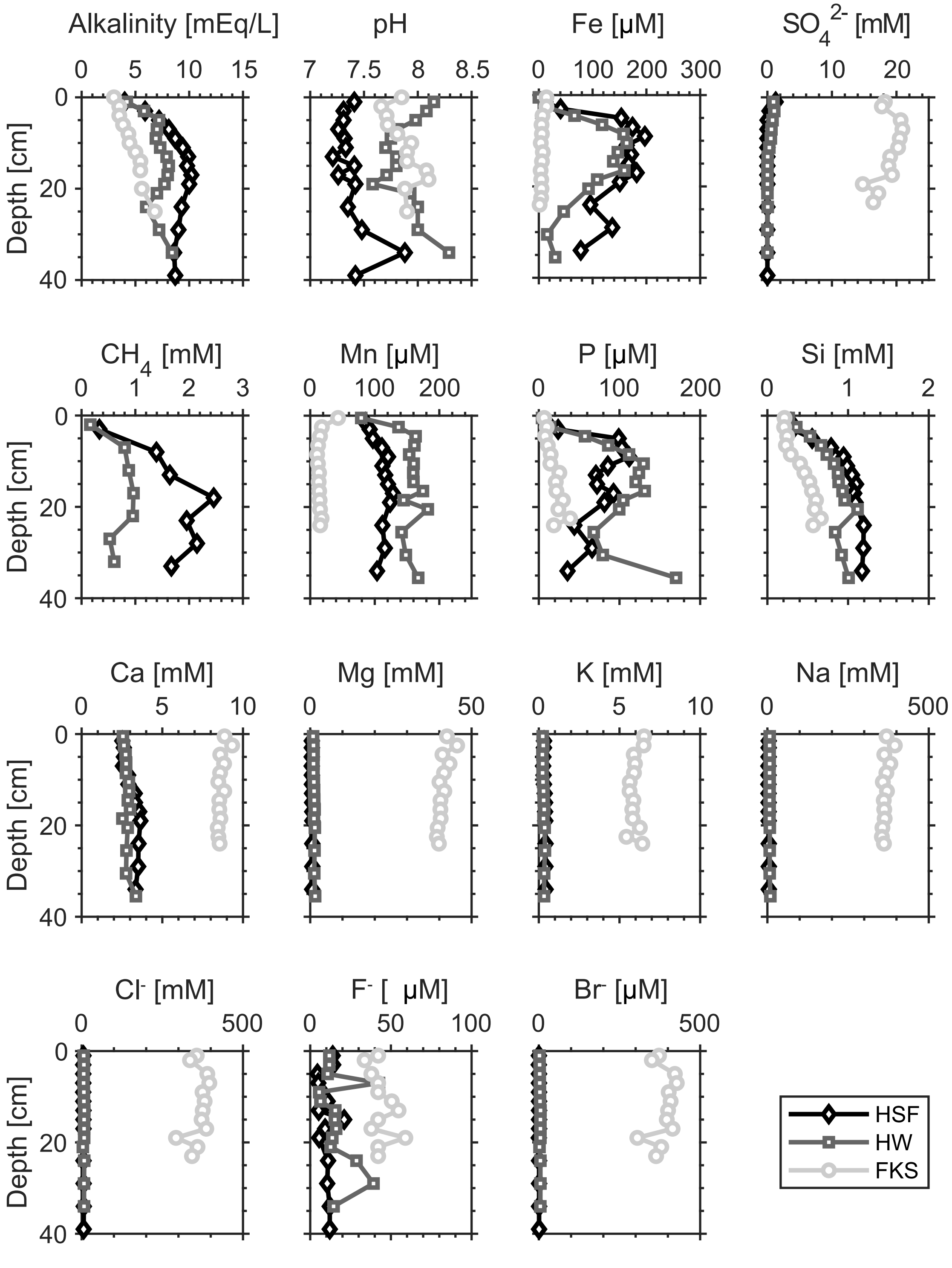

Figure S12 – Sediment porewater depth profiles at HSF, HW, and FKS. Rescaled depth profiles for each field site are presented in Fig. S13, S14, S15. No CH_4 was detected at FKS.

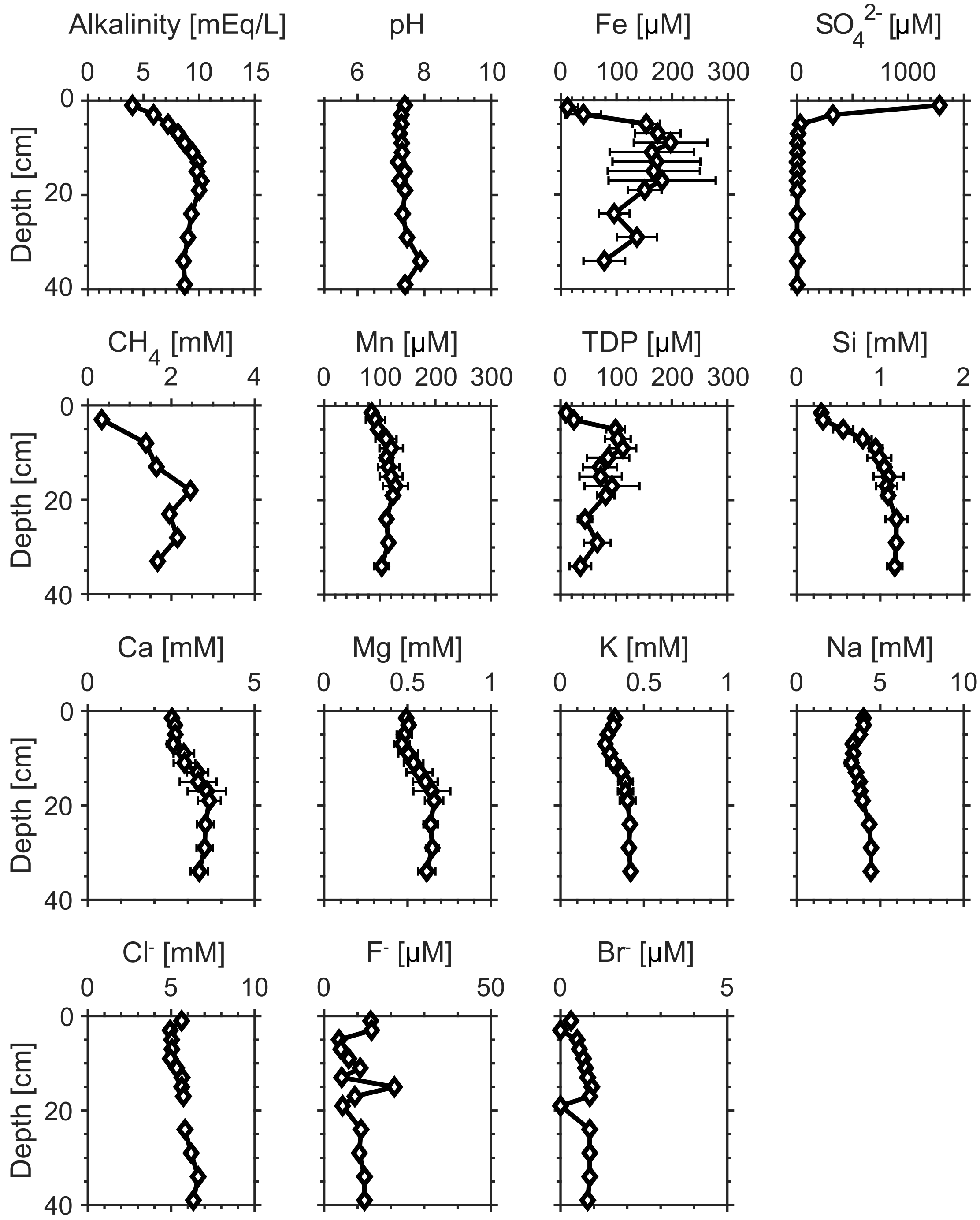

Figure S13 – Sediment porewater depth profiles at HSF. Error bars for Fe, Mn, P, Si, Ca, Mg, K and Na indicate the standard deviation derived from the average of five sediment cores.

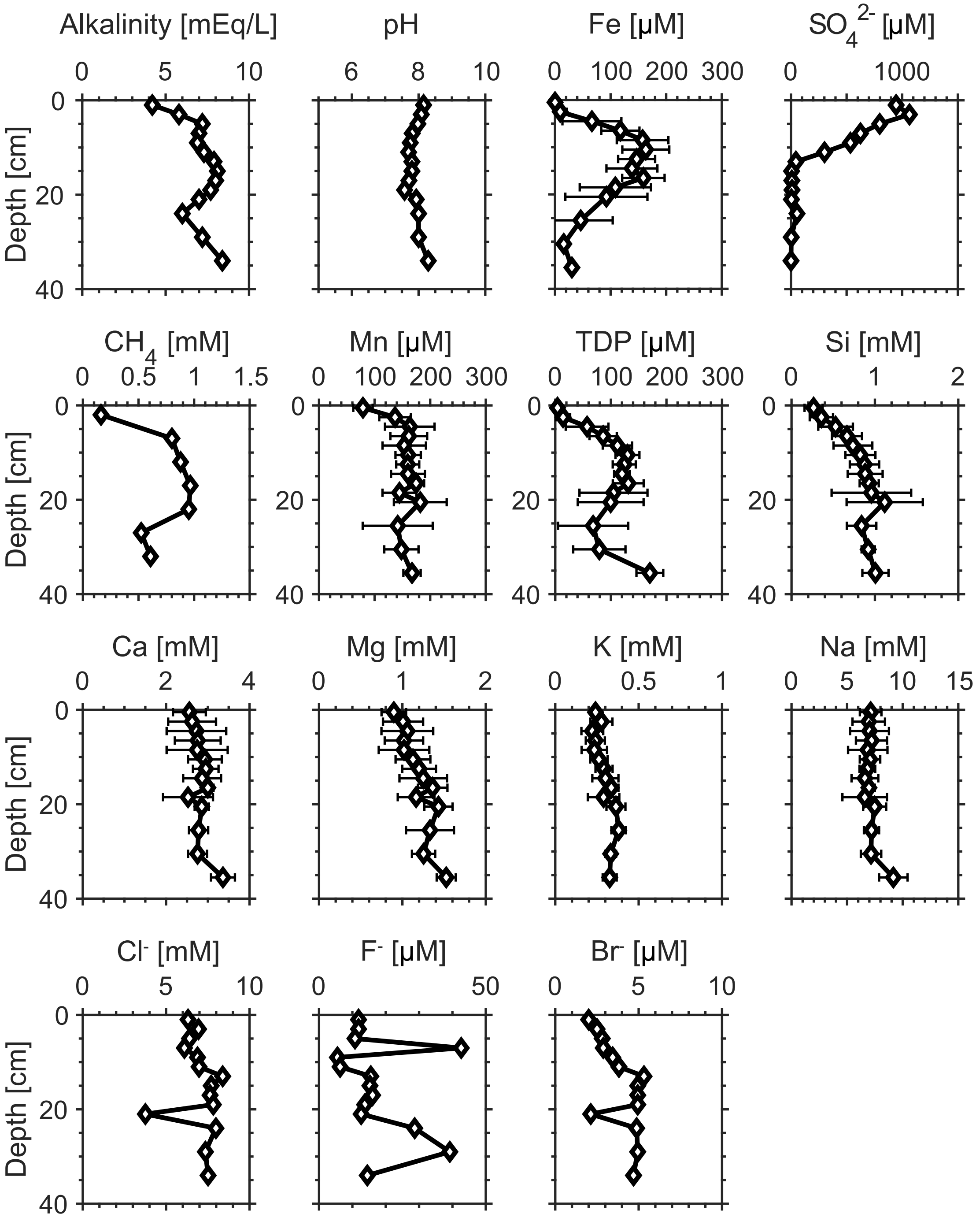

Figure S14 – Sediment porewater depth profiles at HW. Error bars for Fe, Mn, P, Si, Ca, Mg, K and Na indicate the standard deviation derived from the average of five sediment cores.

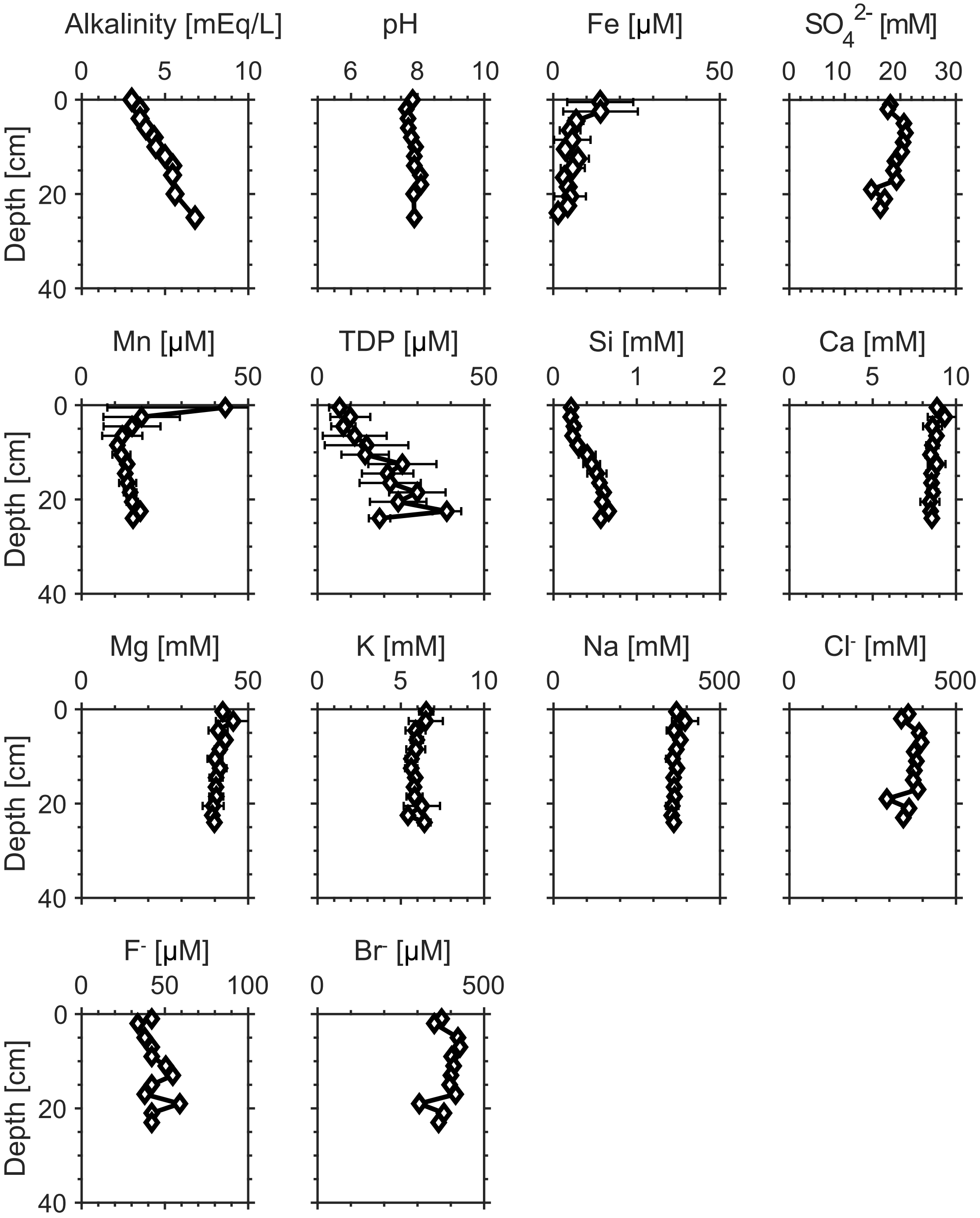

Figure S15 – Sediment porewater depth profiles at FKS. Error bars for Fe, Mn, P, Si, Ca, Mg, K and Na indicate the standard deviation derived from the average of five sediment cores.

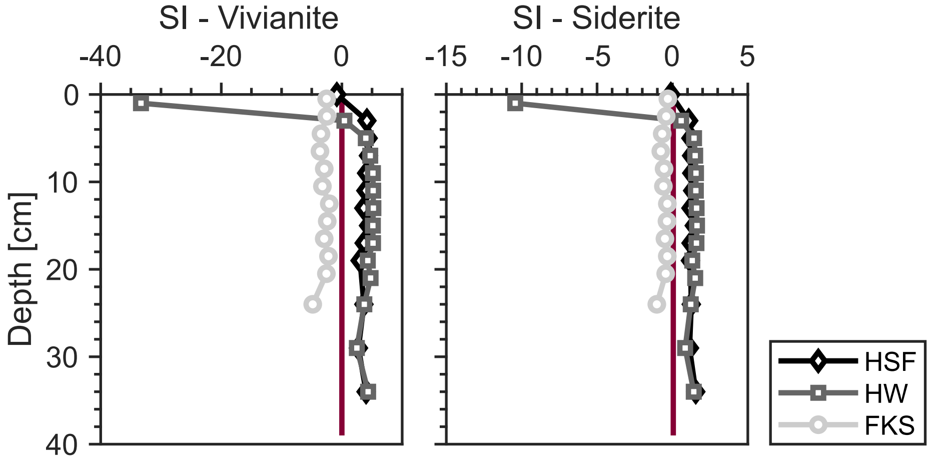

Figure S16 – Change in porewater supersaturation with respect to vivianite (left) and siderite (right) with depth at the three field sites. Porewater data used to perform thermdynamic equilibrium calculations were collected at the end of the experiment. The dark red line indicates the zero-crossing of the x-axis. Thus, data points to the right of the red line indicate the supersaturation of the porewater with respect to vivianite or siderite, respectively. Abbreviation: SI = Saturation index.

S4 Geochemistry of reacted solid phase

S4.1 Sequential Fe extraction

A six-step sequential Fe extraction was performed to gain knowledge about the reactivity of the bulk Fe mineralogy of the unamended sediment, initial, and reacted samples. The sum of the first five steps is defined as reactive Fe as this fraction will, over geological time scales, react with sulfides to form thermodynamically stable Fe-sulfide minerals.[4]

Across all field sites, the sequential extraction yielded $\sim$ 30 to 65% of total Fe content, indicating that the remaining Fe was present as silicate minerals,[26] which is coherent with LCF results of Fe K-edge EXAFS analysis (Section S4.4). Initial samples of each treatment (Fh, FhP, and FhP+Viv) showed increases in hydroxylamine-extractable Fe (Step 3, Fig. S17, Table S2) in comparison to unamended sediments, which targets poorly crystalline ferric minerals and thus dissolved the added ferrihydrite. Furthermore, a minor increase in Na acetate-extractable Fe (Step 2, Fig. S17, Table S2) was observed, which targets highly reactive Fe, including siderite, vivianite, and green rust (in-house tests).[26] After incubation, most Fe was extracted ($\sim$ 40 to 50 %) by Na acetate (Fig. S17, Table S3), accompanied by a decrease in hydroxylamine-extractable Fe for all reacted samples. This switch suggests the formation of highly reactive Fe phases, such as green rust and vivianite, following the reductive dissolution of ferrihydrite. Moreover, there were increases in the CDB-extractable Fe (Step 4; $\sim$ 11 to 19%, Fig. S17, Table S3), targeting more crystalline ferric minerals[26] and thus indicating the formation of more crystalline Fe-oxide minerals such as lepidocrocite or goethite.

Table S2 – Results of sequential Fe extraction for initial sediment and treatments for each field site. Reactive Fe is defined as the sum of the first five steps of the sequential extraction.

		**Fe [µmol/g]**								
		Step 1 $CaCl_2$	Step 2 NaAc	Step 3 HydAm	Step 4 CDB	Step 5 Oxalate	Step 6 HNO_3	Reactive Fe	Total Fe	% reactive Fe
**HSF**	Initial sediment	0	51	38	27	10	39	125	331	38
	Fh_initial	0	59	141	30	10	36	241	458	53
	FhP_initial	0	90	118	30	10	39	249	458	54
	FhP+Viv_initial	0	124	126	28	11	38	288	488	59
**HW**	Initial sediment	0	93	70	51	17	70	232	557	29
	Fh_initial	0	110	243	58	17	65	428	772	55
	FhP_initial	0	154	217	54	18	66	442	771	57
	FhP+Viv_initial	0	201	276	58	18	68	553	818	68
**FKS**	Initial sediment	0	16	11	13	5	9	44	193	23
	Fh_initial	0	25	70	16	5	10	116	271	43
	FhP_initial	0	47	34	15	6	11	102	271	38
	FhP+Viv_initial	0	72	48	16	5	11	141	289	49

Table S3 – Results of sequential Fe extraction for reacted sediment and treatments for each field site. For reacted samples, the average of the three triplicates, including their standard deviation, is reported. Reactive Fe is defined as the sum of the first five steps of the sequential extraction.

		Fe [µmol/g]						
		Step 1 $CaCl_2$	Step 2 NaAc	Step 3 HydAm	Step 4 CDB	Step 5 Oxalate	Step 6 HNO_3	Reactive Fe
**HSF**	Sediment	14	71	23	25	10	42	142
	Fh	8 ± 4	109 ± 8	47 ± 4	32 ± 3	10 ± 1	40 ± 4	206 ± 16
	FhP	12 ± 4	117 ± 2	45 ± 6	35 ± 2	11 ± 0	45 ± 2	220 ± 7
	FhP+Viv	5 ± 2	136 ± 2	45 ± 1	32 ± 2	10 ± 1	41 ± 3	228 ± 6
**HW**	Sediment	1	103	69	56	18	75	248
	Fh	20 ± 6	212 ± 6	95 ± 14	64 ± 5	20 ± 1	78 ± 4	411 ± 17
	FhP	31 ± 1	234 ± 17	58 ± 4	54 ± 1	18 ± 1	74 ± 2	395 ± 21
	FhP+Viv	20 ± 8	218 ± 12	69 ± 13	59 ± 3	19 ± 0	75 ± 2	384 ± 4
**FKS**	Sediment	0	14	11	15	6	12	46
	Fh	0 ± 0	38 ± 6	10 ± 5	15 ± 2	6 ± 1	12 ± 0	69 ± 1
	FhP	0 ± 0	39 ± 2	17 ± 4	16 ± 1	6 ± 0	12 ± 0	78 ± 6
	FhP+Viv	0 ± 0	35 ± 7	15 ± 2	17 ± 2	6 ± 1	11 ± 2	73 ± 10

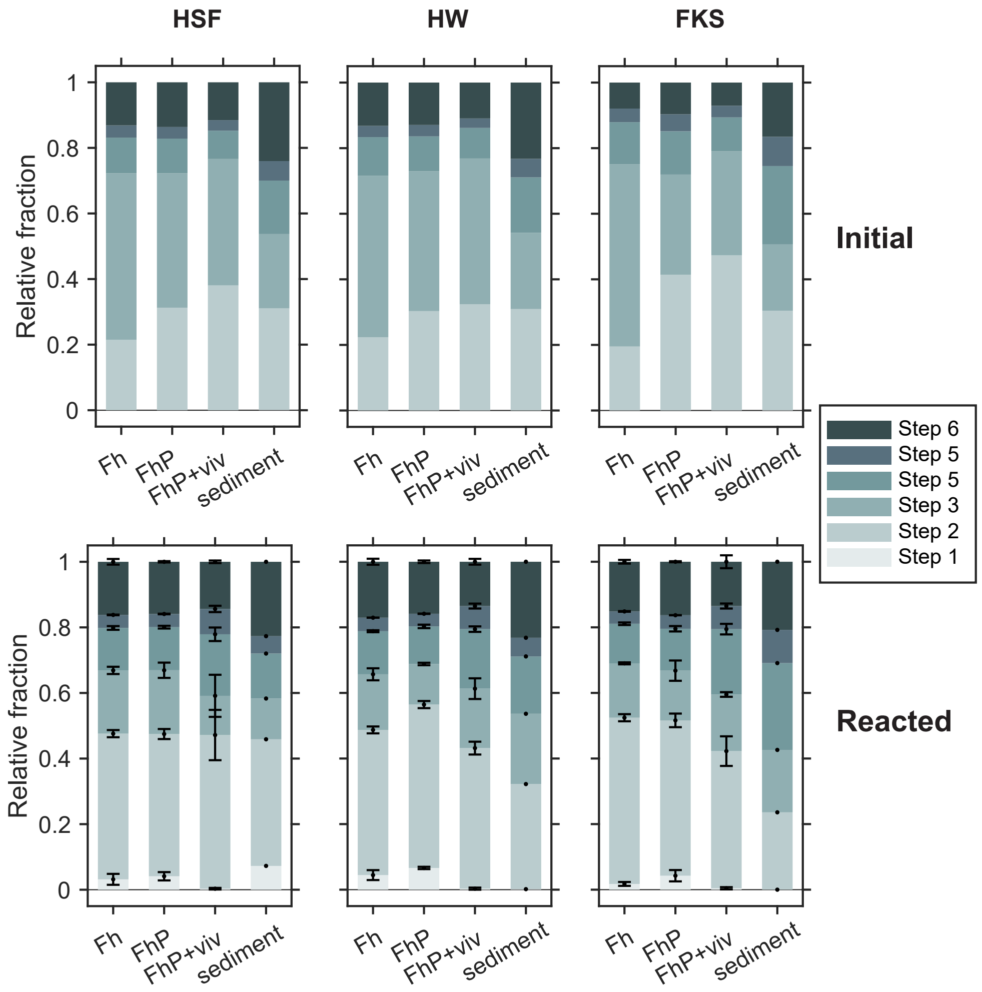

Figure S17 – Distribution of Fe phases in the samples classified by sequential Fe extraction from the initial and reacted solid phase material for each field site and treatment. Reacted samples were extracted in triplicate, and error bars indicate the standard deviation.

Interestingly, the sum of extractable Fe increased somewhat during the experimental period in unamended sediment samples (controls, compare Table S2 with Table S3). In contrast, the extractable Fe for the treatments decreased during the experiment. This decrease in extractable Fe may indicate a loss of the added mineral phases during the experiment. The reductive dissolution of ferrihydrite likely resulted in the formation of aqueous Fe(II), which could diffuse or be transported by advective flow out of the mesh-bags, leading to an overall loss of Fe. Notable differences existed between the sediments of each field site, which appears to be closely related to the sediment texture. The finer the natural sediment structure, the less of the added mineral phases was lost. HW had clay/silty sediment, and except for FhP+Viv, extractable Fe barely changed, indicating no major losses of the added mineral phases. HSF sediment with its silty/ fine sandy sediment had a minor loss in extractable Fe, while a significant amount of Fe was lost from the coarser sandy FKS sediment. This observation might also explain why FKS ^{57}Fe-Mössbauer spectra were more noisy since a greater portion of the added mineral phase was lost. Nevertheless, even for FKS, substantial amounts of added ^{57}Fe were present so that between 77 to 80 % of the ^{57}Fe-Mössbauer signal still originate from the added ^{57}Fe based on conservative calculations.

Table S4 – Changes in extractable Fe in initial and reacted solid-phase material of the samples. Extractable Fe represents the sum of the Fe extracted by the six-step sequential Fe extraction. Based on the differences in extractable Fe, it was possible to estimate the amount of ^{57}Fe remaining in the sample after incubation and determine the minimum contribution of the added ^{57}Fe to the Mössbauer (MB) spectrum.

**Sample**	**Extractable Fe initial solid-phase** [µmol/g]	**Extractable Fe reacted solid-phase** [µmol/g]	**Changes in extractable Fe** [%]	**Amount of ^{57}Fe in reacted solid-phase** [µmol/g]	**Contribution of added ^{57}Fe to MB spectra** [%]
HSF sediment	164	184	+12		
HSF Fh	277	245	-12	46	86
HSF FhP	288	265	-8	52	88
HSF FhP+Viv	326	268	-18	53	88
HW sediment	302	323	+7		
HW Fh	493	489	-1	107	89
HW FhP	508	469	-8	85	87
HW FhP+Viv	621	459	-26	80	85
FKS sediment	53	58	+9		
FKS Fh	125	81	-35	16	77
FKS FhP	113	90	-20	19	80
FKS FhP+Viv	152	84	-45	16	77

S4.2 Mössbauer data - reacted samples

S4.2.1 Fitted spectra and hyperfine parameters of reacted samples

Reacted Fh samples

Mössbauer spectra of reacted Fh samples from all three field sites differed strongly from the initial Fh sample. A second doublet (Fe(II)D1) emerged at 77 K, requiring fitting parameters of CS and QS values of $\sim$ 1.28 mm s^{-1} and $\sim$ 2.87 mm s^{-1}, respectively (Fig. 2, S18, S19, S20, Tables S5-S13), compatible with Fe(II) species such as clay minerals,[40] adsorbed Fe(II),[36] green rust,[11,19] and vivianite.[18] The Fe(II) doublet (Fe(II)D1) contributed 62%, 74%, and 51% of the spectral area at HSF, HW, and FKS, respectively. Additionally, the QS value of Fe(III)D decreased from $\sim$ 0.90 mm s^{-1} to a range of $\sim$ 0.51 to 0.69 mm s^{-1} (Fig. S18, S19, S20, Tables S5-S13), indicating mineralogical changes in the remaining Fe(III), potentially associated with the presence of green rust[19,10] or pyrite.[34] The 77 K Mössbauer spectrum of FKS additionally contained a collapsed feature (Fig. S20, Tables S11) with hyperfine parameters similar to FeS_x minerals.[34]

The 25 K Mössbauer spectra of reacted Fh samples resembled the 77 K spectra but included a collapsed feature in all fits (Fig. S18, S19, S20) , resulting in a reduction in the spectral area of both doublets (Fe(II)D1 and Fe(III)D). This decrease (Fig. S24) suggests magnetic ordering of some Fe(II), possibly indicating the presence of siderite (Néel temperature of $\sim$ 37 K).[7]

At 13 and 10 K Mössbauer spectra of reacted Fh samples displayed a small sextet (Fe(III)S1), with hyperfine parameters consistent with ferrihydrite and lepidocrocite (Table S5-S13).[36,23] The 5 K Mössbauer spectra were dominated by an octet, Fe(II)D1, Fe(III)S1, and a second sextet (Fe(III)S2) across all sites. The emergence of a second sextet below 10 K aligns with the ordering temperature of the Fe(III) component of green rust ($\sim$ 7 K).[31] Additionally, the hyperfine parameters of the sextet were in the range reported for green rust.[23] The formation of an octet below 10 K is aligning with the magnetic ordering temperature of Fe(II) bound in green rust (Néel temperature $\sim$ 5.2 K).[31] Thus, these are strong indications pointing towards the presence of green rust at all field sites. The presence of an Fe(II) doublet at 5 K and the ordering of some Fe(II) at 25 K suggests that adsorbed Fe(II) and likely siderite were also present. Overlaying the collected Mössbauer spectra with a reference siderite spectrum at 5 K also indicates that the features present at 5 K could match siderite (Fig. S21, S22). This implies that the fitted octet was likely oversimplified, representing a mixture of multiple phases.

In summary, the Mössbauer spectra of reacted Fh samples highlight the extensive reduction of added Fe(III) to Fe(II) (51 to 74 % reduced at the end of the experiment, Table S21). The remaining Fe(III) likely existed as green-rust, ferrihydrite, or lepidocrocite, while the formed Fe(II) was probably bound in green-rust, siderite, and adsorbed Fe(II) across all sites. At the high salinity site (FKS), the data suggest the presence of Fe-sulfide minerals in addition to other reduced mineral

phases.

Reacted FhP(+Viv) samples

Similar to Fh samples, reacted FhP(+Viv) samples contained 38% to 81% of the ^{57}Fe as Fe(II) (Table S21). The Mössbauer spectra of Fh and FhP(+Viv) samples exhibited similarities, especially for FKS, indicating the formation of similar secondary minerals. However, differences existed for samples from HSF and HW. In the reacted FhP samples at HSF and HW, an additional doublet (Fe(II)D2) was required in the 77 K spectra fit (Fig. S18, S19). The doublet was required as fitting with only one doublet (Fe(II)D1) resulted in an unusually high standard deviation for the doublet's quadrupole splitting. Additionally, a slight shoulder was visible in the doublet feature at $\sim$ 3 mm s^{-1}, indicative of the presence of two doublets present in the feature (Fig.2). Thus, for FhP(+Viv) samples from HSF and HW a second doublet (Fe(II)D2) was included in the fit. This doublet had CS and QS values of $\sim$ 1.30 mm s^{-1} and $\sim$ 3.20 mm s^{-1}, consistent with Fe(II) bound in the double octahedral position in vivianite.[19,18] The Fe(II)D2 doublet contributed 27 and 18 % to the spectral area at HSF and HW, respectively (Tables S6, S9). The collapsed feature at 25 K contributed less area for FhP samples than for Fh samples at both field sites. Additionally, the decrease in the summed spectral area of Fe(II)D1 and D2 observed from 77 K to 25 K was marginal (Fig. S24), implying that no magnetic ordering representative of siderite occurred. No sextet formed at 13 and 10 K, while the collapsed feature increased at 10 K. This aligns with the magnetic ordering temperature of vivianite (Néel temperature $\sim$ 12 K),[22] which forms multiple octets below 12 K. At 5 K, the spectral area was dominated by an area that could be fitted as an octet (constituting 75 % and 63 % of the 5 K spectra's area at HSF and HW, respectively). However, the magnetic ordering temperatures of different components suggest the presence of multiple mineral phases, including green rust and vivianite, which form octets below 10 K. Consequently, the fitted octet likely comprised multiple octets and is oversimplified. For instance, a proportion of Fe(II) exhibited ordering only at 5 K, aligning with the magnetic ordering temperature of Fe(II) bound green rust.[31] Additionally, at 5 K, distinct features were visible, notably a sharp peak at -4 mm s^{-1} Overlaying the collected spectra with a reference vivianite spectrum at 5 K indicated that this peak resembled vivianite (Fig. S21, S23). Hence, the area fitted as an octet at 5 K at HSF and HW likely represented Fe(II) in green rust and vivianite. Overall, the data suggests the presence of vivianite and green rust and the absence of siderite in reacted FhP samples incubated at HSF and HW.

Reacted FhP+Viv Mössbauer spectra resembled FhP samples at HSF and HW. The spectral area of Fe(II)D2, matching hyperfine parameters of vivianite, changed from 27 to 23% for HSF, but increased from 18 to 24% for HW (Tables S6, S7, S8, S10). This increase was paired with a more pronounced peak at $\sim$ -4 mm s^{-1} in HW's FhP+Viv 5 K spectrum, a representative feature

of vivianite (Fig. S21, S23).

In summary, HSF and HW's FhP and FhP+Viv treatments contained Fe(II) bound in vivianite, green rust, and minor amounts of adsorbed Fe(II). The remaining Fe(III) was likely present in green rust, with traces of ferrihydrite or lepidocrocite (Table S6, S7, S9, S10). For FKS, the Mössbauer spectra of the different treatments resembled each other, indicating that major transformation products were green rust, adsorbed Fe(II), Fe-sulfide minerals, ferrihydrite and/or lepidocrocite, and potentially some siderite for the FhP(+Viv) treatments.

Table S5 – Hyperfine parameters obtained for fitting of reacted HSF Fh samples at 77 K, 25 K, 13 K and 10 K using xVBF model and 5 K using Full Static Hamiltonian model.[a]

**Temp.**	**Phase**	**Phase interpretation**	**Spectral Area %**	**CS**[b] **[mm s**$^{-1}$**]**	**QS**[c] **or ε [mm s**$^{-1}$**]**	**H**[d] **[T]**	**σ**[e] **[mm s**$^{-1}$**] or [T]**	$\mathbf{e^2qQ/2}$[f] **[mm s**$^{-1}$**]**	**η**[g] **[mm s**$^{-1}$**]**	**w**[h] **[mm s**$^{-1}$**]**	**φ**[i] **[°]**	**ϑ**[j] **[°]**	**Red-**$\chi^{2,k}$
77 K	Fe(III) D	Fe(III) (SRO)oxides, GR. or Pyr.	37.9	0.50<br>(0.01)	0.66<br>(0.01)		0.35<br>(0.02)						*0.87*
	Fe(II) D1	Fe(II) in GR, sorbed, Fe(II) min.	62.1	1.28<br>(0.00)	2.87<br>(0.01)		0.31<br>(0.01)						
25 K	Fe(III) D	Fe(III) (SRO)oxides, GR. or Pyr.	25.8	0.47<br>(0.02)	0.69<br>(0.03)		**0.42**						*0.64*
	Fe(II) D1	Fe(II) in GR, sorbed, Fe(II) min.	46.0	1.30<br>(0.01)	2.87<br>(0.01)		**0.31**						
	Collapsed	Fe(II) in Sid., Fe(III) in oxides	28.2	1.40<br>(0.29)	1.56<br>(0.42)		**6.11**						
13 K	Fe(III) D	Fe(III) (SRO)oxides, GR. or Pyr.	14.4	0.44<br>(0.02)	0.65<br>(0.03)		0.37<br>(0.05)						*0.73*
	Fe(II) D1	Fe(II) in GR, sorbed, Fe(II) min.	31.4	1.30<br>(0.01)	2.85<br>(0.01)		0.33<br>(0.02)						
	Fe(III) S1	Fh-like, Lp-like	14.4	0.45<br>(0.05)	-0.01<br>(0.05)	47.64<br>(0.46)	3.54<br>(0.53)						
	Collapsed	Fe(II) in Sid., Fe(III) in oxides	39.8	1.22<br>(0.09)	3.51<br>(0.16)		3.20<br>(0.26)						
10 K	Fe(III) D	Fe(III) (SRO)oxides, GR. or Pyr.	10.3	**0.44**	**0.65**		**0.37**						*0.62*
	Fe(II) D1	Fe(II) in GR, sorbed, Fe(II) min.	20.3	**1.30**	**2.85**		**0.33**						
	Fe(III) S1	Fh-like, Lp-like	19.1	**0.45**	**-0.01**	**47.64**	**3.54**						
	Collapsed	Fe(II) in Sid., Fe(III) in oxides, GR.	50.3	**1.22**	**3.53**		**3.20**						
5 K	Fe(II) D1	Fe(II) sorbed, GR - unordered	10.9	1.39<br>(0.02)				2.70<br>(0.04)	**0**	**0.33**	**0**	**0**	*1.79*
	Fe(III) S1	Fh-like, Lp-like	9.5	0.41<br>(0.02)		44.88<br>(0.52)		0.15<br>(0.01)	**0**	**0.45**	**0**	**0**	
	Fe(III) S2	Fh-like, Fe(III) in GR.	13.1	0.60<br>(0.04)		50.68<br>(0.31)		-0.27<br>(0.03)	**0**	**0.41**	**0**	**0**	
	Octet	Fe(II) in GR., Sid., unk.	66.4	1.44<br>(0.02)		13.01<br>(0.02)		-2.88<br>(0.05)	**0.2**	**0.76**	**84**	**95**	

[a]Parameter uncertainties are presented for the last significant figures. Bold values represent fixed values during fitting.;
[b]Center shift;
[c]Quadrupole splitting (QS, for doublets) or Quadrupole shift (ε, for sextet);
[d]Hyperfine field;
[e]Standard deviation of QS (doublets) or H (sextet);
[f]Quadrupole splitting in paramagnetic state or quadrupole shift;
[g]Asymmetry parameter;
[h]Half line width at half maximum;
[i]Azimuthal angle between the electric field gradient axis of symmetry with hyperfine field H;
[j]Polar angle between the electric field gradient axis of symmetry with hyperfine field H;
[k]Goodness of fit;
Abbreviations: Temp.= Temperature; Fe(III) D = Fe(III) doublet; Fe(II) D1= Fe(II) doublet; Fe(III) S1= Fe(III) sextet; min. = minerals; pyr. = pyrite; Fh = ferrihydrite; Lp = lepidocrocite; SRO = short ranged ordered; unk. = unknown, not clearly identifiable; Gt = (nano)goethite; GR = green rust-like phase; FeS_x = iron sulfide minerals such as mackinawite and greigite; Sid. = siderite.

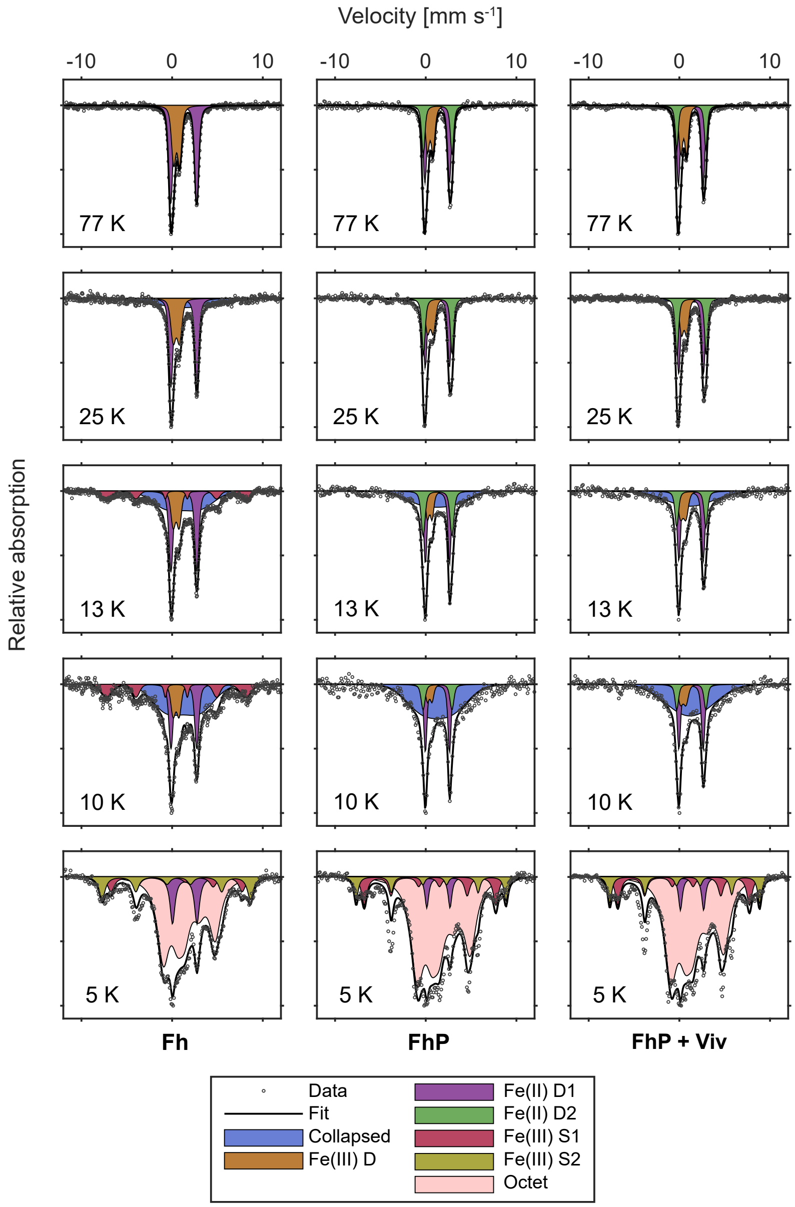

Figure S18 – Fitted Mössbauer spectra of reacted HSF samples. Note, that the fitted octet is oversimplified and likely contains multiple collapsed octets as described in Section S4.2. Fitted hyperfine parameters are presented in Tables S5-S7. Interpretation of fitted components: Fe(III) D = doublet of solid-phase Fe(III) likely present as Fe-oxides and/or green rust; Fe(II) D1 = doublet of solid-phase Fe(II) likely present in green rust, clay minerals, siderite, vivianite and/or adsorbed Fe(II); Fe(II) D2 = doublet of solid-phase Fe(II) likely present in double-octahedral position in vivianite; Fe(III) S1 = sextet of solid-phase Fe(III) likely present in (short-ranged) Fe-oxide minerals such as ferrihydrite and lepidocrocite; Fe(III) S2 = sextet of solid-phase Fe(III) likely present in green rust and/or ferrihydrite; Octet = likely a mixture of iron minerals including Fe(II)-bound in green rust, siderite and/or vivianite; Collapsed = depending on temperature of data collection and mineral specific Néel temperatures feature represents different mineral phases such as FeS_x, siderite, vivianite and Fe(III) in Fe-oxides or green rust.

Table S6 – Hyperfine parameters obtained for fitting of HSF FhP sample at 77 K, 25 K, 13 K and 10 K using xVBF model and 5 K using Full Static Hamiltonian model.[a]

**Temp.**	**Phase**	**Phase interpretation**	**Spectral Area %**	**CS**[b] **[mm s^{-1}]**	**QS**[c] **or ε [mm s^{-1}]**	**H**[d] **[T]**	**σ**[e] **[mm s^{-1}] or [T]**	**$e^2qQ/2$**[f] **[mm s^{-1}]**	**η**[g] **[mm s^{-1}]**	**w**[h] **[mm s^{-1}]**	**φ**[i] **[°]**	**ϑ**[j] **[°]**	**Red-$\chi^{2,k}$**
77 K	Fe(III) D	Fe(III) (SRO)oxides, GR. or Pyr.	27.6	0.50 (0.02)	0.61 (0.03)		**0.30**						*0.62*
	Fe(II) D1	Fe(II) in GR, sorbed, Viv., Sid.	45.35	1.26 (0.01)	2.70 (0.04)		**0.26**						
	Fe(II) D2	Fe(II) in Viv.	27.1	1.31 (0.01)	3.17 (0.05)		**0.21**						
25 K	Fe(III) D	Fe(III) (SRO)oxides, GR. or Pyr.	22.4	0.52 (0.03)	0.63 (0.04)		**0.37**						*0.58*
	Fe(II) D1	Fe(II) in GR, sorbed, Viv., Sid.	38.1	1.28 (0.01)	2.67 (0.04)		**0.24**						
	Fe(II) D2	Fe(II) in Viv.	29.7	1.33 (0.01)	3.15 (0.05)		**0.22**						
	Collapsed	Fe(II) in Sid., Fe(III) in oxides	9.9	**1.4**	**3.50**		**4.55**						
13 K	Fe(III) D	Fe(III) (SRO)oxides, GR. or Pyr.	11.4	0.44 (0.05)	0.51 (0.06)		**0.25**						*0.54*
	Fe(II) D1	Fe(II) in GR, sorbed, Viv.	28.5	1.29 (0.01)	2.66 (0.04)		**0.19**						
	Fe(II) D2	Fe(II) in Viv.	21.4	1.30 (0.02)	3.18 (0.10)		**0.30**						
	Collapsed	Fe(II) in Viv., Sid., Fe(III) in oxides	38.8	1.25 (0.23)	3.27 (0.42)		**3.50**						
10 K	Fe(III) D	Fe(III) (SRO)oxides, GR. or Pyr.	5.6	**0.44**	**0.51**		**0.25**						*0.62*
	Fe(II) D1	Fe(II) in GR, sorbed, Viv.	20.9	**1.29**	**2.66**		**0.19**						
	Fe(II) D2	Fe(II) in Viv.	9.4	**1.30**	**3.18**		**0.30**						
	Collapsed	Fe(II) in Viv., Sid., Fe(III) in oxides, GR.	64.2	**1.25**	**3.27**		**3.50**						
5 K	Fe(II) D1	Fe(II) sorbed, GR - unordered	4.8	1.40 (0.02)				2.56 (0.05)	**0**	**0.25**	**0**	**0**	*7.96*
	Fe(III) S1	Fh-like, Lp-like	12.3	0.42 (0.02)		45.00 (0.25)		0.11 (0.05)	**0**	**0.35**	**0**	**0**	
	Fe(III) S2	Fh-like, Fe(III) in GR.	8.1	0.80 (0.02)		51.27 (0.26)		-0.40 (0.06)	**0**	**0.26**	**0**	**0**	
	Octet	Fe(II) in GR., Viv., Sid., unk.	74.8	1.52 (0.02)		13.13 (0.13)		-2.97 (0.04)	**0.2**	**0.81**	**84**	**95**	

[a]Parameter uncertainties are presented for the last significant figures. Bold values represent fixed values during fitting.;
[b]Center shift;
[c]Quadrupole splitting (QS, for doublets) or Quadrupole shift (ε, for sextet);
[d]Hyperfine field;
[e]Standard deviation of QS (doublets) or H (sextet);
[f]Quadrupole splitting in paramagnetic state or quadrupole shift;
[g]Asymmetry parameter;
[h]Half line width at half maximum;
[i]Azimuthal angle between the electric field gradient axis of symmetry with hyperfine field H;
[j]Polar angle between the electric field gradient axis of symmetry with hyperfine field H;
[k]Goodness of fit;
Abbreviations: Temp.= Temperature; Fe(III) D = Fe(III) doublet; Fe(II) D1/D2= Fe(II) doublet; Fe(III) S1= Fe(III) sextet; min. = minerals; pyr. = pyrite; Fh = ferrihydrite; Lp = lepidocrocite; SRO = short ranged ordered; unk. = unknown, not clearly identifiable; Gt = (nano)goethite; GR = green rust-like phase; FeS_x = iron sulfide minerals such as mackinawite and greigite; Sid. = siderite; Viv. = vivianite.

Table S7 – Hyperfine parameters obtained for fitting of HSF FhP+Viv sample at 77 K, 25 K, 13 K and 10 K using xVBF model and 5 K using Full Static Hamiltonian model.[a]

Temp.	Phase	Phase interpretation	Spectral Area %	CS[b] [mm s^{-1}]	QS[c] or ε [mm s^{-1}]	H[d] [T]	σ[e] [mm s^{-1}] or [T]	$e^2qQ/2$[f] [mm s^{-1}]	η[g] [mm s^{-1}]	w[h] [mm s^{-1}]	φ[i] [°]	ϑ[j] [°]	Red-χ^{2},[k]
77 K	Fe(III) D	Fe(III) (SRO)oxides, GR. or Pyr.	29.8	0.48 (0.01)	0.65 (0.02)		**0.29**						
	Fe(II) D1	Fe(II) in GR, sorbed, Viv., Sid.	47.1	1.26 (0.01)	2.73 (0.02)		**0.24**						*0.64*
	Fe(II) D2	Fe(II) in Viv.	23.1	1.32 (0.01)	3.20 (0.03)		**0.17**						
25 K	Fe(III) D	Fe(III) (SRO)oxides, GR. or Pyr.	20.3	0.53 (0.02)	0.58 (0.02)		**0.32**						
	Fe(II) D1	Fe(II) in GR, sorbed, Viv., Sid.	39.1	1.29 (0.00)	2.72 (0.02)		**0.24**						*0.76*
	Fe(II) D2	Fe(II) in Viv.	28.3	1.35 (0.01)	3.23 (0.02)		**0.22**						
	Collapsed	Fe(II) in Sid., Fe(III) in oxides	12.2	**1.40**	**3.50**		**4.55**						
13 K	Fe(III) D	Fe(III) (SRO)oxides, GR. or Pyr.	15.0	0.43 (0.07)	0.60 (0.08)		**0.38**						
	Fe(II) D1	Fe(II) in GR, sorbed, Viv.	31.5	1.29 (0.01)	2.68 (0.06)		**0.23**						*0.61*
	Fe(II) D2	Fe(II) in Viv.	16.8	1.32 (0.03)	3.21 (0.11)		**0.25**						
	Collapsed	Fe(II) in Viv., Sid., Fe(III) in oxides, GR.	36.7	1.18 (0.29)	2.52 (0.90)		**4.26**						
10 K	Fe(III) D	Fe(III) (SRO)oxides, GR. or Pyr.	8.5	**0.43**	**0.60**		**0.38**						
	Fe(II) D1	Fe(II) in GR, sorbed, Viv.	22.7	**1.29**	**2.68**		**0.23**						*0.79*
	Fe(II) D2	Fe(II) in Viv.	9.2	**1.32**	**3.21**		**0.25**						
	Collapsed	Fe(II) in Viv., Sid., Fe(III) in oxides, GR.	59.6	**1.18**	**2.52**		**4.26**						
5 K	Fe(II) D1	Fe(II) sorbed, GR - unordered	5.2	**1.40**				**2.56**	**0**	**0.25**	**0**	**0**	
	Fe(III) S1	Fh-like, Lp-like	12.3	**0.42**		**45.00**		**0.11**	**0**	**0.35**	**0**	**0**	*10.81*
	Fe(III) S2	Fh-like, Fe(III) in GR.	9.0	**0.80**		**51.27**		**-0.40**	**0**	**0.26**	**0**	**0**	
	Octet	Fe(II) in GR., Viv., Sid., unk.	73.5	**1.52**		**13.13**		**-2.97**	**0.2**	**0.81**	**84**	**95**	

[a]Parameter uncertainties are presented for the last significant figures. Bold values represent fixed values during fitting.;
[b]Center shift;
[c]Quadrupole splitting (QS, for doublets) or Quadrupole shift (ε, for sextet);
[d]Hyperfine field;
[e]Standard deviation of QS (doublets) or H (sextet);
[f]Quadrupole splitting in paramagnetic state or quadrupole shift;
[g]Asymmetry parameter;
[h]Half line width at half maximum;
[i]Azimuthal angle between the electric field gradient axis of symmetry with hyperfine field H;
[j]Polar angle between the electric field gradient axis of symmetry with hyperfine field H;
[k]Goodness of fit;
Abbreviations: Temp.= Temperature; Fe(III) D = Fe(III) doublet; Fe(II) D1/D2= Fe(II) doublet; Fe(III) S1= Fe(III) sextet; min. = minerals; pyr. = pyrite; Fh = ferrihydrite; Lp = lepidocrocite; SRO = short ranged ordered; unk. = unknown, not clearly identifiable; Gt = (nano)goethite; GR = green rust-like phase; FeS_x = iron sulfide minerals such as mackinawite and greigite; Sid. = siderite; Viv. = vivianite.

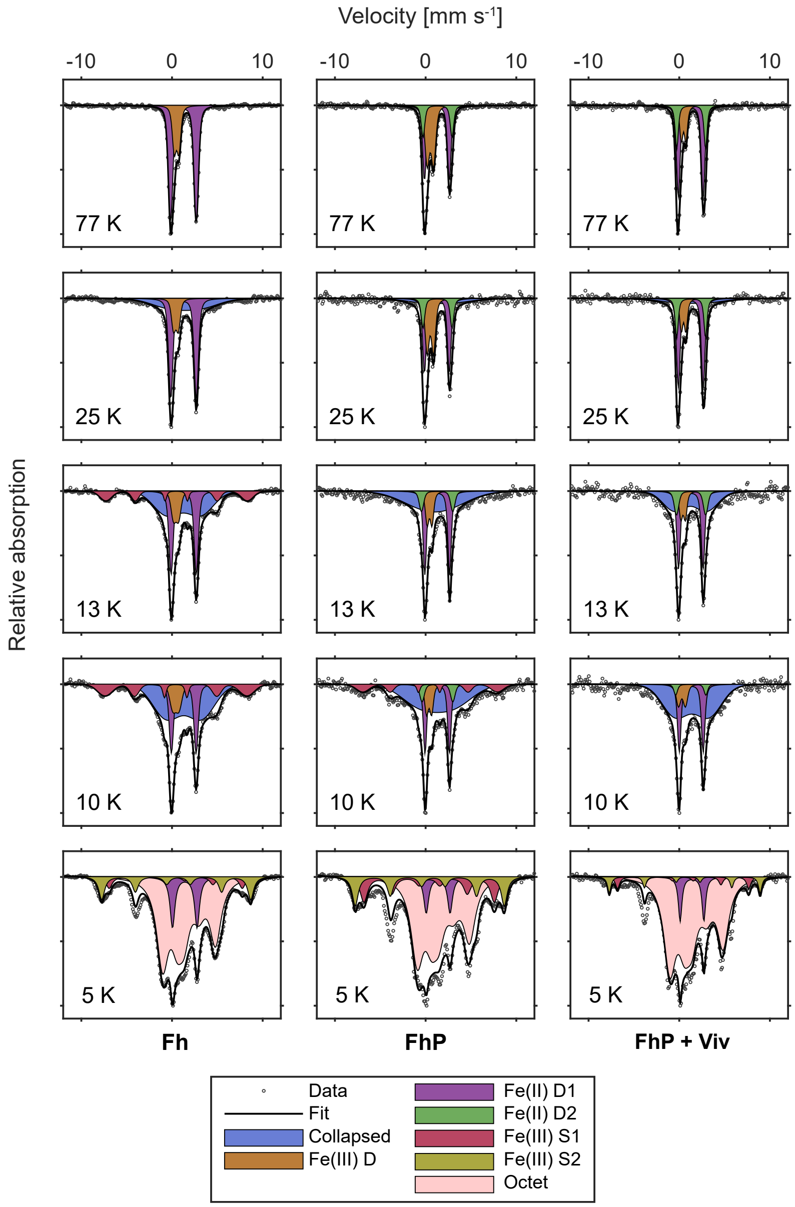

Figure S19 – Fitted Mössbauer spectra of reacted HW samples. Note, that the fitted octet is oversimplified and likely contains multiple collapsed octets as discussed in Section S4.2. Fitted hyperfine parameters are presented in Tables S8-S10. Interpretation of fitted components: Fe(III) D = doublet of solid-phase Fe(III) likely present as Fe-oxides and/or green rust; Fe(II) D1 = doublet of solid-phase Fe(II) likely present in green rust, clay minerals, siderite, vivianite and/or adsorbed Fe(II); Fe(II) D2 = doublet of solid-phase Fe(II) likely present in double-octahedral position in vivianite; Fe(III) S1 = sextet of solid-phase Fe(III) likely present in (short-ranged) Fe-oxide minerals such as ferrihydrite and lepidocrocite; Fe(III) S2 = sextet of solid-phase Fe(III) likely present in green rust and/or ferrihydrite; Octet = likely a mixture of iron minerals including Fe(II)-bound in green rust, siderite and/or vivianite; Collapsed = depending on temperature of data collection and mineral specific Néel temperatures feature represents different mineral phases such as FeS_x, siderite, vivianite and Fe(III) in Fe-oxides or green rust.

Table S8 – Hyperfine parameters obtained for fitting of HW Fh sample at 77 K, 25 K, 13 K and 10 K using xVBF model and 5 K using Full Static Hamiltonian model.[a]

**Temp.**	**Phase**	**Phase interpretation**	**Spectral Area %**	**CS[b]** [mm s^{-1}]	**QS[c] or ε** [mm s^{-1}]	**H[d]** [T]	**σ[e]** [mm s^{-1}] or [T]	**e^{2}qQ/2[f]** [mm s^{-1}]	**η[g]** [mm s^{-1}]	**w[h]** [mm s^{-1}]	**φ[i]** [°]	**ϑ[j]** [°]	**Red-χ^{2,k}**
77 K	Fe(III) D	Fe(III) (SRO)oxides, GR. or Pyr.	26.1	0.50 (0.01)	0.51 (0.01)		0.29 (0.02)						*1.42*
	Fe(II) D1	Fe(II) in GR, sorbed, Fe(II) min.	73.9	1.26 (0.00)	2.82 (0.01)		0.35 (0.01)						
25 K	Fe(III) D	Fe(III) (SRO)oxides, GR. or Pyr.	15.2	0.43 (0.02)	0.53 (0.03)		**0.31**						*1.01*
	Fe(II) D1	Fe(II) in GR, sorbed, Fe(II) min.	52.6	1.27 (0.01)	2.81 (0.01)		**0.35**						
	Collapsed	Fe(II) in Sid., Fe(III) in oxides	32.2	1.43 (0.16)	3.49 (0.31)		**3.86**						
13 K	Fe(III) D	Fe(III) (SRO)oxides, GR. or Pyr.	10.5	0.44 (0.03)	0.56 (0.03)		**0.40**						*1.23*
	Fe(II) D1	Fe(II) in GR, sorbed, Fe(II) min.	29.5	1.29 (0.01)	2.78 (0.01)		0.35 (0.02)						
	Fe(III) S1	Fh-like, Lp-like	16.5	0.49 (0.04)	0.02 (0.04)	48.48 (0.34)	3.69 (0.38)						
	Collapsed	Fe(II) in GR, sorbed, Fe(II) min.	43.5	1.26 (0.06)	3.61 (0.10)		2.82 (0.13)						
10 K	Fe(III) D	Fe(III) (SRO)oxides, GR. or Pyr.	9.0	0.43 (0.06)	0.68 (0.07)		**0.54**						*1.32*
	Fe(II) D1	Fe(II) in GR, sorbed, Fe(II) min.	19.7	1.30 (0.01)	2.73 (0.02)		0.35 (0.02)						
	Fe(III) S1	Fh-like, Lp-like	19.6	0.42 (0.04)	0.02 (0.04)	48.55 (0.38)	4.79 (0.42)						
	Collapsed	Fe(II) in Sid., Fe(III) in oxides, GR.	51.7	1.30 (0.05)	3.79 (0.10)		2.93 (0.13)						
5 K	Fe(II) D1	Fe(II) sorbed, GR - unordered	11.2	1.41 (0.00)				2.71 (0.00)	**0**	0.32 (0.01)	**0**	**0**	*8.88*
	Fe(III) S1	Fh-like, Lp-like	6.4	0.34 (0.02)		45.52 (0.22)		0.15 (0.06)	**0**	0.40 (0.02)	**0**	**0**	
	Fe(III) S2	Fh-like, Fe(III) in GR.	12.5	0.59 (0.01)		50.95 (0.10)		-0.25 (0.03)	**0**	0.39 (0.01)	**0**	**0**	
	Octet	Fe(II) in GR., Sid., unk.	69.9	1.42 (0.01)		13.34 (0.05)		-2.92 (0.01)	**0.2**	0.76 (0.01)	**84**	**95**	

[a]Parameter uncertainties are presented for the last significant figures. Bold values represent fixed values during fitting.;
[b]Center shift;
[c]Quadrupole splitting (QS, for doublets) or Quadrupole shift (ε, for sextet);
[d]Hyperfine field;
[e]Standard deviation of QS (doublets) or H (sextet);
[f]Quadrupole splitting in paramagnetic state or quadrupole shift;
[g]Asymmetry parameter;
[h]Half line width at half maximum;
[i]Azimuthal angle between the electric field gradient axis of symmetry with hyperfine field H;
[j]Polar angle between the electric field gradient axis of symmetry with hyperfine field H;
[k]Goodness of fit;
Abbreviations: Temp.= Temperature; Fe(III) D = Fe(III) doublet; Fe(II) D1= Fe(II) doublet; Fe(III) S1= Fe(III) sextet; min. = minerals; pyr. = pyrite; Fh = ferrihydrite; Lp = lepidocrocite; SRO = short ranged ordered; unk. = unknown, not clearly identifiable; Gt = (nano)goethite; GR = green rust-like phase; FeS_x = iron sulfide minerals such as mackinawite and greigite; Sid. = siderite.

Table S9 – Hyperfine parameters obtained for fitting of HW FhP sample at 77 K, 25 K, 13 K and 10 K using xVBF model and 5 K using Full Static Hamiltonian model.[a]

**Temp.**	**Phase**	**Phase interpretation**	**Spectral Area %**	**CS[b]** [mm s^{-1}]	**QS[c] or ε** [mm s^{-1}]	**H[d]** [T]	**σ[e]** [mm s^{-1}] or [T]	**e^{2}qQ/2[f]** [mm s^{-1}]	**η[g]** [mm s^{-1}]	**w[h]** [mm s^{-1}]	**φ[i]** [°]	**ϑ[j]** [°]	**Red-χ^{2},[k]**
77 K	Fe(III) D	Fe(III) (SRO)oxides, GR. or Pyr.	39.3	0.52 (0.01)	0.70 (0.02)		**0.37**						*0.81*
	Fe(II) D1	Fe(II) in GR, sorbed, Viv., Sid.	42.8	1.24 (0.01)	2.74 (0.03)		**0.26**						
	Fe(II) D2	Fe(II) in Viv.	17.9	1.31 (0.02)	3.20 (0.06)		**0.25**						
25 K	Fe(III) D	Fe(III) (SRO)oxides, GR. or Pyr.	30.6	**0.52**	**0.70**		**0.32**						*0.70*
	Fe(II) D1	Fe(II) in GR, sorbed, Viv., Sid.	38.6	**1.24**	**2.74**		**0.26**						
	Fe(II) D2	Fe(II) in Viv.	15.2	**1.31**	**3.20**		**0.25**						
	Collapsed	Fe(II) in Sid., Fe(III) in oxides	15.8	**1.43**	**3.50**		**3.90**						
13 K	Fe(III) D	Fe(III) (SRO)oxides, GR. or Pyr.	12.2	0.43 (0.04)	0.53 (0.04)		**0.25**						*0.76*
	Fe(II) D1	Fe(II) in GR, sorbed, Viv.	30.6	1.28 (0.01)	2.73 (0.03)		**0.23**						
	Fe(II) D2	Fe(II) in Viv.	9.3	1.27 (0.05)	3.46 (0.15)		**0.35**						
	Collapsed	Fe(II) in Viv., Sid., Fe(III) in oxides	48.0	1.01 (0.19)	2.96 (0.61)		**4.95**						
10 K	Fe(III) D	Fe(III) (SRO)oxides, GR. or Pyr.	8.7	0.38 (0.05)	0.56 (0.07)		**0.27**						*0.60*
	Fe(II) D1	Fe(II) in GR, sorbed, Viv.	19.7	1.28 (0.01)	2.69 (0.05)		**0.23**						
	Fe(II) D2	Fe(II) in Viv.	6.3	1.30 (0.07)	3.39 (0.23)		**0.37**						
	Fe(III) S1	Fh-like, Lp-like	13.6	0.44 (0.11)	0.05 (0.11)	46.02 (1.00)	**4.00**						
	Collapsed	Fe(II) in Viv., Sid., Fe(III) in oxides	51.7	1.26 (0.15)	3.68 (0.31)		**4.02**						
5 K	Fe(II) D1	Fe(II) sorbed, GR - unordered	7.2	1.38 (0.01)				2.63 (0.03)	**0**	**0.32**	**0**	**0**	*7.96*
	Fe(III) S1	Fh-like, Lp-like	15.8	0.41 (0.02)		44.58 (0.21)		-0.05 (0.04)	**0**	**0.50**	**0**	**0**	
	Fe(III) S2	Fh-like, Fe(III) in GR.	14.4	0.66 (0.02)		50.95 (0.11)		-0.40 (0.04)	**0**	**0.39**	**0**	**0**	
	Octet	Fe(II) in GR., Viv., Sid., unk.	62.6	1.50 (0.01)		12.99 (0.10)		-2.93 (0.03)	**0.2**	**0.80**	**84**	**95**	

[a]Parameter uncertainties are presented for the last significant figures. Bold values represent fixed values during fitting.;
[b]Center shift;
[c]Quadrupole splitting (QS, for doublets) or Quadrupole shift (ε, for sextet);
[d]Hyperfine field;
[e]Standard deviation of QS (doublets) or H (sextet);
[f]Quadrupole splitting in paramagnetic state or quadrupole shift;
[g]Asymmetry parameter;
[h]Half line width at half maximum;
[i]Azimuthal angle between the electric field gradient axis of symmetry with hyperfine field H;
[j]Polar angle between the electric field gradient axis of symmetry with hyperfine field H;
[k]Goodness of fit;
Abbreviations: Temp.= Temperature; Fe(III) D = Fe(III) doublet; Fe(II) D1/D2= Fe(II) doublet; Fe(III) S1= Fe(III) sextet; min. = minerals; pyr. = pyrite; Fh = ferrihydrite; Lp = lepidocrocite; SRO = short ranged ordered; unk. = unknown, not clearly identifiable; Gt = (nano)goethite; GR = green rust-like phase; FeS_x = iron sulfide minerals such as mackinawite and greigite; Sid. = siderite; Viv. = vivianite.

Table S10 – Hyperfine parameters obtained for fitting of HW FhP+Viv sample at 77 K, 25 K, 13 K and 10 K using xVBF model and 5 K using Full Static Hamiltonian model.[a]

**Temp.**	**Phase**	**Phase interpretation**	**Spectral Area %**	**CS[b]** [mm s^{-1}]	**QS[c] or ε** [mm s^{-1}]	**H[d]** [T]	**σ[e]** [mm s^{-1}] or [T]	**$e^2qQ/2$[f]** [mm s^{-1}]	**η[g]** [mm s^{-1}]	**w[h]** [mm s^{-1}]	**φ[i]** [°]	**ϑ[j]** [°]	**Red-χ^{2}[k]**
77 K	Fe(III) D	Fe(III) (SRO)oxides, GR. or Pyr.	19.1	0.48 (0.02)	0.51 (0.03)		**0.20**						*0.56*
	Fe(II) D1	Fe(II) in GR, sorbed, Viv., Sid.	56.8	1.27 (0.01)	2.74 (0.03)		**0.26**						
	Fe(II) D2	Fe(II) in Viv.	24.1	1.31 (0.01)	3.18 (0.05)		**0.19**						
25 K	Fe(III) D	Fe(III) (SRO)oxides, GR. or Pyr.	15.6	**0.48**	**0.51**		**0.20**						*0.71*
	Fe(II) D1	Fe(II) in GR, sorbed, Viv., Sid.	38.6	**1.27**	**2.74**		**0.26**						
	Fe(II) D2	Fe(II) in Viv.	19.2	**1.31**	**3.18**		**0.19**						
	Collapsed	Fe(II) in Sid., Fe(III) in oxides	16.3	**1.43**	**3.50**		**3.90**						
13 K	Fe(III) D	Fe(III) (SRO)oxides, GR. or Pyr.	10.9	0.42 (0.07)	0.49 (0.09)		**0.23**						*0.55*
	Fe(II) D1	Fe(II) in GR, sorbed, Viv.	32.9	1.28 (0.02)	2.69 (0.07)		**0.25**						
	Fe(II) D2	Fe(II) in Viv.	12.4	1.31 (0.07)	3.22 (0.38)		**0.37**						
	Collapsed	Fe(II) in Viv., Sid., Fe(III) in oxides, GR.	43.8	1.25 (0.20)	3.52 (0.21)		**2.42**						
10 K	Fe(III) D	Fe(III) (SRO)oxides, GR. or Pyr.	8.7	0.30 (0.20)	0.76 (0.39)		**0.36**						*0.74*
	Fe(II) D1	Fe(II) in GR, sorbed, Viv.	22.7	1.32 (0.05)	2.54 (0.06)		**0.25**						
	Fe(II) D2	Fe(II) in Viv.	3.7	1.27 (0.14)	3.32 (0.40)		**0.25**						
	Collapsed	Fe(II) in Viv., Sid., Fe(III) in oxides, GR.	64.9	1.41 (0.11)	3.62 (0.17)		**2.79**						
5 K	Fe(II) D1	Fe(II) sorbed, GR - unordered	7.9	1.41 (0.01)				2.62 (0.03)	**0**	**0.24**	**0**	**0**	*6.98*
	Fe(III) S1	Fh-like, Lp-like	4.9	0.42 (0.04)		44.87 (0.22)		0.02 (0.09)	**0**	**0.27**	**0**	**0**	
	Fe(III) S2	Fh-like, Fe(III) in GR.	5.6	0.79 (0.03)		51.59 (0.30)		-0.38 (0.06)	**0**	**0.22**	**0**	**0**	
	Octet	Fe(II) in GR., Viv., Sid., unk.	81.5	1.44 (0.01)		13.26 (0.12)		-2.97 (0.03)	**0.2**	**0.78**	**84**	**95**	

[a]Parameter uncertainties are presented for the last significant figures. Bold values represent fixed values during fitting.;
[b]Center shift;
[c]Quadrupole splitting (QS, for doublets) or Quadrupole shift (ε, for sextet);
[d]Hyperfine field;
[e]Standard deviation of QS (doublets) or H (sextet);
[f]Quadrupole splitting in paramagnetic state or quadrupole shift;
[g]Asymmetry parameter;
[h]Half line width at half maximum;
[i]Azimuthal angle between the electric field gradient axis of symmetry with hyperfine field H;
[j]Polar angle between the electric field gradient axis of symmetry with hyperfine field H;
[k]Goodness of fit;
Abbreviations: Temp.= Temperature; Fe(III) D = Fe(III) doublet; Fe(II) D1/D2= Fe(II) doublet; Fe(III) S1= Fe(III) sextet; min. = minerals; pyr. = pyrite; Fh = ferrihydrite; Lp = lepidocrocite; SRO = short ranged ordered; unk. = unknown, not clearly identifiable; Gt = (nano)goethite; GR = green rust-like phase; FeS_x = iron sulfide minerals such as mackinawite and greigite; Sid. = siderite; Viv. = vivianite.

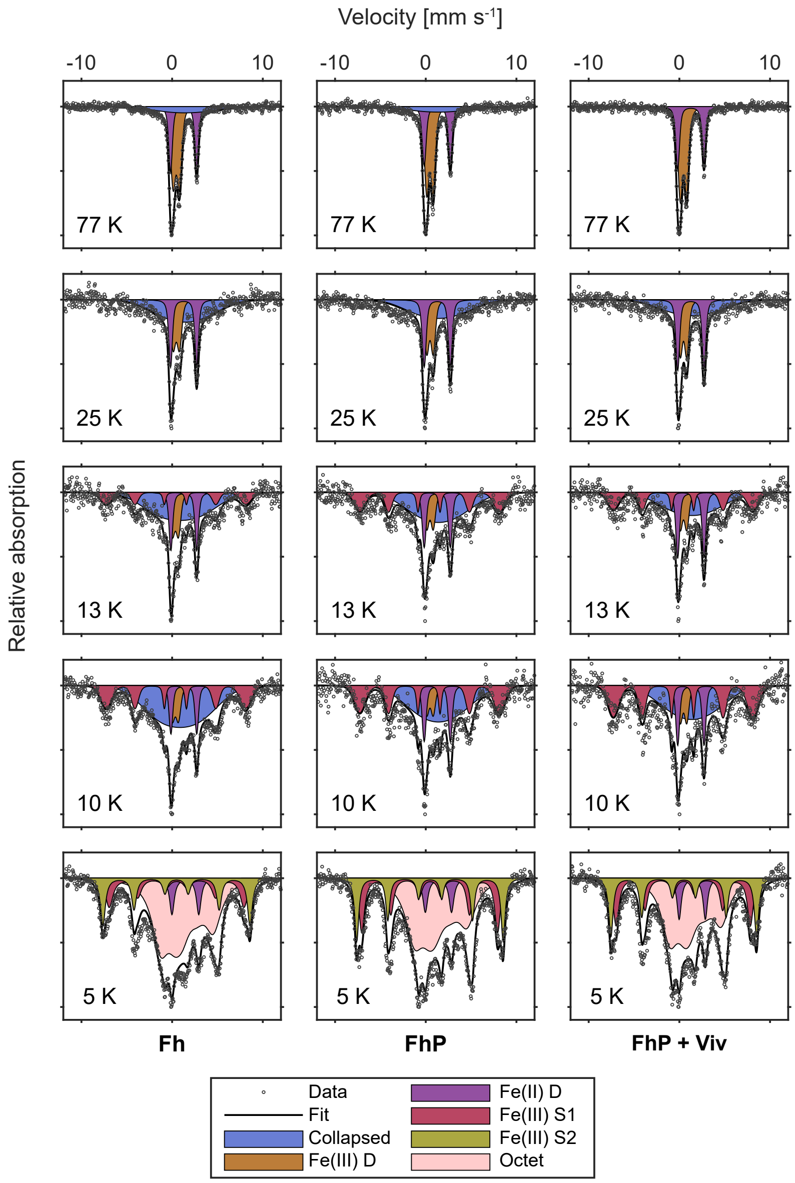

Figure S20 – Fitted Mössbauer spectra of reacted FKS samples. Note, that the fitted octet is oversimplified and likely contains multiple collapsed octets as discussed in Section S4.2. Fitted hyperfine parameters are presented in Tables S11-S13. Interpretation of fitted components: Fe(III) D = doublet of solid-phase Fe(III) likely present as Fe-oxides and/or green rust; Fe(II) D = doublet of solid-phase Fe(II) likely present in green rust, clay minerals and/or adsorbed Fe(II); Fe(III) S1 = sextet of solid-phase Fe(III) likely present in (short-ranged) Fe-oxide minerals such as ferrihydrite and lepidocrocite; Fe(III) S2 = sextet of solid-phase Fe(III) likely present in green rust and/or ferrihydrite; Octet = likely a mixture of iron minerals including Fe(II)-bound in green rust; Collapsed = depending on temperature of data collection and mineral specific Néel temperatures feature represents different mineral phases such as FeS_x, siderite, and Fe(III) in Fe-oxides or green rust.

Table S11 – Hyperfine parameters obtained for fitting of FKS Fh sample at 77 K, 25 K, 13 K and 10 K using xVBF model and 5 K using Full Static Hamiltonian model.[a]

**Temp.**	**Phase**	**Phase interpretation**	**Spectral Area %**	**CS**[b] **[mm s^{-1}]**	**QS**[c] **or ε [mm s^{-1}]**	**H**[d] **[T]**	**σ**[e] **[mm s^{-1}] or [T]**	**$e^2qQ/2$**[f] **[mm s^{-1}]**	**η**[g] **[mm s^{-1}]**	**w**[h] **[mm s^{-1}]**	**φ**[i] **[°]**	**ϑ**[j] **[°]**	**Red-χ^{2}**[k]
77 K	Fe(III) D	Fe(III) (SRO)oxides, GR. or Pyr.	48.7	0.46 (0.01)	0.69 (0.01)		**0.40**						*0.70*
	Fe(II) D1	Fe(II) in GR, sorbed, Fe(II) min.	33.3	1.27 (0.01)	2.88 (0.02)		**0.26**						
	Collapsed	FeS_x	18.0	0.42 (0.12)	**0.01**	14.32 (1.28)	**5.60**						
25 K	Fe(III) D	Fe(III) (SRO)oxides, GR. or Pyr.	22.4	**0.46**	**0.69**		**0.40**						*0.74*
	Fe(II) D1	Fe(II) in GR, sorbed, Fe(II) min.	25.5	**1.27**	**2.88**		**0.26**						
	Collapsed	Fe(II) in FeS_x, Sid., Fe(III) in oxides	52.1	**1.40**	**3.50**		**4.55**						
13 K	Fe(III) D	Fe(III) (SRO)oxides, GR. or Pyr.	14.2	0.36 (0.08)	0.67 (0.08)		**0.40**						*0.60*
	Fe(II) D1	Fe(II) in GR, sorbed, Fe(II) min.	16.1	1.29 (0.03)	2.85 (0.06)		**0.26**						
	Fe(III) S1	Fh-like, Lp-like	17.6	0.39 (0.11)	0.03 (0.11)	47.75 (0.97)	**3.54**						
	Collapsed	Fe(II) in FeS_x, Sid., Fe(III) in oxides	52.1	0.86 (0.26)	4.03 (0.58)		**4.55**						
10 K	Fe(III) D	Fe(III) (SRO)oxides, GR. or Pyr.	8.5	**0.36**	**0.67**		**0.40**						*0.65*
	Fe(II) D1	Fe(II) in GR, sorbed, Fe(II) min.	9.8	**1.29**	**2.85**		**0.26**						
	Fe(III) S1	Fh-like, Lp-like	24.5	**0.39**	**0.03**	**47.75**	**3.54**						
	Collapsed	Fe(II) in FeS_x, Sid., Fe(III) in oxides	57.2	**0.86**	**4.03**		**4.55**						
5 K	Fe(II) D1	Fe(II) sorbed, GR - unordered	6.3	1.46 (0.03)				2.96 (0.06)	**0**	**0.31**	**0**	**0**	*1.79*
	Fe(III) S1	Fh-like, Lp-like	16.8	0.50 (0.04)		46.08 (0.33)		-0.01 (0.00)	**0**	**0.51**	**0**	**0**	
	Fe(III) S2	Fh-like, Fe(III) in GR.	17.3	0.48 (0.02)		50.26 (0.17)		-0.02 (0.00)	**0**	**0.32**	**0**	**0**	
	Octet	Fe(II) in GR., FeS_x, Sid., unk.	59.6	1.17 (0.04)		13.17 (0.34)		-2.97 (0.09)	**0.2**	**1.10**	**84**	**95**	

[a]Parameter uncertainties are presented for the last significant figures. Bold values represent fixed values during fitting.;

[b]Center shift;

[c]Quadrupole splitting (QS, for doublets) or Quadrupole shift (ε, for sextet);

[d]Hyperfine field;

[e]Standard deviation of QS (doublets) or H (sextet);

[f]Quadrupole splitting in paramagnetic state or quadrupole shift;

[g]Asymmetry parameter;

[h]Half line width at half maximum;

[i]Azimuthal angle between the electric field gradient axis of symmetry with hyperfine field H;

[j]Polar angle between the electric field gradient axis of symmetry with hyperfine field H;

[k]Goodness of fit;

Abbreviations: Temp.= Temperature; Fe(III) D = Fe(III) doublet; Fe(II) D1= Fe(II) doublet; Fe(III) S1= Fe(III) sextet; min. = minerals; pyr. = pyrite; Fh = ferrihydrite; Lp = lepidocrocite; SRO = short ranged ordered; unk. = unknown, not clearly identifiable; Gt = (nano)goethite; GR = green rust-like phase; FeS_x = iron sulfide minerals such as mackinawite and greigite; Sid. = siderite; Viv. = vivianite.

Table S12 – Hyperfine parameters obtained for fitting of FKS FhP sample at 77 K, 25 K, 13 K and 10 K using xVBF model and 5 K using Full Static Hamiltonian model.[a]

**Temp.**	**Phase**	**Phase interpretation**	**Spectral Area %**	**CS[b] [mm s^{-1}]**	**QS[c] or ε [mm s^{-1}]**	**H[d] [T]**	**σ[e] [mm s^{-1}] or [T]**	**$e^2qQ/2$[f] [mm s^{-1}]**	**η[g] [mm s^{-1}]**	**w[h] [mm s^{-1}]**	**φ[i] [°]**	**ϑ[j] [°]**	**Red-$\chi^{2,k}$**
77 K	Fe(III) D	Fe(III) (SRO)oxides, GR. or Pyr.	52.1	0.47 (0.01)	0.71 (0.02)		**0.37**						*0.67*
	Fe(II) D1	Fe(II) in GR, sorbed, Fe(II) min.	32.4	1.27 (0.01)	2.88 (0.02)		**0.30**						
	Collapsed	FeS_x	15.5	0.34 (0.17)	**0.05**	16.2 (1.73)	**5.6**						
25 K	Fe(III) D	Fe(III) (SRO)oxides, GR. or Pyr.	24.7	**0.47**	**0.71**		**0.37**						*0.70*
	Fe(II) D1	Fe(II) in GR, sorbed, Fe(II) min.	29.0	**1.27**	**2.88**		**0.30**						
	Collapsed	Fe(II) in FeS_x, Sid., Fe(III) in oxides	46.3	**1.40**	**3.50**		**4.55**						
13 K	Fe(III) D	Fe(III) (SRO)oxides, GR. or Pyr.	10.8	**0.47**	**0.71**		**0.37**						*0.61*
	Fe(II) D1	Fe(II) in GR, sorbed, Fe(II) min.	15.2	**1.27**	**2.88**		**0.30**						
	Fe(III) S1	Fh-like, Lp-like	26.5	**0.39**	**0.03**	**47.75**	**3.54**						
	Collapsed	Fe(II) in FeS_x, Sid., Fe(III) in oxides	47.6	**1.40**	**3.50**		**4.55**						
10 K	Fe(III) D	Fe(III) (SRO)oxides, GR. or Pyr.	7.3	**0.47**	**0.71**		**0.37**						*0.66*
	Fe(II) D1	Fe(II) in GR, sorbed, Fe(II) min.	12.9	**1.27**	**2.88**		**0.30**						
	Fe(III) S1	Fh-like, Lp-like	31.5	**0.39**	**0.03**	**47.75**	**3.54**						
	Collapsed	Fe(II) in FeS_x, Sid., Fe(III) in oxides	48.3	**1.40**	**3.50**		**4.55**						
5 K	Fe(II) D1	Fe(II) sorbed, GR - unordered	5.9	1.40 (0.04)				2.89 (0.08)	**0**	**0.32**	**0**	**0**	*1.17*
	Fe(III) S1	Fh-like, Lp-like	22.0	0.47 (0.02)		46.46 (0.18)		-0.05 (0.05)	**0**	**0.37**	**0**	**0**	
	Fe(III) S2	Fh-like, Fe(III) in GR.	18.5	0.48 (0.03)		50.05 (0.13)		-0.03 (0.00)	**0**	**0.27**	**0**	**0**	
	Octet	Fe(II) in GR., FeS_x, Sid., unk.	53.6	1.19 (0.06)		13.44 (0.44)		-2.87 (0.13)	**0.2**	**1.17**	**84**	**95**	

[a]Parameter uncertainties are presented for the last significant figures. Bold values represent fixed values during fitting.;
[b]Center shift;
[c]Quadrupole splitting (Qs, for doublets) or Quadrupole shift (ε, for sextet);
[d]Hyperfine field;
[e]Standard deviation of QS (doublets) or H (sextet);
[f]Quadrupole splitting in the paramagnetic state or quadrupole shift;
[g]Asymmetry parameter;
[h]Half line width at half maximum;
[i]Azimuthal angle between the electric field gradient axis of symmetry with hyperfine field H;
[j]Polar angle between the electric field gradient axis of symmetry with hyperfine field H;
[k]Goodness of fit;
Abbreviations: Temp.= Temperature; Fe(III) D = Fe(III) doublet; Fe(II) D1= Fe(II) doublet; Fe(III) S1= Fe(III) sextet; min. = minerals; pyr. = pyrite; Fh = ferrihydrite; Lp = lepidocrocite; SRO = short ranged ordered; unk. = unknown, not clearly identifiable; Gt = (nano)goethite; GR = green rust-like phase; FeS_x = iron sulfide minerals such as mackinawite and greigite; Sid. = siderite; Viv. = vivianite.

Table S13 – Hyperfine parameters obtained for fitting of FKS FhP+Viv sample at 77 K, 25 K, 13 K and 10 K using xVBF model and 5 K using Full Static Hamiltonian model.[a]

**Temp.**	**Phase**	**Phase interpretation**	**Spectral Area %**	**CS[b] [mm s^{-1}]**	**QS[c] or ε [mm s^{-1}]**	**H[d] [T]**	**σ[e] [mm s^{-1}] or [T]**	**$e^2qQ/2$[f] [mm s^{-1}]**	**η[g] [mm s^{-1}]**	**w[h] [mm s^{-1}]**	**φ[i] [°]**	**ϑ[j] [°]**	**Red-$\chi^{2,k}$**
77 K	Fe(III) D	Fe(III) (SRO)oxides, GR. or Pyr.	61.9	0.46 (0.01)	0.71 (0.01)		0.36 (0.02)						*0.62*
	Fe(II) D1	Fe(II) in GR, sorbed, Fe(II) min.	38.1	1.27 (0.01)	2.90 (0.02)		0.28 (0.02)						
25 K	Fe(III) D	Fe(III) (SRO)oxides, GR. or Pyr.	27.0	**0.46**	**0.71**		**0.36**						*0.59*
	Fe(II) D1	Fe(II) in GR, sorbed, Fe(II) min.	31.1	**1.27**	**2.90**		**0.28**						
	Collapsed	Fe(II) in FeS_x, Sid., Fe(III) in oxides	41.9	**1.40**	**3.50**		**4.55**						
13 K	Fe(III) D	Fe(III) (SRO)oxides, GR. or Pyr.	12.2	**0.46**	**0.71**		**0.36**						*0.60*
	Fe(II) D1	Fe(II) in GR, sorbed, Fe(II) min.	19.4	**1.27**	**2.90**		**0.28**						
	Fe(III) S1	Fh-like, Lp-like	27.3	**0.39**	**0.03**	**47.75**	**3.54**						
	Collapsed	Fe(II) in FeS_x, Sid., Fe(III) in oxides	41.1	**1.40**	**3.50**		**4.55**						
10 K	Fe(III) D	Fe(III) (SRO)oxides, GR. or Pyr.	8.7	**0.46**	**0.71**		**0.36**						*0.69*
	Fe(II) D1	Fe(II) in GR, sorbed, Fe(II) min.	12.9	**1.27**	**2.90**		**0.28**						
	Fe(III) S1	Fh-like, Lp-like	35.1	**0.39**	**0.03**	**47.75**	**3.54**						
	Collapsed	Fe(II) in FeS_x, Sid., Fe(III) in oxides	43.3	**1.40**	**3.50**		**4.55**						
5 K	Fe(II) D1	Fe(II) sorbed, GR - unordered	6.8	1.42 (0.02)				2.89 (0.04)	**0**	**0.29**	**0**	**0**	*1.04*
	Fe(III) S1	Fh-like, Lp-like	21.3	0.46 (0.01)		45.91 (0.24)		-0.07 (0.00)	**0**	**0.38**	**0**	**0**	
	Fe(III) S2	Fh-like, Fe(III) in GR.	19.8	0.49 (0.01)		49.81 (0.16)		-0.02 (0.00)	**0**	**0.29**	**0**	**0**	
	Octet	Fe(II) in GR., FeS_x, Sid., unk.	52.1	1.33 (0.03)		13.37 (0.31)		-2.73 (0.07)	**0.2**	**1.06**	**84**	**95**	

[a]Parameter uncertainties are presented for the last significant figures. Bold values represent fixed values during fitting.;

[b]Center shift;

[c]Quadrupole splitting (QS, for doublets) or Quadrupole shift (ε, for sextet);

[d]Hyperfine field;

[e]Standard deviation of QS (doublets) or H (sextet);

[f]Quadrupole splitting in paramagnetic state or quadrupole shift;

[g]Asymmetry parameter;

[h]Half line width at half maximum;

[i]Azimuthal angle between the electric field gradient axis of symmetry with hyperfine field H;

[j]Polar angle between the electric field gradient axis of symmetry with hyperfine field H;

[k]Goodness of fit;

Abbreviations: Temp.= Temperature; Fe(III) D = Fe(III) doublet; Fe(II) D1= Fe(II) doublet; Fe(III) S1= Fe(III) sextet; min. = minerals; pyr. = pyrite; Fh = ferrihydrite; Lp = lepidocrocite; SRO = short ranged ordered; unk. = unknown, not clearly identifiable; Gt = (nano)goethite; GR = green rust-like phase; FeS_x = iron sulfide minerals such as mackinawite and greigite; Sid. = siderite; Viv. = vivianite.

S4.2.2 Overlay of reacted samples with vivianite and siderite references

We overlay the collected and normalized 5 K Mössbauer spectra (Fig. S21-S23) with reference spectra of vivianite and siderite. Both minerals exhibit multiple octets at 5 K, making fitting extremely challenging. Spectra of reacted HSF and HW clearly showed consistency with siderite and vivianite, while features of vivianite were missing in FKS spectra. Features of vivianite were more clearly identifiable in reacted FhP and FhP+Viv samples from HSF and HW, aligning with the ability to quantity the vivianite pool in FhP and FhP+Viv samples. Some features of vivianite were also visible in Fh samples at HSF and HW, such as a slight shoulder feature at about 6 mm s^{-1}, which may indicate the presence of small amounts of vivianite that fell below the quantification limit.

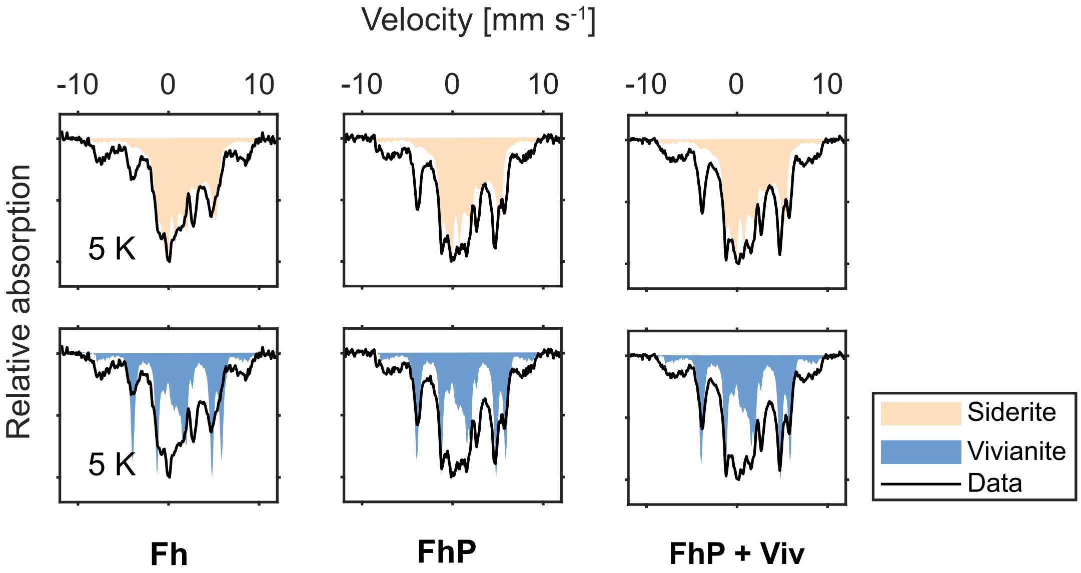

Figure S21 – Overlay of HSF Mössbauer data for the three treatments with siderite at 5 K (top), as well as vivianite at 5 K (bottom).

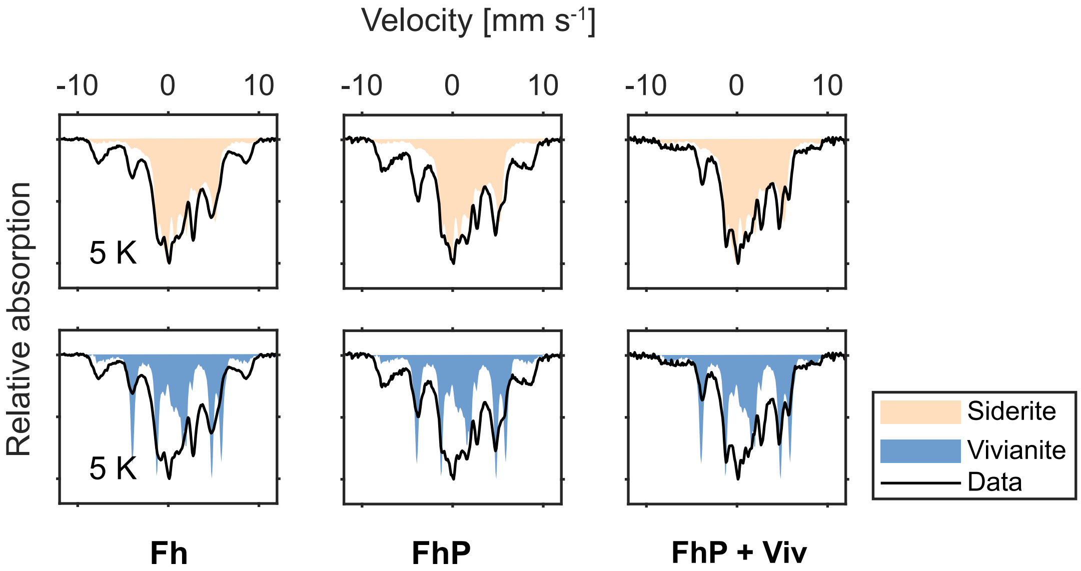

Figure S22 – Overlay of HW Mössbauer data for the three treatments with siderite at 5 K (top), as well as vivianite at 5 K (bottom).

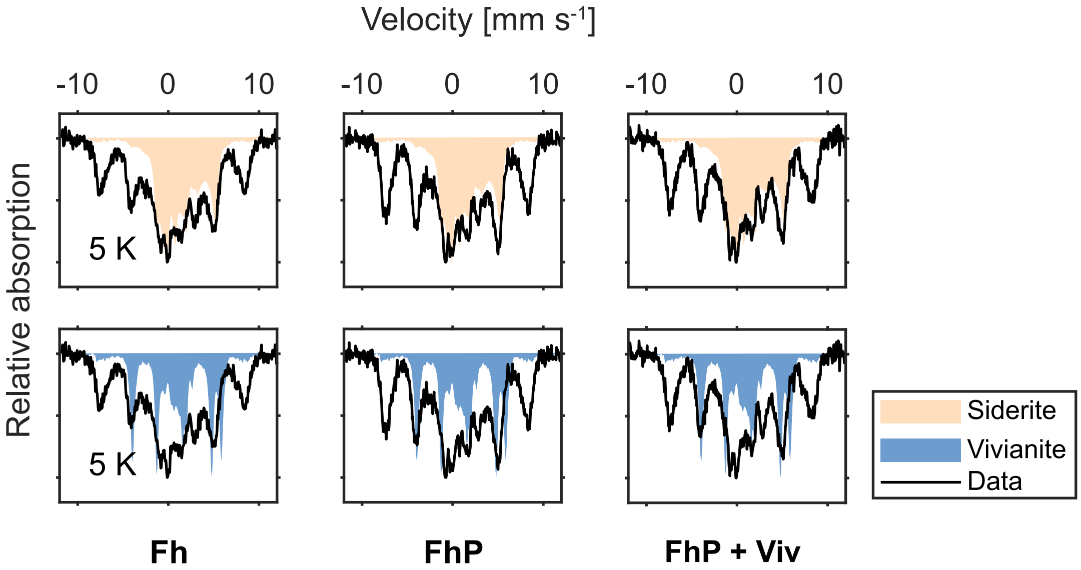

Figure S23 – Overlay of FKS Mössbauer data for the three treatments with siderite at 5 K (top), as well as vivianite at 5 K (bottom).

S4.2.3 Identifying the presence of siderite based on Fe(II) temperature trends

The Néel temperature of siderite is $\sim$ 37 K.[7] Consequently, at 77 K Fe(II) bound in siderite is present as a doublet, while from $\sim$ 37 K Fe(II) starts to undergo magnetic ordering, forming an octet. In comparison to other Fe(II) minerals such as green rust and vivianite, which only start to magnetically order below 12 K,[22,31] siderite has a relatively high Néel temperature. We used this difference to test if potentially some siderite was present in our reacted samples. If siderite was present in our samples, the sum of the spectral area of Fe(II)D1 and Fe(II)D2 should decrease from 77 K to 25 K due to the magnetic ordering of siderite. In case of the absence of siderite, the sum of the spectral area of Fe(II)D1 and Fe(II)D2 should be the same at 77 K and 25 K. In Figure S24, we plotted the spectral area of Fe(II) doublet(s) against temperature. Based on this profile, our results show that siderite was likely present in reacted Fh samples across all field sites. Additionally, potentially, there were some minor amounts of siderite in the FhP+Viv sample incubated at HW and in the FhP(+Viv) samples of FKS.

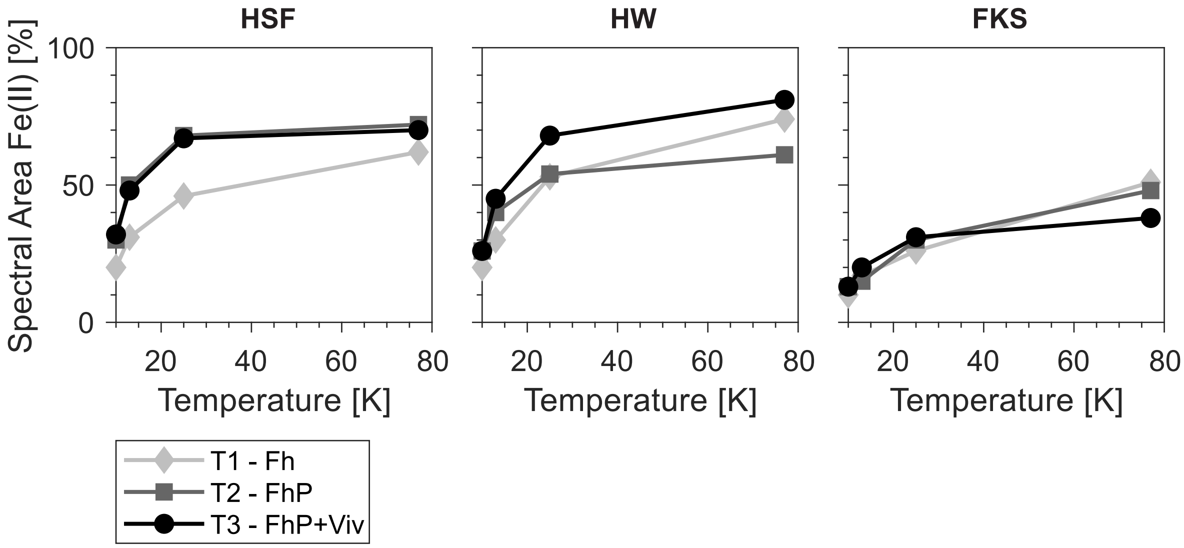

Figure S24 – Fitted spectral area of ferrous iron (Fe(II) D1 and D2) of Mössbauer spectra at different temperatures for the three field sites and three field treatments.

S4.2.4 Mössbauer data of initial and reacted unamended sediment

Mössbauer spectra of the initial and reacted unamended sediment (control) were collected for each field site. The initial unamended sediment was collected and dried under oxic conditions, while the reacted unamended sediment was collected, and dried under oxic conditions followed by an incubation in the field, and subsequent drying under anoxic conditions. Most of the Fe (68 to 75%, Table S14) was present as Fe(III) in the initial sediment across all field sites. The Fe(III) was likely present in clay minerals and Fe-oxide minerals with varying crystallinity, ranging from short-ranged Fe-oxide minerals to lepidocrocite based on fitted hyperfine parameters, which is consistent with the results of the sequential Fe extraction. Due to spectral noise, it was impossible to unambiguously identify the present Fe(III)-mineral phases. The present Fe(II) was likely bound in clay minerals or adsorbed Fe(II).

During the field incubation, Fe(III) was reduced and its contribution ranged between 44 to 58% (Table S15). Interestingly, 5 K Mössbauer spectra of all field sites contained a collapsed feature with a large spectral area ($\sim$ 50%). This collapsed feature indicates the presence of highly disordered Fe-mineral phases, which could include Fe(III) and Fe(II) complexed by organic material,[38] green rust phases which have not been magnetically ordered[31] or disordered Fe-sulfide minerals.[34] Due to high spectral noise, the collapsed feature might hide the presence of octets which could indicate the presence of green rust, siderite, and/or vivianite. In contrast, to the ^{57}Fe-amended treatments, an Fe(III)-sextet (Fe(III)S1) emerged at 5 K at HSF and HW with hyperfine parameters consistent with (nano)goethite (Table S15)[24] and aligning with Fe K-edge EXAFS results (Section S4.4). The initial Mössbauer spectra of HSF and HW had an Fe(III)-sextet at 5 K, which had hyperfine parameters consistent with ferrihydrite or lepidocrocite.[37,24] Thus, the change in hyperfine parameters during the field incubation suggest that Fe(II)-catalyzed transformation to more crystalline Fe-oxide minerals occurred. This is frequently observed in Fe(II)-spiked laboratory studies investigating the transformation of ferrihydrite and lepidocrocite but was absent in our amended treatments. Consequently, this result indicates that adding ferrihydrite to natural sediment somewhat altered the transformation pathway, and Fe(II)-catalyzed transformation might have still occurred. However, its contribution was likely hidden by the extensive reductive transformation that occurred in the amended, as well as unamended samples.

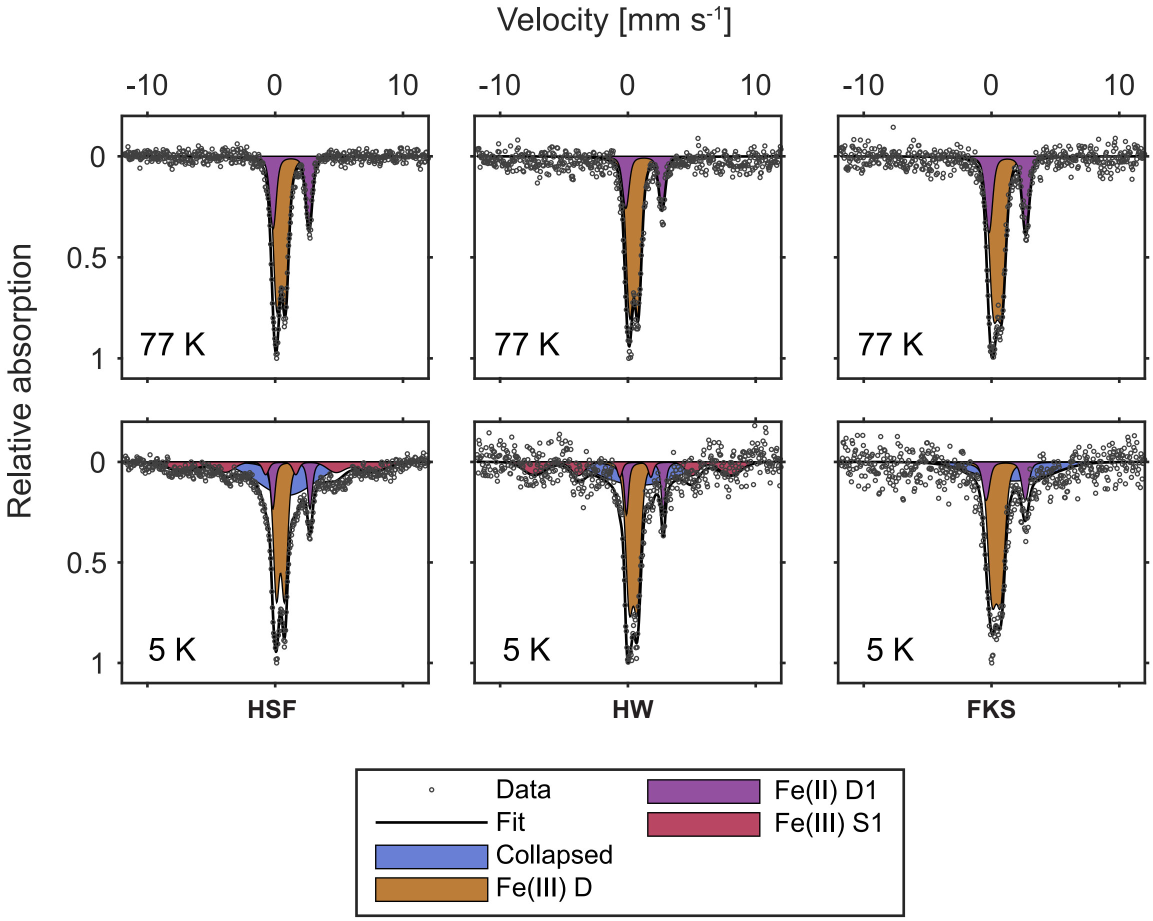

Figure S25 – Fitted Mössbauer spectra of the initial sediment from the three field sites. The sediment was dried under an ambient atmosphere at 30°C.

Table S14 – Hyperfine parameters obtained for fitting of initial sediment. Mössbauer spectra were collected at 77 K and 5 K.[a]

**Sample**	**Temp.**	**Phase**	**Phase interpretation**	**Spectral Area %**	**CS[b] [mm s^{-1}]**	**QS[c] or ε [mm s^{-1}]**	**H[d] [T]**	**σ[e] [mm s^{-1}] or [T]**	**Red χ^{2,f}**
**HSF**	77 K	Fe(III) D	Fe(III) in oxides, clays or Pyr.	67.3	0.46 (0.01)	0.67 (0.01)		0.40 (0.02)	**0.76**
		Fe(II) D1	Fe(II) in clays sorbed, Fe(II) min.	32.7	1.24 (0.02)	2.82 (0.03)		0.36 (0.04)	
	5 K	Fe(III) D	Pyr., clays	34.2	0.42 (0.01)	0.64 (0.02)		0.35	**0.68**
		Fe(II) D1	Sorbed Fe(II), Fe(II) not ordered	10.3	1.27 (0.02)	2.91 (0.04)		0.22	
		Fe(III) S1	Fh-like, Lp-like	20.5	0.31 (0.12)	-0.12 (0.11)	46.20 (1.1)	6.72	
		Collapsed	Very SRO-Fe-oxides; unk.	34.8	0.89 (0.14)	-0.01		4.37	
**HW**	77 K	Fe(III) D	Fe(III) in oxides, clays or Pyr.	74.9	0.47 (0.02)	0.68 (0.02)		0.42	**0.63**
		Fe(II) D1	Fe(II) in clays sorbed, Fe(II) min.	25.1	1.26 (0.03)	2.84 (0.07)		0.37	
	5 K	Fe(III) D	Pyr., clays	39.6	0.42 (0.04)	0.63 (0.05)		0.43	**0.49**
		Fe(II) D1	Sorbed Fe(II), Fe(II) not ordered	10.1	1.33 (0.05)	2.88 (0.10)		0.17	
		Fe(III) S1	Fh-like, Lp-like	20.4	0.47 (0.21)	-0.11 (0.21)	47.70 (1.90)	4.84	
		Collapsed	Very SRO-Fe-oxides; unk.	29.8	0.93 (0.57)	3.20 (1.50)		4.10	
**FKS**	77 K	Fe(III) D	Fe(III) in oxides, clays or Pyr.	67.8	0.47 (0.02)	0.67 (0.03)		0.52	**0.69**
		Fe(II) D1	Fe(II) in clays sorbed, Fe(II) min.	32.2	1.25 (0.03)	2.88 (0.05)		0.41	
	5 K	Fe(III) D	Pyr., clays	54.3	0.38 (0.05)	0.70 (0.05)		0.52	**0.52**
		Fe(II) D1	Sorbed Fe(II), Fe(II) not ordered	14.4	1.11 (0.09)	3.06 (0.17)		0.41	
		Collapsed	Very SRO-Fe-oxides; unk.	31.4	1.67 (0.71)	3.20 (1.50)		4.00	

[a]Parameter uncertainties are present for the last significant figures. Parameters without uncertainty were fixed;

[b]Center shift;

[c]Quadrupole splitting (QS, for doublets) or Quadrupole shift (ε, for sextet);

[d]Hyperfine field;

[e]Standard deviation of QS (doublets) or H (sextet);

[f]Goodness of fit;

Abbreviations: Temp.= Temperature; Fe(III) D = Fe(III) doublet; Fe(II) D1= Fe(II) doublet; Fe(III) S1= Fe(III) sextet.; min. = minerals; pyr. = pyrite; Fh = ferrihydrite; Lp = lepidocrocite; SRO = short ranged ordered; unk. = unknown, not clearly identifiable; Gt = (nano)goethite

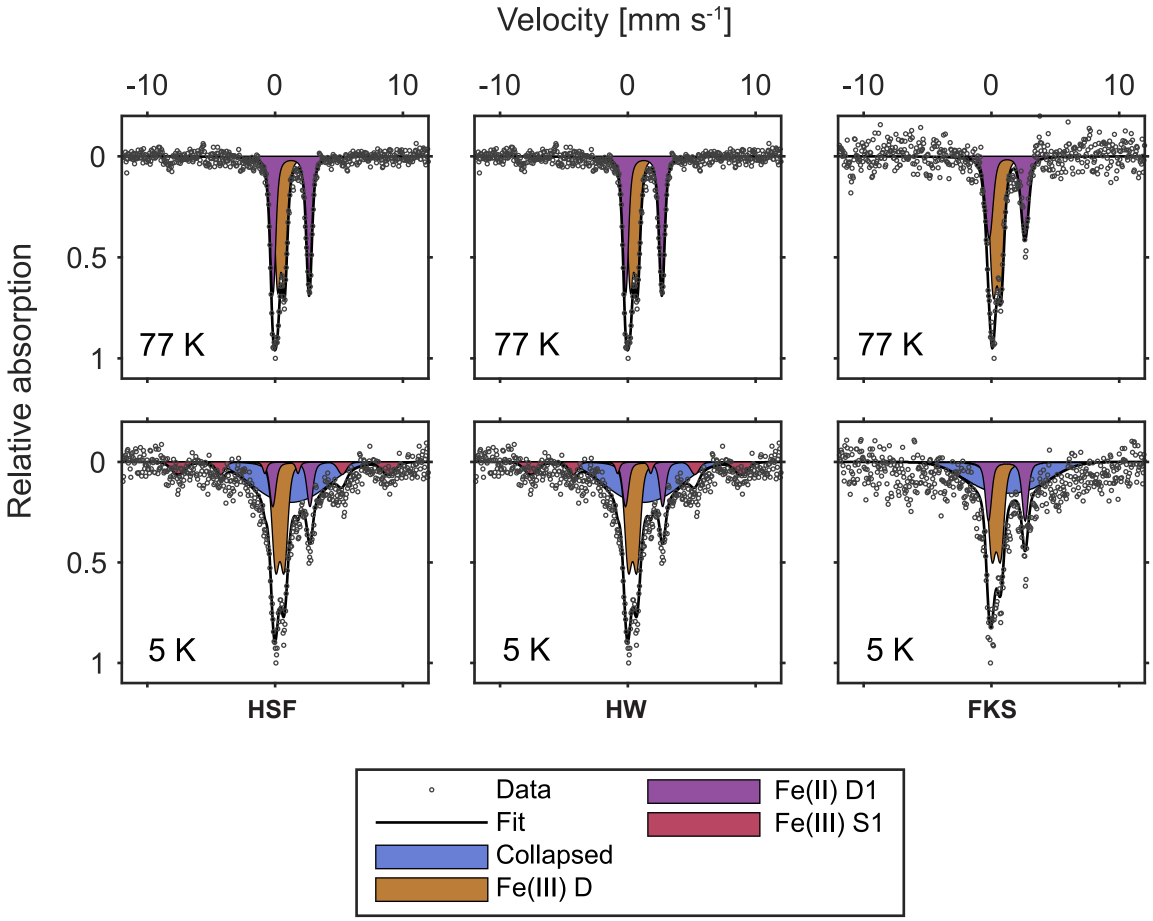

Figure S26 – Fitted Mössbauer spectra of the reacted sediments (Controls) from the three field sites.

Table S15 – Hyperfine parameters obtained for fitting of reacted sediment (Control). Mössbauer spectra were collected at 77 K and 5 K.[a]

**Sample**	**Temp.**	**Phase**	**Phase interpretation**	**Spectral Area %**	**CS[b] [mm s^{-1}]**	**QS[c] or ε [mm s^{-1}]**	**H[d] [T]**	**σ[e] [mm s^{-1}] or [T]**	**Red χ[2,f]**
**HSF**	77 K	Fe(III) D	Fe(III) in oxides, clays, GR. or Pyr.	44.3	0.47 (0.01)	0.54 (0.01)		0.27	**0.79**
		Fe(II) D1	Fe(II) in clays sorbed, Fe(II) min.	55.7	1.25 (0.01)	2.86 (0.01)		0.29	
	5 K	Fe(III) D	Pyr., clays	24.8	0.40 (0.02)	0.66 (0.02)		0.34	**0.78**
		Fe(II) D1	Sorbed Fe(II), Fe(II) not ordered	13.7	1.26 (0.02)	2.92 (0.03)		0.21	
		Fe(III) S1	Gt-like[g]	12.3	0.34 (0.10)	-0.10 (0.10)	50.13 (0.91)	3.67	
		Collapsed	Very SRO-oxides, GR, FeS_x,unk.	49.2	1.05 (0.15)	3.09 (0.28)		3.67	
**HW**	77 K	Fe(III) D	Fe(III) in oxides, clays, GR. or Pyr.	47.2	0.46 (0.01)	0.55 (0.02)		0.30 (0.03)	**0.65**
		Fe(II) D1	Fe(II) in clays sorbed, Fe(II) min.	52.8	1.25 (0.01)	2.83 (0.02)		0.30 (0.02)	
	5 K	Fe(III) D	Pyr., clays	28.7	0.37 (0.04)	0.67 (0.05)		0.43	**0.58**
		Fe(II) D1	Sorbed Fe(II), Fe(II) not ordered	11.3	1.27 (0.06)	2.91 (0.11)		0.34	
		Fe(III) S1	Gt-like[g]	12.6	0.57 (0.13)	0.07 (0.13)	50.95 (1.19)	2.89	
		Collapsed	Very SRO-oxides, GR, FeS_x,unk.	47.4	1.18 (0.24)	3.06 (0.55)		3.78	
**FKS**	77 K	Fe(III) D	Fe(III) in oxides, clays, GR. or Pyr.	57.9	0.44 (0.03)	0.61 (0.05)		0.39 (0.08)	**0.54**
		Fe(II) D1	Fe(II) in clays sorbed, Fe(II) min.	42.1	1.22 (0.04)	2.80 (0.09)		0.46 (0.11)	
	5 K	Fe(III) D	Pyr., clays	32.4	0.38 (0.06)	0.67 (0.07)		0.43	**0.62**
		Fe(II) D1	Sorbed Fe(II), Fe(II) not ordered	18.7	1.20 (0.06)	2.87 (0.11)		0.34	
		Collapsed	Very SRO-oxides, GR, FeS_x,unk.	48.9	1.25 (0.43)	3.34 (0.92)		3.78	

[a]Parameter uncertainties are present for the last significant figures. Parameters without uncertainty were fixed;

[b]Center shift;

[c]Quadrupole splitting (QS, for doublets) or Quadrupole shift (ε, for sextet);

[d]Hyperfine field;

[e]Standard deviation of QS (doublets) or H (sextet);

[f]Goodness of fit;

[g] Present Gt likely very poorly crystalline as not magnetically ordered at 77 K;

Abbreviations: Temp.= Temperature; Fe(III) D = Fe(III) doublet; Fe(II) D1= Fe(II) doublet; Fe(III) S1= Fe(III) sextet; min. = minerals; pyr. = pyrite; Fh = ferrihydrite; Lp = lepidocrocite; SRO = short ranged ordered; unk. = unknown, not clearly identifiable; Gt = (nano)goethite; GR = green rust-like phase; FeS_x = iron sulfide minerals such as mackinawite and greigite

S4.3 PCA analysis for LCF of Fe K-edge EXAFS specra

To constrain the suitable number of references used for linear combination fitting (LCF) of Fe K-edge extended X-ray absorption fine structure (EXAFS) spectra, principal component analysis (PCA) was employed using SixPack.[43] The E_0 of all spectra was set to 7128 eV, and the PCA analysis was performed on k^3-weighted EXAFS spectra over 2-10.5 Å^{-1} (n=16). PCA of Fe K-edge EXAFS spectra indicated that a maximum of four statistically significant spectral components were required to account for 95% of spectral variance (Table S16). Following PCA, target-transforming testing (TT, 4 components) was undertaken in SixPack.

Table S16 – PCA output parameters for Fe (n=18).

**Component**	**Eigenvalue**	**Cum Variance**	**IND**
1	85.394	0.822	0.00781
2	7.278	0.892	0.00567
3	5.238	0.943	0.00289
4	1.837	0.960	0.00235
5	1.317	0.973	0.00183
6	0.715	0.980	0.00172
7	0.575	0.986	0.00159
8	0.449	0.990	0.00143
9	0.326	0.993	0.00129
10	0.246	0.995	0.00109
11	0.138	0.997	0.00108
12	0.115	0.998	0.00089
13	0.063	0.999	0.00086
14	0.030	0.999	0.00116
15	0.026	0.999	0.00167

Table S17 – Result from Fe target-transform testing of used references. Abbreviation: NSSR = Normalized sum of squared residuals ($\sum_i (data_i - fit_i)^2 / \sum_i data^2$).

**Reference**	χ^2	**NSSR (%)**	**SPOIL**
Ferro-smectite	46.84	3.4	1.56
Biotite	193.18	14.2	1.27
Illite	42.6	3.5	2.18
Pyrite	1063	20.5	1.07
Mackinawite	297.4	21.7	1.63
Siderite	837.6	50.7	2.04
Vivianite	344.8	24.0	1.30
Green rust	358.7	24.2	2.25
Goethite	240.2	14.6	1.17
Amorphous ferric phosphate	173.8	14.0	1.56
Ferrihydrite	40	5.3	1.87

S4.4 LCF of Fe K-edge EXAFS spectra

Results of the LCF of Fe K-edge EXAFS spectra aligned with the sequential Fe extraction. About 48 to 76% of Fe in all samples and field sites were fitted as silicate minerals (illite, biotite, ferrosmectite, Table S18). Initial samples of the Fh treatment had 22 to 41 % of Fe fitted as Fh (Table S18, Fig. S28). Small amounts of goethite (8 to 12 %) were also fitted for initial samples of the Fh treatment of HSF and HW. While initial samples of the other two treatments were not measured, we assume that those had similar compositions as the increase in total Fe through the addition of Fh or FhP was consistent across the three different treatments (Table 1).

In reacted samples, LCF of Fe K-edge EXAFS spectra indicated no Fh, and fitted phases varied across all field sites and treatments. At HSF and HW, all reacted samples (treatments and unamended sediment) contained Fe fitted as green rust (10 to 14 % and 15 to 20%, respectively; Table S18, S19). The control sample (unamended reacted sediment) at HW contained goethite (14%), while siderite (8%) was suggested to be present in the reacted Fh sample at HW. Additionally, vivianite was present in FhP and FhP+Viv samples at HW (11 and 15%, respectively). In contrast to HSF and HW, the presence of green rust was not suggested in FKS samples by LCF of the Fe K-edge EXAFS spectra. Reacted Fh samples from FKS contained pyrite (17%) as well as ferric phosphate (22%), while only mackinawite was suggested to be present in FhP and FhP+Viv samples (14 and 9%, respectively, Table S18). Low data quality for the control sample (unamended reacted sediment) of FKS precluded LCF analysis (data not shown).

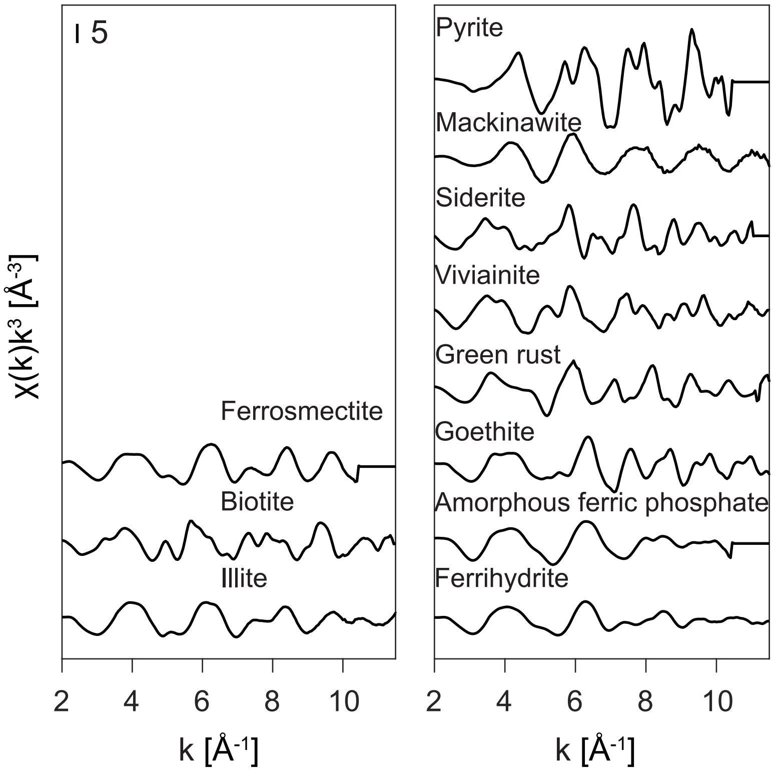

Figure S27 – k^3-weighted Fe K-edge EXAFS spectra of reference phases. Sources for Fe K-edge EXAFS spectra: ferro-smectite - courtesy of T. Borch (Colorado State University, USA); biotite, goethite, amorphous ferric phosphate, ferrihydrite, pyrite[21]; Illite[9]; siderite[17]; vivianite[18]; carbonate green rust[39]; mackinawite - courtesy of E. D. Burton (Southern Cross University, Australia).

Table S18 – Linear combination fit results of Fe K-edge EXAFS spectra. Fe(III)-clay was fitted using either ferro-smectite or illite, while Fe(II)-clay was fitted using biotite as a reference. ferro-smectite was used for FKS spectra, while illite was used for HW and HSF spectra. The illite used also contained some Fe(II). Abbreviations: Fh = ferrihydrite; Gt = goethite; Sid = siderite; GR = green rust; Viv = vivianite; Mkw = mackinawite; Py = pyrite; Fe(III)-P = amorphous ferric phosphate.

Site	Treatment	Fe(III)-clay [%]	Fe(II)-clay [%]	Fh [%]	Gt [%]	Sid [%]	GR [%]	Viv [%]	Mkw [%]	Py [%]	Fe(III)-P [%]	NSSR[a] [%]	Red. $\chi^{2,b}$
**HSF**	Fh initial	28	20	41	12							4.2	0.22
	Fh	42	31				11		16			4.7	0.25
	FhP	57	34				10					12.5	0.82
	FhP+Viv	38	48				14					26.1	1.92
**HW**	Fh initial	63	7	22	8							2.3	0.11
	Fh	37	33			8	22					5.6	0.24
	FhP	41	29				19	11				5.3	0.23
	FhP+Viv	43	28				15	15				5.1	0.23
**FKS**	Fh initial	60	16	24								2.9	0.18
	Fh	22	39							17	22	8.6	0.53
	FhP	55	26						14			3.2	0.15
	FhP+Viv	68	24						9			10.8	0.59

[a]Normalized sum of squared residuals ($\sum_i(\mathrm{data}_i - \mathrm{fit}_i)^2 / \sum_i \mathrm{data}^2$);
[b]Fit accuracy; reduced $\chi^2 = (N_{idp}/N_{pts}) \sum_i(\mathrm{data}_i - \mathrm{fit}_i)/\varepsilon_i)^2 (N_{idp} - N_{var})^{-1}$. N_{idp}, N_{pts}, and N_{var} are, respectively, the number of independent data points in the model fit (17 for HSF and FKS, 18.9 for HW), the total number of data points (169 for HSF and FKS, 189 for HW), and the number of fitted variables (2-4).

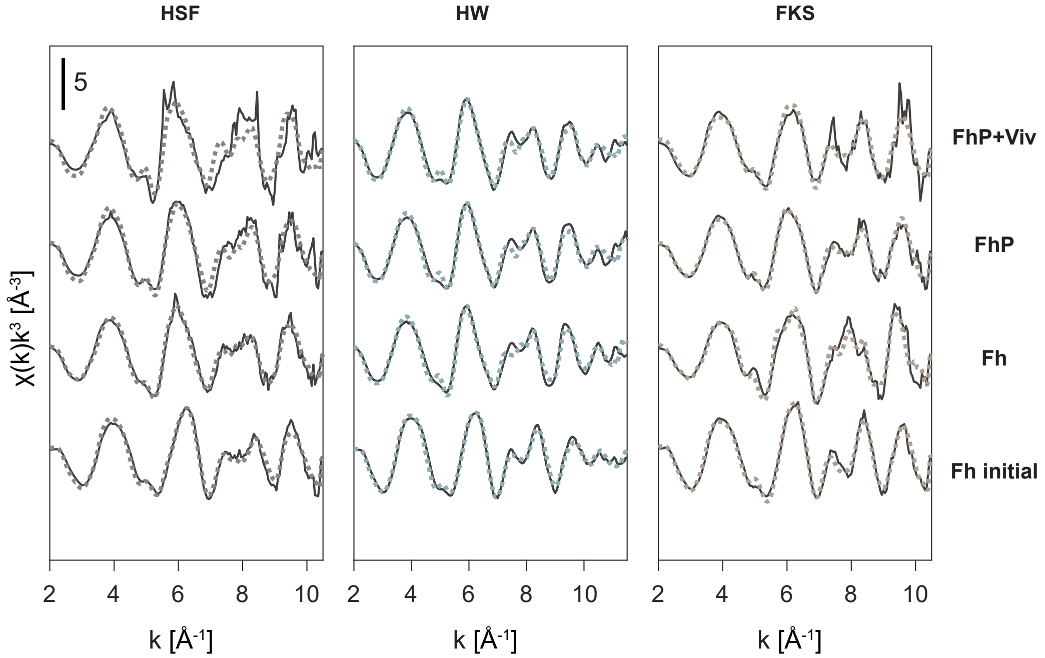

Figure S28 – k^3-weighted Fe K-edge EXAFS spectra of reacted samples. Solid lines indicate experimental data and dotted lines show the linear combination fit (LCF). Reference spectra can be found in Table S27. The corresponding values of the LCF are reported in Table S18. Abbreviations: HSF = Haseldorfer Marsch - low salinity site, HW = Hollerwettern - medium salinity site, FKS = Friedrichskoog - high salinity site.

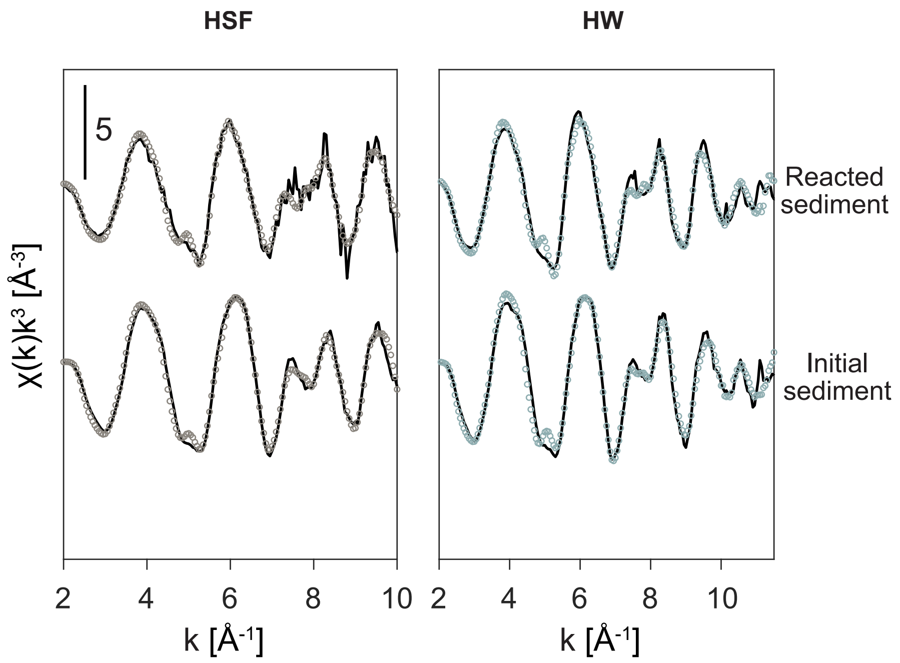

Figure S29 – k^3-weighted Fe K-edge EXAFS spectra of initial and reacted (Control) sediment. Solid lines indicate experimental data and dotted lines show the linear combination fit (LCF). Results of the LCF are presented in Table S19. Spectra of FKS are not shown presented nor fitted due to poor data quality.

Table S19 – Linear combination fit results of Fe K-edge EXAFS spectra of initial and reacted (Control) sediment. Fe K-edge EXAFS data of FKS could not be fitted due to poor data quality. Abbreviations: Gt = goethite; GR = green rust.; NSSR = normalized sum of squared residuals $(\sum_i(\text{data}_i - \text{fit}_i)^2/\sum_i \text{data}^2)$; Red. $\chi^2 = (N_{idp}/N_{pts})\sum_i(\text{data}_i - \text{fit}_i)/\varepsilon_i)^2(N_{idp} - N_{var})^{-1}$. N_{idp}, N_{pts}, and N_{var} are, respectively, the number of independent data points in the model fit (17 for HSF, 18.9 for HW), the total number of data points (169 for HSF, 189 for HW), and the number of fitted variables (3-4).

**Sample**	**Illite** [%]	**Biotite** [%]	**Gt** [%]	**GR** [%]	**NSSR** [%]	**Red.** χ^2
HSF_initial sediment	56	18	17	9	2.0	0.30
HSF_reacted sediment	49	34		17	10.2	0.45
HW_initial sediment	76	16	8		3.6	0.22
HW_reacted sediment	37	33	14	16	4.2	0.21

S4.5 LCF of Fe K-edge XANES spectra

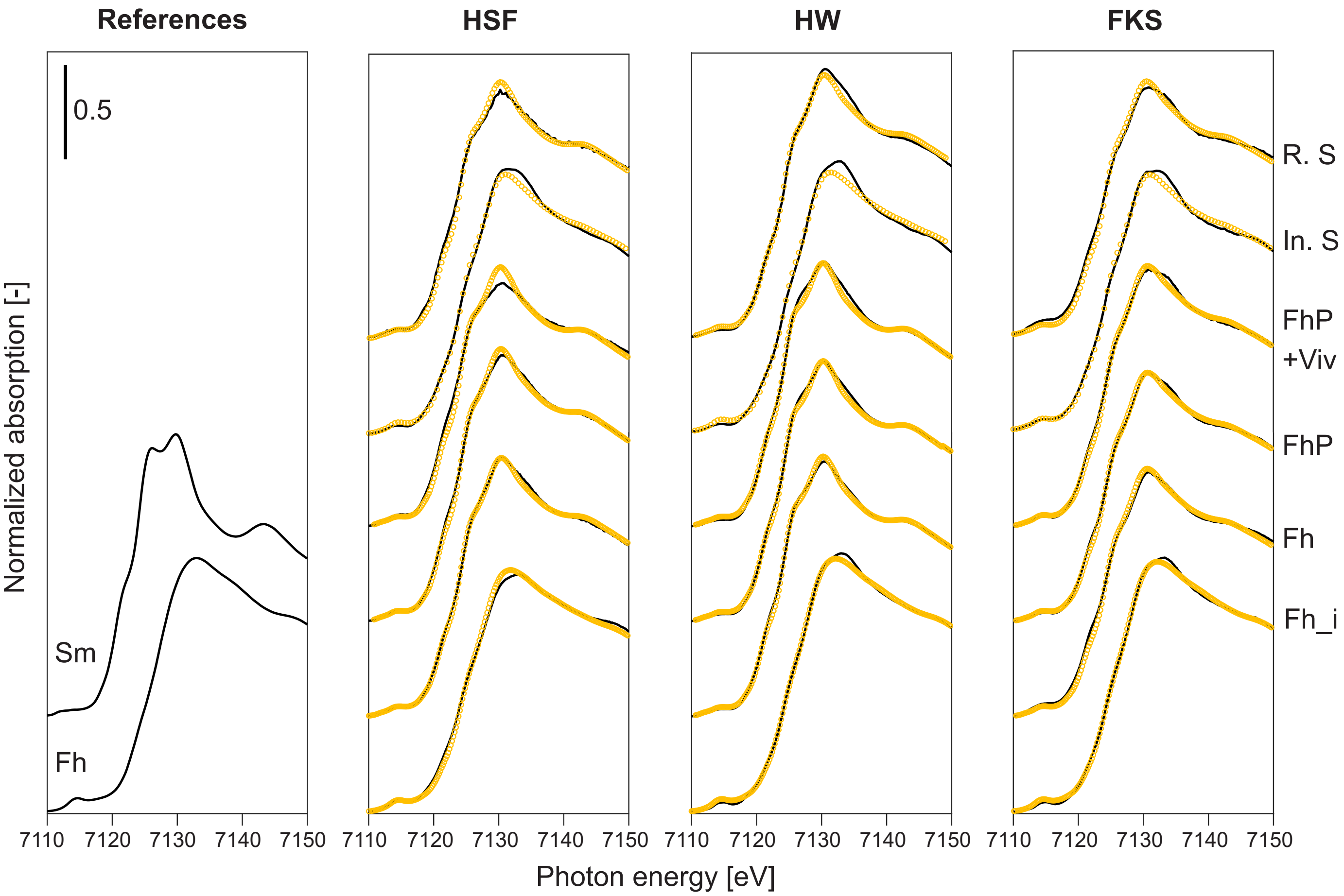

Figure S30 – Normalized Fe K-edge XANES spectra and their linear combination fits (LCF). Experimental data and LCF are shown in black and yellow, respectively. Abbreviations: Sm = reduced smectite, Fh = ferrihydrite, R. S. = reacted unamended sediment; In. S. = initial unamended sediment; FhP+Viv = reacted FhP+Viv treatment; FhP = reacted FhP treatment; Fh = reacted Fh treatment; HSF = Haseldorfer Marsch - low salinity site; HW = Hollerwettern - medium salinity site; FKS = Friedrichskoog - high salinity site.

Table S20 – Linear combination fit results for Fe K-edge XANES spectra. Fe(III) and Fe(II) were fit by the references ferrihydrite and reduced smectite, respectively. NSSR = normalized sum of squared residuals $(\sum_i (\mathrm{data}_i - \mathrm{fit}_i)^2 / \sum_i \mathrm{data}^2)$; Red. $\chi^2 = (N_{idp}/N_{pts}) \sum_i (\mathrm{data}_i - \mathrm{fit}_i)/\varepsilon_i)^2 (N_{idp} - N_{var})^{-1}$. N_{idp}, N_{pts}, and N_{var} are, respectively, the number of independent data points in the model fit (37.5), the total number of data points (250), and the number of fitted variables (2).

Sample		Fe(III)	Fe(II)	NSSR	Red. χ^2
		[%]			
HSF	Initial sediment	67	33	0.200	0.00054
	Reacted sediment	41	59	0.290	0.00064
	Fh initial	80	20	0.186	0.00043
	Fh reacted	49	51	0.066	0.00016
	FhP reacted	46	54	0.101	0.00026
	FhP+Viv reacted	41	59	0.413	0.00094
HW	Initial sediment	74	26	0.294	0.00082
	Reacted sediment	49	51	0.182	0.00046
	Fh initial	86	14	0.067	0.00018
	Fh reacted	40	60	0.063	0.00015
	FhP reacted	40	61	0.067	0.00016
	FhP+Viv reacted	38	62	0.126	0.00030
FKS	Initial sediment	67	33	0.151	0.00038
	Reacted sediment	54	46	0.286	0.00065
	Fh initial	86	14	0.060	0.00015
	Fh reacted	57	43	0.225	0.00049
	FhP reacted	59	41	0.076	0.00018
	FhP+Viv reacted	58	42	0.010	0.00025

S4.6 Ferrous fraction in solid phase

Mössbauer spectroscopy detects only ^{57}Fe, while Fe K-edge XAS measures the bulk Fe composition (sum of all Fe isotopes). Both techniques can be used to determine the samples' Fe(II) fraction. In natural sediment, in which no ^{57}Fe was added, the determined Fe(II) fraction of both techniques should be the same. This is consistent with our measurements (Table S21). Across all field sites, the Fe(II) fraction increased during the field incubation (compare initial with reacted sediment), indicating the reductive dissolution and transformation of present Fe-phases. These may range from Fe-oxide minerals to Fe-containing clay minerals. A reduction of clay minerals with incubation aligns with the observation made from LCF of the Fe K-edge EXAFS, which indicated an increase in the biotite (reduced clay mineral) fraction with incubation (Table S19).

The determined Fe(II) fraction in ^{57}Fe-amended samples differed between the two techniques. The initial Fh samples had no or minimal Fe(II) based on Mössbauer spectroscopy, as at least 95% of the spectra originated from the added ^{57}Fe in the form of ferrihydrite. After field incubation, the determined Fe(II) fraction based on Mössbauer spectroscopy was generally higher than the Fe(II) fraction determined by Fe K-edge XAS. This likely indicates a preferential reduction of the added ferrihydrite over natural Fe-mineral phases, which is not surprising as ferrihydrite is a highly reactive Fe-mineral.[35]

The degree of reductive transformation was interestingly similar across different treatments (Table S21), indicating that the presence of adsorbed P did not consistently impact reduction rates. The consistency in dissimilatory reduction extents despite the presence of adsorbed P contrasts with previous findings, where the presence of P either decreased[2] or enhanced[8,19] microbial-driven dissimilatory reduction rates. The impact of P on Fe reduction rates appears to depend on a range of variables, including the Fe:P ratio, the initial Fe mineral, the method of P addition (solution spike, adsorbed, co-precipitated), and the overall P availability for the microbial community.[8,2,13,16,25] Consequently, the results of different studies may not be directly comparable. Additionally, Ginn et al.[13] demonstrated that amending soils with P initially accelerated microbial-driven Fe(II) production, while the maximum Fe(II) produced was similar in amended and non-amended soils. Our experiment did not resolve temporal Fe(II) production rates; thus, such an effect might be hidden. Furthermore, the complex sediment matrix, including the presence of other ligands besides P, may also influence reduction rates. Therefore, P adsorption's impact on reduction rates might be masked, potentially explaining the absence of consistent differences in reductive dissolution in the presence or absence of adsorbed P.

Table S21 – Comparison of Fe(II) determined by Mössbauer spectroscopy and LCF of the Fe K-edge XANES region.

	**Sample**	**MB-Fe(II)** [%]	**XAS-Fe(II)** [%]
**HSF**	Initial sediment	33	33
	Reacted sediment	56	59
	Fh initial	2	20
	Fh reacted	62	51
	FhP reacted	72	54
	FhP+Viv reacted	70	59
**HW**	Initial sediment	25	26
	Reacted sediment	53	51
	Fh initial	0	14
	Fh reacted	74	60
	FhP reacted	61	61
	FhP+Viv reacted	81	62
**FKS**	Initial sediment	32	33
	Reacted sediment	42	46
	Fh initial	2	14
	Fh reacted	51	43
	FhP reacted	48	41
	FhP+Viv reacted	38	42

S4.7 P K-edge XAS

Table S22 shows the 10 groups of references that were pre-defined for the LCF of P K-edge X-ray absorption near-edge structure spectroscopy (XANES).

Table S22 – Groups used for LCF of P K-edge XANES.

**Group**	**Reference spectra**	**Abbreviation**	**Source**
Organic P	Phytic acid	IHP	Prietzel et al.[27]
P associated ferric minerals	P adsorbed to Fh	P-Fh	Kubeneck et al.[17]
Ferrous phosphate minerals	Vivianite	Viv	Kubeneck et al.[17]
Mn,Mg-substituted ferrous phosphate mineral	Mn- and Mg-enriched vivianite	Mn-,Mg-Viv	Kubeneck et al.[17]
P adsorbed to Al-oxides	P sorbed onto Al_2O_3	P-Al_2O_3	Giguet et al.[12]
Aluminium phosphate	Cristobalite	$AlPO_4$	Prietzel et al.[27]
Crystalline Ca-phosphate minerals	Hydroxyapatite	Hy-Ap	Kim et al.[15]
Amorphous Ca-phosphate minerals	Beta-tricalcium phosphate	$CaPO_4$	Vogel et al.[42]
P adsorbed to Mn-oxides	P sorbed onto δ-MnO_2	P-MnOx	Rivard et al.[30]
Mg-phosphate minerals	Trimagnesium phosphate octahydrate	MgP	Vogel et al.[42]

P K-edge XANES data was collected for initial and reacted FhP samples and controls (unamended reacted sediment). In initial FhP samples from all field sites, P was mainly associated with ferric minerals modeled by P adsorbed to ferrihydrite (P-Fh, Fig. S32, Table S23), as well as P associated with aluminum and calcium and organic P. The fraction of P associated with ferric minerals decreased with incubation at HSF (61 to 16%), while P was fitted to be present vivianite (19%). In the control sample (unamended reacted sediment) at HSF, P was fitted to be present as organic P (29%), vivianite (24%), P associated with Mn-oxides (9%) and aluminum (19%) and calcium (20%, Table S23). After field incubation of FhP samples at HW, no ferric mineral-associated P was present; instead, vivianite (24%) was fitted and the fraction of organic P increased (51%). The control samples (unamended reacted sediment) also contained P mainly associated with vivianite (21%) and organic P (49%). In reacted FhP samples of FKS, the fraction of P associated with ferric minerals decreased from 69% to 37%. In contrast to the other two field sites, P was not found to be bound with Mn-oxides or vivianite. However, the fraction of P associated with calcium increased, potentially suggesting a sink-switch from Fe-bound P to Ca-bound P.

Table S23 – Results of LCF of P K-edge XANES data. Abbreviations: P-Fh = Fh with adsorbed P; $AlPO_4$ = cristobalite (crystalline Al-phosphate mineral); P-Al_2O_3 = P adsorbed to Al_2O_3; Hy-Ap = hydroxyapatite; P-MnOx = P adsorbed to δ-MnO_2; Viv = vivianite; $CaPO_4$ = beta-tricalcium phosphate; IHP = phytic acid. NSSR = Normalized sum of squared residuals ($\sum_i(\mathrm{data}_i - \mathrm{fit}_i)^2 / \sum_i \mathrm{data}^2$); Red. $\chi^2 = (N_{idp}/N_{pts}) \sum_i(\mathrm{data}_i - \mathrm{fit}_i)/\varepsilon_i)^2 (N_{idp} - N_{var})^{-1}$. N_{idp}, N_{pts}, and N_{var} are, respectively, the number of independent data points in the model fit (86), the total number of data points (193), and the number of fitted variables (4-5).

		**P-Fh** [%]	**$AlPO_4$** [%]	**P-Al_2O_3** [%]	**Hy-Ap** [%]	**P-MnOx** [%]	**Viv** [%]	**$CaPO_4$** [%]	**IHP** [%]	**NSSR** [%]	**Red. χ^2**
	FhP_initial	61	18		9				12	0.1	0.001
**HSF**	FhP_reacted	16	21		7		19		38	0.1	0.001
	Sediment_reacted		19		20	9	24		29	0.1	0.001
	FhP_initial	60	18		6				16	0.1	0.001
**HW**	FhP-reacted		17		8		24		51	0.1	0.001
	Sediment_reacted		13		17		21		49	0.1	0.001
	FhP_initial	69	16		7			8		0.2	0.001
**FKS**	FhP_reacted	37		6	22				35	0.2	0.022
	Sediment_reacted			27				49	24	0.7	0.005

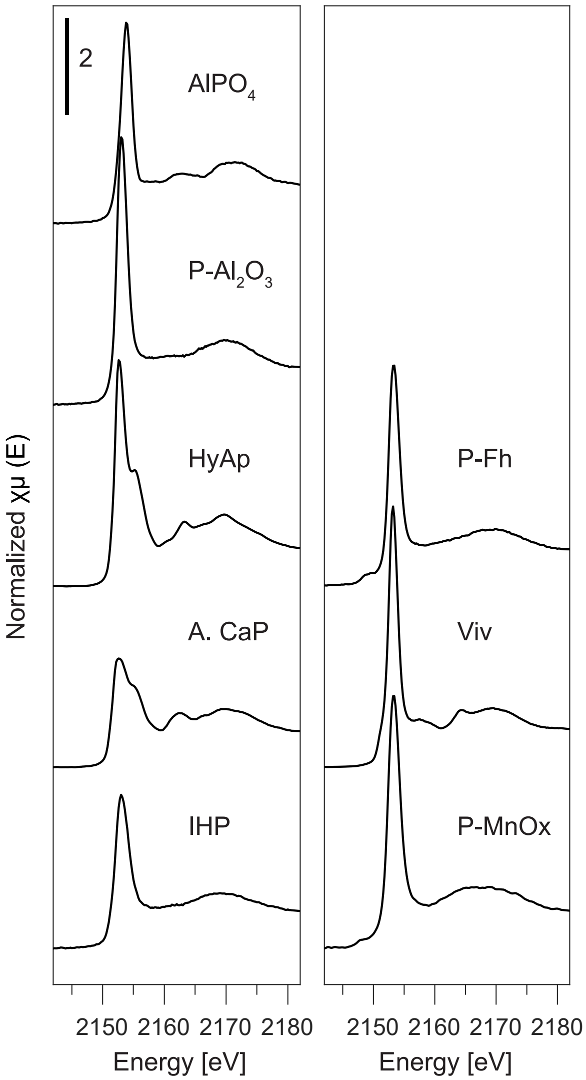

Figure S31 – Reference spectra used for LCF of the P K-edge XANES. For further information on reference material see Table S22.

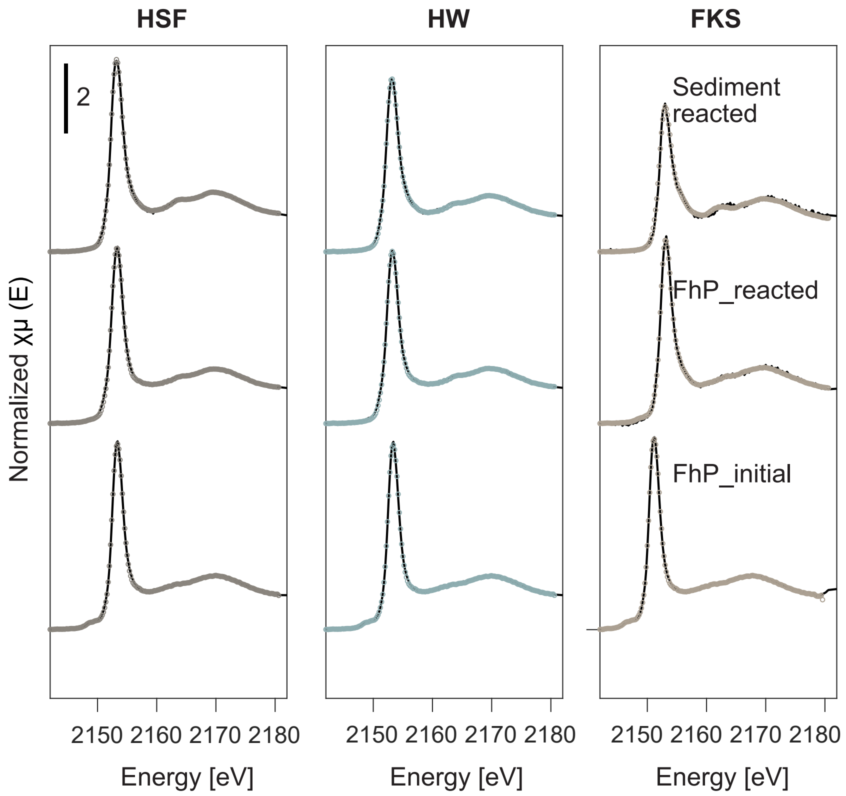

Figure S32 – P K-edge XANES spectra of reacted sediment (control) and initial and reacted FhP samples for the three field sites. Solid lines indicate experimental data and dotted lines show the LCF. Results of the LCF are presented in Table S23.

S5 Additional Mössbauer data - initial samples

Initial samples (mineral-sediment mixes) exhibited similar Mössbauer spectra since ≥ 95% of the Mössbauer signal originated from the added ^{57}Fe-labeled ferrihydrite (Fig. S33-S34). At 77 K, the Mössbauer spectra of initial Fh and FhP(+Viv) samples consisted of a doublet (Fe(III)D) with a center shift (CS) of ∼ 0.45 mm s^{-1} and quadrupole splitting (QS) of ∼ 0.9 mm s^{-1} (Table S24-S32). These values were slightly larger than reported ferrihydrite values (QS ∼ 0.70 mm s^{-1} e.g. ref[44], yet consistent with ferrihydrite starting to undergo magnetic ordering.[23] Below and at 25 K, a sextet (Fe(III)S1) emerged (CS: ∼ 0.47 mm s^{-1}, quadrupole shift (ε): ∼ -0.02 mm s^{-1}, H: ∼ 47.8-49.0 T; Table S24-S32), consistent with reported parameters for ferrihydrite.[36,44] In the initial FhP(+Viv) samples, the sextet at 25 K was not completely ordered yet (Figure S33-S34), possibly indicating a slight decrease in crystallinity due to phosphate adsorption. Fe(II) covered by the spectral area of Fe(II)D likely originated from the initial sediment. The hyperfine parameters are consistent with Fe(II) bound in phyllosilicate clays.[40]

Table S24 – Hyperfine parameters obtained for fitting of initial HSF Fh sample at 77 K, 25 K, 13 K and 10 K using xVBF model and 5 K using Full Static Hamiltonian model (FSH).

Temp.	Phase	Spectral Area %	CS^a [mm s^{-1}]	σ^b [mm s^{-1}]	QS^c or ε [mm s^{-1}]	prob. of cp.^{d}	σ^e [mm s^{-1} or[T]	H^f [T]	σ^g [T]	e2qQ/2^h [mm s^{-1}]	η^i [mm s^{-1}]	w^j [mm s^{-1}]	φ^k [°]	ϑ^l [°]	Red. $\chi^{2,m}$
77 K	Fe(III) D cp. 1	98.1	0.45		0.84	0.58			0.5						0.93
	Fe(III) D cp. 2				1.32	0.42			1.7						
	Fe(II) D	1.9	1.19		3.06				0.14						
25 K	Fe(III) D	3.7	0.44		0.71				0.2						5.66
	Fe(II) D	2.0	1.28						0.3						
	Fe(III) S1	94.2	0.47	0.1	-0.01		0.26	46.09	3.52						
13 K	Fe(III) D	3.8	0.44		0.71		0.2								1.26
	Fe(II) D	1.9	1.28		3.03		0.3								
	Fe(III) S1	96.4	0.47	0.1	-0.01		0.19	48.16	2.63						
10 K	Fe(III) D	3.1	0.44		0.71		0.2								0.91
	Fe(II) D	2.3	1.28		3.03		0.3								
	Fe(III) S1	94.7	0.47	0.1	-0.01		0.19	48.16	2.63						
5 K	Fe(III) D	4.3	0.44							0.71	0	0.3	0	0	64.24
	Fe(III) S1	95.8	0.48					49.06		-0.02	0	0.3	0	0	

[a]Center shift;
[b]Standard deviation of CS;
[c]Quadrupole splitting (QS, for doublets) or Quadrupole shift (ε, for sextet);
[d]For some broad doublets, we deployed a two-component fit to describe the population distribution. prob. of cp. provides the relative contribution of each component;
[e]Standard deviation of QS (doublets) or H (sextet);
[f]Hyperfine field;
[g]Standard deviation of H;
[h]Quadrupole splitting in paramagnetic state or quadrupole shift in FSH model;
[i]Asymmetry parameter;
[j]Half line width at half maximum;
[k]Azimuthal angle between the electric field gradient axis of symmetry with hyperfine field H;
[l]Polar angle between the electric field gradient axis of symmetry with hyperfine field H;
[m]Goodness of fit;
Abbreviations: Temp.= Temperature; Fe(III) D = Fe(III) doublet; Fe(II) D1= Fe(II) doublet; Fe(III) S1= Fe(III) sextet.

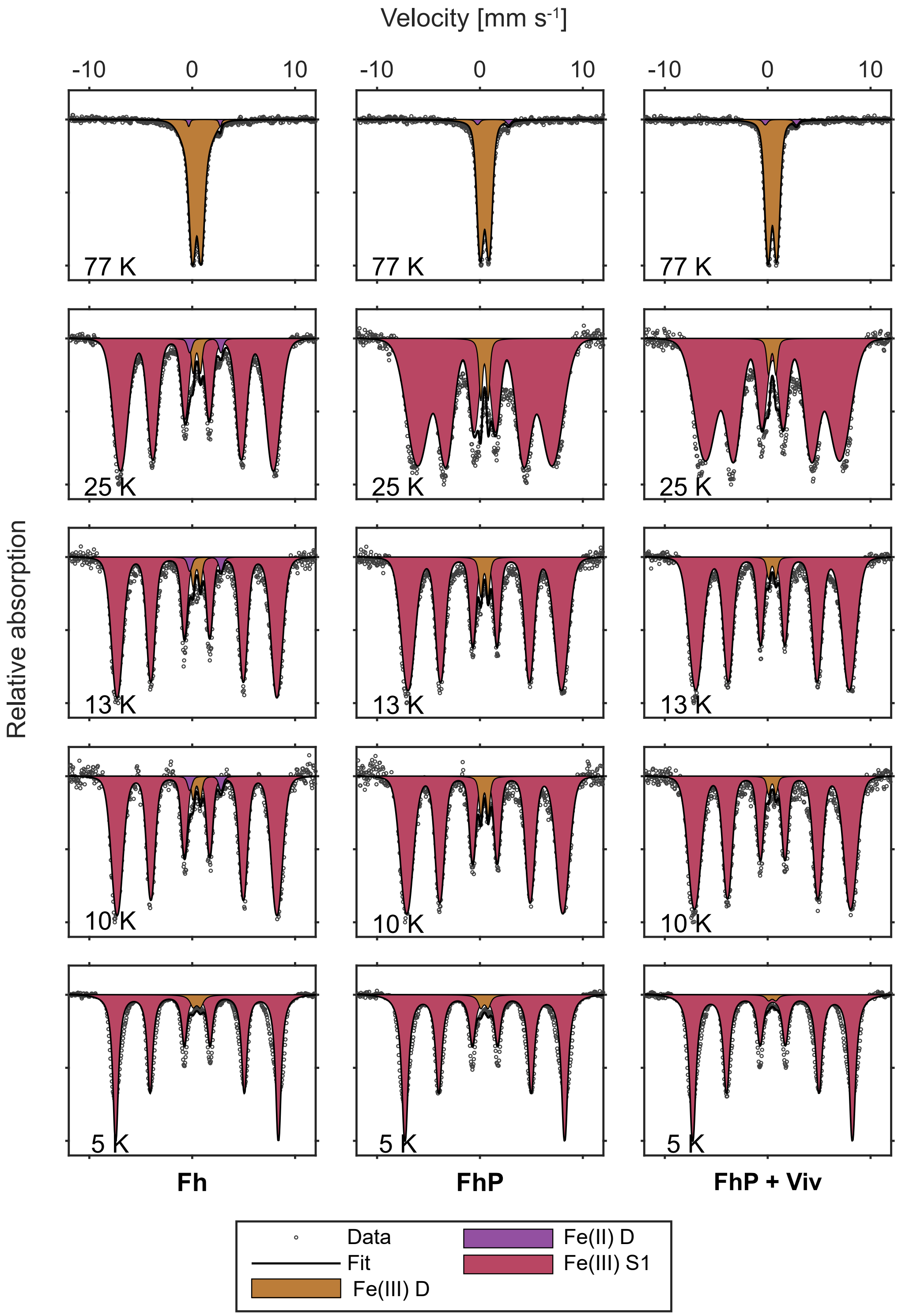

Figure S33 – Fitted Mössbauer spectra of initial HSF samples.

Table S25 – Hyperfine parameters obtained for fitting of HSF FhP sample at 77 K, 25 K, 13 K and 10 K using xVBF model and 5 K using Full Static Hamiltonian model (FSH).

Temp.	Phase	Spectral Area %	CS[a] [mm s^{-1}]	σ[b] [mm s^{-1}]	QS[c] or ε [mm s^{-1}]	σ[d] [mm s^{-1}] or[T]	H[e] [T]	σ[f] [T]	e2qQ/2[g] [mm s^{-1}]	η[h] [mm s^{-1}]	w[i] [mm s^{-1}]	φ[j] [°]	ϑ[k] [°]	Red.$\chi^{2,l}$
77 K	Fe(III) D	97.1	0.46		0.85			0.60						1.01
	Fe(II) D	2.9	1.28		3.03			0.30						
25 K	Fe(III) D	4.7	0.44		0.71			0.20						4.05
	Fe(III) S1	95.3	0.48	0.1	-0.003	0.36	40.54	5.77						
13 K	Fe(III) D	4.5	0.44		0.71	0.20								1.41
	Fe(III) S1	95.5	0.48	0.1	-0.007	0.20	46.27	3.39						
10 K	Fe(III) D	4.7	0.44		0.71	0.30								4.42
	Fe(III) S1	95.3	0.48	0.1	-0.009	0.20	47.05	3.14						
5 K	Fe(III) D	2.9	0.44						0.71	0	0.30	0	0	53.9
	Fe(III) S1	97.1	0.48				48.13		-0.02	0	0.35	0	0	

[a]Center shift;
[b]Standard deviation of CS;
[c]Quadrupole splitting (QS, for doublets) or Quadrupole shift (ε, for sextet);
[d]Standard deviation of QS (doublets) or H (sextet);
[e]Hyperfine field;
[f]Standard deviation of H;
[g]Quadrupole splitting in paramagnetic state or quadrupole shift in FSH model;
[h]Asymmetry parameter;
[i]Half line width at half maximum;
[j]Azimuthal angle between the electric field gradient axis of symmetry with hyperfine field H;
[k]Polar angle between the electric field gradient axis of symmetry with hyperfine field H;
[l]Goodness of fit;
Abbreviations: Temp.= Temperature; Fe(III) D = Fe(III) doublet; Fe(II) D1= Fe(II) doublet; Fe(III) S1= Fe(III) sextet.

Table S26 – Hyperfine parameters obtained for fitting of HSF FhP+Viv sample at 77 K, 25 K, 13 K and 10 K using xVBF model and 5 K using Full Static Hamiltonian model (FSH).

**Temp.**	**Phase**	**Spectral Area %**	**CS[a] [mm s^{-1}]**	**σ[b] [mm s^{-1}]**	**QS[c] or ε [mm s^{-1}]**	**σ[d] [mm s^{-1}] or[T]**	**H[e] [T]**	**σ[f] [T]**	**e2qQ/2[g] [mm s^{-1}]**	**η[h] [mm s^{-1}]**	**w[i] [mm s^{-1}]**	**φ[j] [°]**	**ϑ[k] [°]**	**Red.χ[2,l]**
77 K	Fe(III) D	97.0	0.46		0.85			0.48						1.01
	Fe(II) D	3.0	1.28		3.03			0.30						
25 K	Fe(III) D	2.90	0.44		0.71			0.20						4.57
	Fe(III) S1	97.1	0.48	0.1	-0.003	0.36	40.54	5.77						
13 K	Fe(III) D	2.6	0.44		0.71	0.20								1.91
	Fe(III) S1	97.5	0.48	0.1	-0.007	0.20	46.27	3.39						
10 K	Fe(III) D	2.3	0.44		0.71	0.30								1.13
	Fe(III) S1	97.7	0.48	0.1	-0.009	0.20	47.05	3.14						
5 K	Fe(III) D	1.6	0.44						0.71	0	0.30	0	0	28.81
	Fe(III) S1	98.4	0.48				48.13		-0.02	0	0.35	0	0	

[a]Center shift;

[b]Standard deviation of CS;

[c]Quadrupole splitting (QS, for doublets) or Quadrupole shift (ε, for sextet);

[d]Standard deviation of QS (doublets) or H (sextet);

[e]Hyperfine field;

[f]Standard deviation of H;

[g]Quadrupole splitting in paramagnetic state or quadrupole shift in FSH model;

[h]Asymmetry parameter;

[i]Half line width at half maximum;

[j]Azimuthal angle between the electric field gradient axis of symmetry with hyperfine field H;

[k]Polar angle between the electric field gradient axis of symmetry with hyperfine field H;

[l]Goodness of fit;

Abbreviations: Temp.= Temperature; Fe(III) D = Fe(III) doublet; Fe(II) D1= Fe(II) doublet; Fe(III) S1= Fe(III) sextet.

Table S27 – Hyperfine parameters obtained for fitting of initial HW Fh sample at 77 K, 25 K, 13 K and 10 K using xVBF model and 5 K using Full Static Hamiltonian model (FSH).

**Temp.**	**Phase**	**Spectral Area %**	**CS[a] [mm s^{-1}]**	**σ[b] [mm s^{-1}]**	**QS[c] or ε [mm s^{-1}]**	**prob. of cp.[d]**	**σ[e] [mm s^{-1}] or[T]**	**H[f] [T]**	**σ[g] [T]**	**e2qQ/2[h] [mm s^{-1}]**	**η[i] [mm s^{-1}]**	**w[j] [mm s^{-1}]**	**φ[k] [°]**	**ϑ[l] [°]**	**Red. $\chi^{2,m}$**
77 K	Fe(III) D cp. 1	100	0.45		0.84	0.60			0.50						0.97
	Fe(III) D cp. 2				1.32	0.40			1.70						
25 K	Fe(III) D	3.6	0.44		0.71				0.20						5.86
	Fe(III) S1	96.4	0.47	0.01	-0.01		0.26	46.09	3.52						
13 K	Fe(III) D	3.3	0.44		0.71		0.2								3.31
	Fe(III) S1	96.4	0.47	0.1	-0.01		0.19	48.16	2.63						
10 K	Fe(III) D	3.7	0.44		0.71		0.2								2.33
	Fe(III) S1	96.3	0.47	0.1	-0.01		0.19	48.16	2.63						
5 K	Fe(III) D	3.4	0.44							0.71	0	0.3	0	0	140
	Fe(III) S1	96.7	0.48					49.06		-0.02	0	0.3	0	0	

[a]Center shift;

[b]Standard deviation of CS;

[c]Quadrupole splitting (QS, for doublets) or Quadrupole shift (ε, for sextet);

[d]For some broad doublets, we deployed a two-component fit to describe the population distribution. prob. of cp. provides the relative contribution of each component;

[e]Standard deviation of QS (doublets) or H (sextet);

[f]Hyperfine field;

[g]Standard deviation of H;

[h]Quadrupole splitting in paramagnetic state or quadrupole shift in FSH model;

[i]Asymmetry parameter;

[j]Half line width at half maximum;

[k]Azimuthal angle between the electric field gradient axis of symmetry with hyperfine field H;

[l]Polar angle between the electric field gradient axis of symmetry with hyperfine field H;

[m]Goodness of fit;

Abbreviations: Temp.= Temperature; Fe(III) D = Fe(III) doublet; Fe(II) D1= Fe(II) doublet; Fe(III) S1= Fe(III) sextet.

Table S28 – Hyperfine parameters obtained for fitting of HW FhP sample at 77 K, 25 K, 13 K and 10 K using xVBF model and 5 K using Full Static Hamiltonian model (FSH).

**Temp.**	**Phase**	**Spectral Area %**	**CS[a]** [mm s^{-1}]	σ**[b]** [mm s^{-1}]	**QS[c] or** ε [mm s^{-1}]	σ**[d]** [mm s^{-1}] or[T]	**H[e]** [T]	σ**[f]** [T]	**e2qQ/2[g]** [mm s^{-1}]	η**[h]** [mm s^{-1}]	**w[i]** [mm s^{-1}]	φ**[j]** [°]	ϑ**[k]** [°]	**Red.**χ^{2}**[,l]**
77 K	Fe(III) D	100	0.46		0.85			0.61						1.01
25 K	Fe(III) D	4.4	0.44		0.71			0.20						5.55
	Fe(III) S1	95.6	0.48	0.1	-0.003	0.36	40.54	5.77						
13 K	Fe(III) D	4.2	0.44		0.71	0.20								1.87
	Fe(III) S1	95.8	0.48	0.1	-0.007	0.20	46.27	3.39						
10 K	Fe(III) D	3.7	0.44		0.71	0.20								2.33
	Fe(III) S1	96.3	0.48	0.1	-0.009	0.20	47.05	3.14						
5 K	Fe(III) D	2.9	0.44						0.71	0	0.30	0	0	53.9
	Fe(III) S1	97.1	0.48				48.13		-0.02	0	0.35	0	0	

[a]Center shift;
[b]Standard deviation of CS;
[c]Quadrupole splitting (QS, for doublets) or Quadrupole shift (ε, for sextet);
[d]Standard deviation of QS (doublets) or H (sextet);
[e]Hyperfine field;
[f]Standard deviation of H;
[g]Quadrupole splitting in paramagnetic state or quadrupole shift in FSH model;
[h]Asymmetry parameter;
[i]Half line width at half maximum;
[j]Azimuthal angle between the electric field gradient axis of symmetry with hyperfine field H;
[k]Polar angle between the electric field gradient axis of symmetry with hyperfine field H;
[l]Goodness of fit;
Abbreviations: Temp.= Temperature; Fe(III) D = Fe(III) doublet; Fe(II) D1= Fe(II) doublet; Fe(III) S1= Fe(III) sextet.

Table S29 – Hyperfine parameters obtained for fitting of HW FhP+Viv sample at 77 K, 25 K, 13 K and 10 K using xVBF model and 5 K using Full Static Hamiltonian model (FSH).

**Temp.**	**Phase**	**Spectral Area %**	**CS[a]** [mm s^{-1}]	σ[b] [mm s^{-1}]	**QS[c] or ε** [mm s^{-1}]	σ[d] [mm s^{-1}] or[T]	**H[e]** [T]	σ[f] [T]	**e2qQ/2[g]** [mm s^{-1}]	η[h] [mm s^{-1}]	**w[i]** [mm s^{-1}]	φ[j] [°]	ϑ[k] [°]	**Red.** $\chi^{2,l}$
77 K	Fe(III) D	100	0.46		0.85			0.61						1.87
25 K	Fe(III) D	2.8	0.44		0.71			0.20						13.9
	Fe(III) S1	97.2	0.48	0.1	-0.003	0.36	41.76	5.24						
13 K	Fe(III) D	2.3	0.44		0.71	0.20								5.43
	Fe(III) S1	97.7	0.47	0.1	-0.007	0.20	46.29	3.26						
10 K	Fe(III) D	1.9	0.44		0.71	0.20								3.95
	Fe(III) S1	98.1	0.47	0.1	-0.009	0.20	46.92	3.26						
5 K	Fe(III) D	1.1	0.44						0.71	0	0.30	0	0	163.14
	Fe(III) S1	98.9	0.47				47.78		-0.02	0	0.35	0	0	

[a]Center shift;

[b]Standard deviation of CS;

[c]Quadrupole splitting (QS, for doublets) or Quadrupole shift (ε, for sextet);

[d]Standard deviation of QS (doublets) or H (sextet);

[e]Hyperfine field;

[f]Standard deviation of H;

[g]Quadrupole splitting in paramagnetic state or quadrupole shift in FSH model;

[h]Asymmetry parameter;

[i]Half line width at half maximum;

[j]Azimuthal angle between the electric field gradient axis of symmetry with hyperfine field H;

[k]Polar angle between the electric field gradient axis of symmetry with hyperfine field H;

[l]Goodness of fit;

Abbreviations: Temp.= Temperature; Fe(III) D = Fe(III) doublet; Fe(II) D1= Fe(II) doublet; Fe(III) S1= Fe(III) sextet.

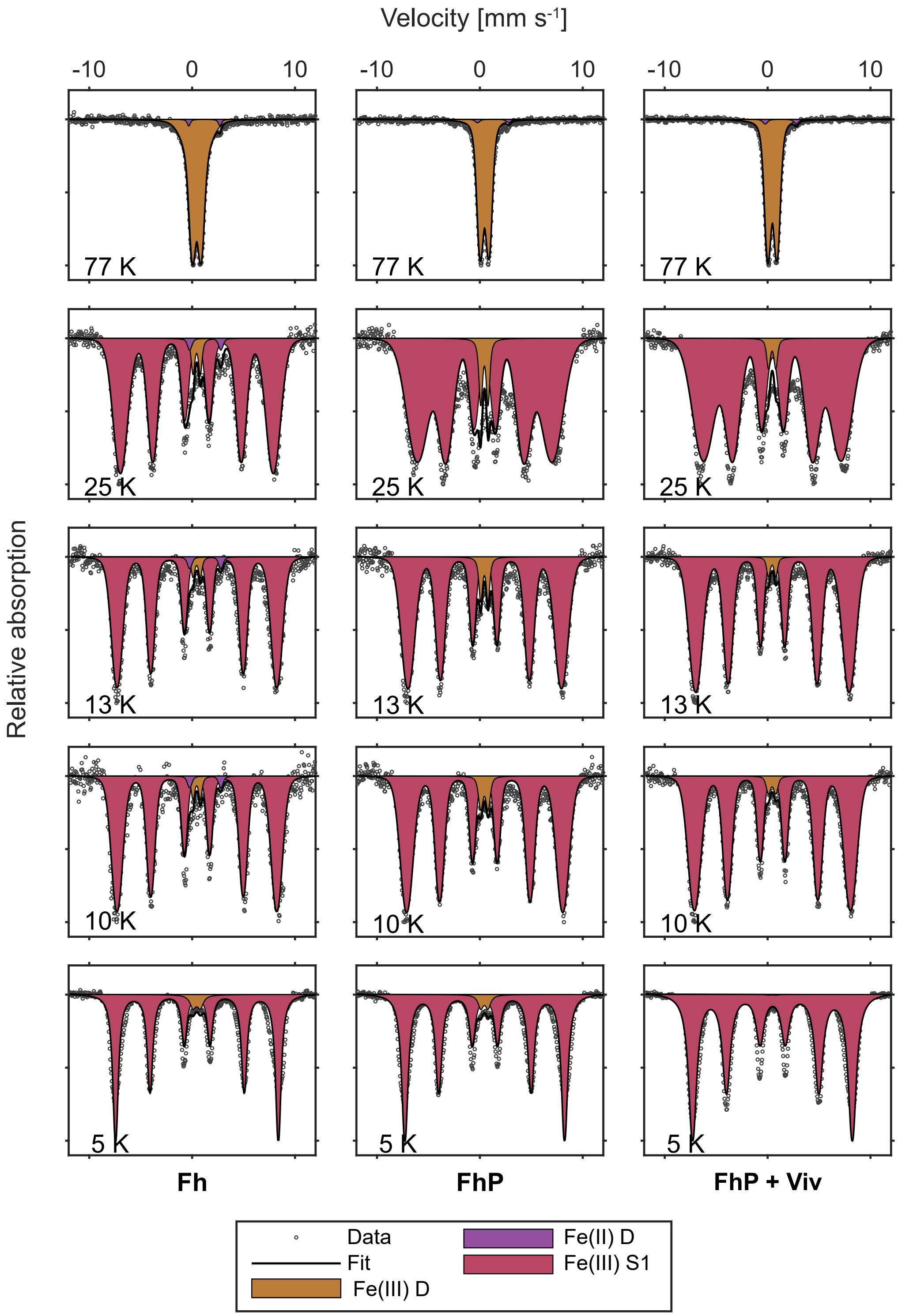

Figure S34 – Fitted Mössbauer spectra of initial FKS samples.

Table S30 – Hyperfine parameters obtained for fitting of initial FKS Fh sample at 77 K, 25 K, 13 K and 10 K using xVBF model and 5 K using Full Static Hamiltonian model (FSH).

Temp.	Phase	Spectral Area %	CS[a] [mm s^{-1}]	σ[b] [mm s^{-1}]	QS[c] or ε [mm s^{-1}]	prob. of cp.[d]	σ[e] [mm s^{-1}] or[T]	H[f] [T]	σ[g] [T]	e2qQ/2[h] [mm s^{-1}]	η[i] [mm s^{-1}]	w[j] [mm s^{-1}]	φ[k] [°]	ϑ[l] [°]	Red. $\chi^{2,m}$
77 K	Fe(III) D cp. 1	97.7	0.45		0.84	0.64			0.5						1.03
	Fe(III) D cp. 2				1.32	0.36			1.7						
	Fe(II) D	2.3	1.19		3.06				0.14						
25 K	Fe(III) D	3.7	0.44		0.71				0.2						1.17
	Fe(II) D	3.0	1.28		3.03				0.3						
	Fe(III) S1	93.3	0.47	0.1	-0.01		0.26	46.09	3.52						
13 K	Fe(III) D	2.8	0.44		0.71		0.2								0.95
	Fe(II) D	1.9	1.28		3.03		0.3								
	Fe(III) S1	95.4	0.47	0.1	-0.01		0.19	48.16	2.63						
10 K	Fe(III) D	3.1	0.44		0.71		0.2								0.76
	Fe(II) D	1.9	1.28		3.03		0.3								
	Fe(III) S1	95.0	0.47	0.1	-0.01		0.19	48.16	2.63						
5 K	Fe(III) D	4.7	0.44							0.71	0	0.3	0	0	19.81
	Fe(III) S1	95.3	0.48					49.06		-0.02	0	0.3	0	0	

[a]Center shift;
[b]Standard deviation of CS;
[c]Quadrupole splitting (QS, for doublets) or Quadrupole shift (ε, for sextet);
[d]For some broad doublets, we deployed a two-component fit to describe the population distribution. prob. of cp. provides the relative contribution of each component;
[e]Standard deviation of QS (doublets) or H (sextet);
[f]Hyperfine field;
[g]Standard deviation of H;
[h]Quadrupole splitting in paramagnetic state or quadrupole shift in FSH model;
[i]Asymmetry parameter;
[j]Half line width at half maximum;
[k]Azimuthal angle between the electric field gradient axis of symmetry with hyperfine field H;
[l]Polar angle between the electric field gradient axis of symmetry with hyperfine field H;
[m]Goodness of fit;
Abbreviations: Temp.= Temperature; Fe(III) D = Fe(III) doublet; Fe(II) D1= Fe(II) doublet; Fe(III) S1= Fe(III) sextet.

Table S31 – Hyperfine parameters obtained for fitting of FKS FhP sample at 77 K, 25 K, 13 K and 10 K using xVBF model and 5 K using Full Static Hamiltonian model (FSH).

**Temp.**	**Phase**	**Spectral Area %**	**CS[a] [mm s^{-1}]**	**σ[b] [mm s^{-1}]**	**QS[c] or ε [mm s^{-1}]**	**σ[d] [mm s^{-1}] or[T]**	**H[e] [T]**	**σ[f] [T]**	**e2qQ/2[g] [mm s^{-1}]**	**η[h] [mm s^{-1}]**	**w[i] [mm s^{-1}]**	**φ[j] [°]**	**ϑ[k] [°]**	**Red.$\chi^{2,l}$**
77 K	Fe(III) D	98.1	0.46		0.85			0.50						1.20
	Fe(II) D	1.9	1.28		3.03			0.30						
25 K	Fe(III) D	5.2	0.44		0.71			0.20						2.98
	Fe(III) S1	94.8	0.48	0.1	-0.003	0.36	40.54	5.77						
13 K	Fe(III) D	5.2	0.44		0.71	0.20								1.19
	Fe(III) S1	94.8	0.48	0.1	-0.007	0.20	46.27	3.39						
10 K	Fe(III) D	4.6	0.44		0.71	0.30								1.16
	Fe(III) S1	95.4	0.48	0.1	-0.009	0.20	47.05	3.14						
5 K	Fe(III) D	3.7	0.44						0.71	0	0.30	0	0	23.50
	Fe(III) S1	96.3	0.48				48.13		-0.02	0	0.35	0	0	

[a]Center shift;
[b]Standard deviation of CS;
[c]Quadrupole splitting (QS, for doublets) or Quadrupole shift (ε, for sextet);
[d]Standard deviation of QS (doublets) or H (sextet);
[e]Hyperfine field;
[f]Standard deviation of H;
[g]Quadrupole splitting in paramagnetic state or quadrupole shift in FSH model;
[h]Asymmetry parameter;
[i]Half line width at half maximum;
[j]Azimuthal angle between the electric field gradient axis of symmetry with hyperfine field H;
[k]Polar angle between the electric field gradient axis of symmetry with hyperfine field H;
[l]Goodness of fit;
Abbreviations: Temp.= Temperature; Fe(III) D = Fe(III) doublet; Fe(II) D1= Fe(II) doublet; Fe(III) S1= Fe(III) sextet.

Table S32 – Hyperfine parameters obtained for fitting of FKS FhP+Viv sample at 77 K, 25 K, 13 K and 10 K using xVBF model and 5 K using Full Static Hamiltonian model (FSH).

**Temp.**	**Phase**	**Spectral Area %**	**CS[a] [mm s^{-1}]**	**σ[b] [mm s^{-1}]**	**QS[c] or ε [mm s^{-1}]**	**σ[d] [mm s^{-1}] or[T]**	**H[e] [T]**	**σ[f] [T]**	**e2qQ/2[g] [mm s^{-1}]**	**η[h] [mm s^{-1}]**	**w[i] [mm s^{-1}]**	**φ[j] [°]**	**ϑ[k] [°]**	**Red.χ^{2}[,l]**
77 K	Fe(III) D	97.4	0.47		0.89			0.50						1.40
	Fe(II) D	2.6	1.28		3.03			0.30						
25 K	Fe(III) D	2.5	0.44		0.71			0.20						5.73
	Fe(III) S1	97.5	0.48	0.1	-0.003	0.36	40.54	5.77						
13 K	Fe(III) D	2.3	0.44		0.71	0.20								1.83
	Fe(III) S1	97.7	0.48	0.1	-0.007	0.20	46.27	3.39						
10 K	Fe(III) D	2.5	0.44		0.71	0.30								2.06
	Fe(III) S1	97.5	0.48	0.1	-0.009	0.20	47.05	3.14						
5 K	Fe(III) S1	100	0.48				48.13		-0.02	0	0.35	0	0	23.50

[a]Center shift;
[b]Standard deviation of CS;
[c]Quadrupole splitting (QS, for doublets) or Quadrupole shift (ε, for sextet);
[d]Standard deviation of QS (doublets) or H (sextet);
[e]Hyperfine field;
[f]Standard deviation of H;
[g]Quadrupole splitting in paramagnetic state or quadrupole shift in FSH model;
[h]Asymmetry parameter;
[i]Half line width at half maximum;
[j]Azimuthal angle between the electric field gradient axis of symmetry with hyperfine field H;
[k]Polar angle between the electric field gradient axis of symmetry with hyperfine field H;
[l]Goodness of fit;
Abbreviations: Temp.= Temperature; Fe(III) D = Fe(III) doublet; Fe(II) D1= Fe(II) doublet; Fe(III) S1= Fe(III) sextet.

References

[1] Blaes, N., Fischer, H., Gonser, U., 1985. Analytical expression for the Mössbauer line shape of ^{57}Fe in the presence of mixed hyperfine interactions. Nucl. Instrum. Methods Phys. Res. 9, 201–208.

[2] Borch, T., Masue, Y., Kukkadapu, R.K., Fendorf, S., 2007. Phosphate imposed limitations on biological reduction and alteration of ferrihydrite. Environ. Sci. Technol. 41, 166–172.

[3] Brady, M.P., Tostevin, R., Tosca, N.J., 2022. Marine phosphate availability and the chemical origins of life on Earth. Nat. Commun. 13, 5162.

[4] Canfield, D.E., 1989. Reactive iron in marine sediments. Geochim. Cosmochim. Acta 53, 619–632.

[5] Claff, S.R., Sullivan, L.A., Burton, E.D., Bush, R.T., 2010. A sequential extraction procedure for acid sulfate soils: Partitioning of iron. Geoderma 155, 224–230.

[6] Cline, J.D., 1969. Spectrophotometric determination of hydrogen sulfide in natural waters 1. Limnol. Oceanogr. 14, 454–458.

[7] Frederichs, T., von Dobeneck, T., Bleil, U., Dekkers, M.J., 2003. Towards the identification of siderite, rhodochrosite, and vivianite in sediments by their low-temperature magnetic properties. Phys. Chem. Earth 28, 669–679.

[8] Fredrickson, J.K., Zachara, J.M., Kennedy, D.W., Dong, H., Onstott, T.C., Hinman, N.W., Li, S., 1998. Biogenic iron mineralization accompanying the dissimilatory reduction of hydrous ferric oxide by a groundwater bacterium. Geochim. Cosmochim. Acta 62, 3239–3257.

[9] Frommer, J., Voegelin, A., Dittmar, J., Marcus, M.A., Kretzschmar, R., 2011. Biogeochemical processes and arsenic enrichment around rice roots in paddy soil: Results from micro-focused X-ray spectroscopy. Eur. J. Soil Sci. 62, 305–317.

[10] Génin, J.M.R., Abdelmoula, M., Ruby, C., Upadhyay, C., 2006. Speciation of iron; characterisation and structure of green rusts and $Fe^{II–III}$ oxyhydroxycarbonate fougerite. C. R. Geosci. 338, 402–419.

[11] Génin, J.M.R., Bourrié, G., Trolard, F., Abdelmoula, M., Jaffrezic, A., Refait, P., Maitre, V., Humbert, B., Herbillon, A., 1998. Thermodynamic equilibria in aqueous suspensions of synthetic and natural Fe(II)- Fe(III) green rusts: Occurrences of the mineral in hydromorphic soils. Environ. Sci. Technol. 32, 1058–1068.

[12] Giguet-Covex, C., Poulenard, J., Chalmin, E., Arnaud, F., Rivard, C., Jenny, J.P., Dorioz, J.M., 2013. XANES spectroscopy as a tool to trace phosphorus transformation during soil genesis and mountain ecosystem development from lake sediments. Geochim. Cosmochim. Acta 118, 129–147.

[13] Ginn, B., Meile, C., Wilmoth, J., Tang, Y., Thompson, A., 2017. Rapid iron reduction rates are stimulated by high-amplitude redox fluctuations in a tropical forest soil. Environ. Sci. Technol. 51, 3250–3259.

[14] Kelly, S., Hesterberg, D., Ravel, B., 2008. Analysis of soils and minerals using X-ray absorption spectroscopy. Methods of soil analysis part 5—mineralogical methods 5, 387–463.

[15] Kim, B., Gautier, M., Rivard, C., Sanglar, C., Michel, P., Gourdon, R., 2015. Effect of aging on phosphorus speciation in surface deposit of a vertical flow constructed wetland. Environ. Sci. Technol. 49, 4903–4910.

[16] Kraal, P., van Genuchten, C.M., Behrends, T., Rose, A.L., 2019. Sorption of phosphate and silicate alters dissolution kinetics of poorly crystalline iron (oxyhydr)oxide. Chemosphere 234, 690–701.

[17] Kubeneck, L.J., Notini, L., Rothwell, K.A., Fantappiè, G., Huthwelker, T., ThomasArrigo, L.K., Kretzschmar, R., 2024. Transformation of vivianite in intertidal sediments with contrasting sulfide conditions. Geochim. Cosmochim. Acta 370, 173–187.

[18] Kubeneck, L.J., ThomasArrigo, L.K., Rothwell, K.A., Kaegi, R., Kretzschmar, R., 2023. Competitive incorporation of Mn and Mg in vivianite at varying salinity and effects on crystal structure and morphology. Geochim. Cosmochim. Acta 346, 231–244.

[19] Kukkadapu, R.K., Zachara, J.M., Fredrickson, J.K., Kennedy, D.W., 2004. Biotransformation of two-line silica-ferrihydrite by a dissimilatory Fe (III)-reducing bacterium: Formation of carbonate green rust in the presence of phosphate. Geochim. Cosmochim. Acta 68, 2799–2814.

[20] Lagarec, K., Rancourt, D., 1997. Extended Voigt-based analytic lineshape method for determining N-dimensional correlated hyperfine parameter distributions in Mössbauer spectroscopy. Nucl. Instrum. Methods Phys. Res. 129, 266–280.

[21] Langner, P., Mikutta, C., Kretzschmar, R., 2012. Arsenic sequestration by organic sulphur in peat. Nat. Geosci. 5, 66–73.

[22] Meijer, H., Van den Handel, J., Frikkee, E., 1967. Magnetic behaviour of vivianite, $Fe_3(PO_4)_2$ $8H_2O$. Phys. 34, 475–483.

[23] Notini, L., Schulz, K., Kubeneck, L.J., Grigg, A.R.C., Rothwell, K.A., Fantappiè, G., ThomasArrigo, L.K., Kretzschmar, R., 2023. A new approach for investigating iron mineral transformations in soils and sediments using ^{57}Fe-labeled minerals and ^{57}Fe Mössbauer spectroscopy. Environ. Sci. Technol. 57, 10008–10018.

[24] Notini, L., ThomasArrigo, L.K., Kaegi, R., Kretzschmar, R., 2022. Coexisting goethite promotes Fe (II)-catalyzed transformation of ferrihydrite to goethite. Environ. Sci. Technol. 56, 12723–12733.

[25] O'Loughlin, E.J., Boyanov, M.I., Gorski, C.A., Scherer, M.M., Kemner, K.M., 2021. Effects of Fe (III) oxide mineralogy and phosphate on Fe (II) secondary mineral formation during microbial iron reduction. Minerals 11, 149.

[26] Poulton, S.W., Canfield, D.E., 2005. Development of a sequential extraction procedure for iron: Implications for iron partitioning in continentally derived particulates. Chem. Geol. 214, 209–221.

[27] Prietzel, J., Harrington, G., Häusler, W., Heister, K., Werner, F., Klysubun, W., 2016. Reference spectra of important adsorbed organic and inorganic phosphate binding forms for soil P speciation using synchrotron-based K-edge XANES spectroscopy. J. Synchrotron Radiat. 23, 532–544.

[28] Rancourt, D., Ping, J., 1991. Voigt-based methods for arbitrary-shape static hyperfine parameter distributions in Mössbauer spectroscopy. Nucl. Instrum. Methods Phys. Res. 58, 85–97.

[29] Ravel, B., Newville, M., 2005. ATHENA, ARTEMIS, HEPHAESTUS: Data analysis for X-ray absorption spectroscopy using IFEFFIT. J. Synchrotron Rad. 12, 537–541.

[30] Rivard, C., Lanson, B., Cotte, M., 2016. Phosphorus speciation and micro-scale spatial distribution in North-American temperate agricultural soils from micro X-ray fluorescence and X-ray absorption near-edge spectroscopy. Plant and soil 401, 7–22.

[31] Rusch, B., Génin, J.M., Ruby, C., Abdelmoula, M., Bonville, P., 2008. Mössbauer study of magnetism in FeII-III (oxy-) hydroxycarbonate green rusts; ferrimagnetism of FeII- III hydroxycarbonate. Hyperfine Interact. 187, 7–12.

[32] Striggow, B., 2017. Field measurement of oxidation-reduction potential (ORP). US Environmental Protection Agency; Science and Ecosystem Support Division , 2017–07.

[33] Taylor, P., Maeck, R., De Bièvre, P., 1992. Determination of the absolute isotopic composition and atomic weight of a reference sample of natural iron. IJMS 121, 111–125.

[34] Thiel, J., Byrne, J.M., Kappler, A., Schink, B., Pester, M., 2019. Pyrite formation from FeS and H_2S is mediated through microbial redox activity. PNAS 116, 6897–6902.

[35] ThomasArrigo, L.K., Bouchet, S., Kaegi, R., Kretzschmar, R., 2020. Organic matter influences transformation products of ferrihydrite exposed to sulfide. Environ. Sci. Nano 7, 3405–3418.

[36] ThomasArrigo, L.K., Byrne, J.M., Kappler, A., Kretzschmar, R., 2018. Impact of organic matter on iron (II)-catalyzed mineral transformations in ferrihydrite–organic matter coprecipitates. Environ. Sci. Technol. 52, 12316–12326.

[37] ThomasArrigo, L.K., Mikutta, C., Byrne, J., Kappler, A., Kretzschmar, R., 2017. Iron (II)-catalyzed iron atom exchange and mineralogical changes in iron-rich organic freshwater flocs: An iron isotope tracer study. Environ. Sci. Technol. 51, 6897–6907.

[38] ThomasArrigo, L.K., Notini, L., Shuster, J., Nydegger, T., Vontobel, S., Fischer, S., Kappler, A., Kretzschmar, R., 2022. Mineral characterization and composition of Fe-rich flocs from wetlands of Iceland: Implications for Fe, C and trace element export. STOTEN 816, 151567.

[39] Van Genuchten, C., Behrends, T., Kraal, P., Stipp, S.L., Dideriksen, K., 2018. Controls on the formation of Fe (II,III)(hydr)oxides by Fe(0) electrolysis. Electrochim. Acta 286, 324–338.

[40] Vandenberghe, R.E., De Grave, E., 2012. Application of Mössbauer spectroscopy in earth sciences, in: Mössbauer Spectroscopy: Tutorial Book. Springer, pp. 91–185.

[41] Voelz, J.L., Johnson, N.W., Chun, C.L., Arnold, W.A., Penn, R.L., 2019. Quantitative dissolution of environmentally accessible iron residing in iron-rich minerals: A review. ACS Earth Space Chem. 3, 1371–1392.

[42] Vogel, C., Rivard, C., Wilken, V., Muskolus, A., Adam, C., 2018. Performance of secondary P-fertilizers in pot experiments analyzed by phosphorus X-ray absorption near-edge structure (XANES) spectroscopy. Ambio 47, 62–72.

[43] Webb, S., 2005. SIXpack: a graphical user interface for XAS analysis using IFEFFIT. Phys. Scr. 2005, 1011.

[44] Zhou, Z., Muehe, E.M., Tomaszewski, E.J., Lezama-Pacheco, J., Kappler, A., Byrne, J.M., 2020. Effect of natural organic matter on the fate of cadmium during microbial ferrihydrite reduction. Environ. Sci. Technol. 54, 9445–9453.
